# Using hybridization networks to retrace the evolution of Indo-European languages

**DOI:** 10.1186/s12862-016-0745-6

**Published:** 2016-09-06

**Authors:** Matthieu Willems, Etienne Lord, Louise Laforest, Gilbert Labelle, François-Joseph Lapointe, Anna Maria Di Sciullo, Vladimir Makarenkov

**Affiliations:** 1 Department of Computer Science, Université du Québec à Montréal, Case postale 8888, succursale Centre-ville, Montréal, Québec H3C 3P8 Canada; 2 Department of Biological Sciences, Université de Montréal, C.P. 6128 succ. Centre-Ville, Montreal, Quebec H3C 3J7 Canada; 3 Department of Mathematics, Université du Québec à Montréal, Case postale 8888, succursale Centre-ville, Montréal, Québec H3C 3P8 Canada; 4 Department of Linguistics, Université du Québec à Montréal, Case postale 8888, succursale Centre-ville, Montréal, Québec H3C 3P8 Canada

**Keywords:** Historical linguistics, Phylogenetic trees, Phylogenetic networks, Reticulate evolution

## Abstract

**Background:**

Curious parallels between the processes of species and language evolution have been observed by many researchers. Retracing the evolution of Indo-European (IE) languages remains one of the most intriguing intellectual challenges in historical linguistics. Most of the IE language studies use the traditional phylogenetic tree model to represent the evolution of natural languages, thus not taking into account reticulate evolutionary events, such as language hybridization and word borrowing which can be associated with species hybridization and horizontal gene transfer, respectively. More recently, implicit evolutionary networks, such as split graphs and minimal lateral networks, have been used to account for reticulate evolution in linguistics.

**Results:**

Striking parallels existing between the evolution of species and natural languages allowed us to apply three computational biology methods for reconstruction of phylogenetic networks to model the evolution of IE languages. We show how the transfer of methods between the two disciplines can be achieved, making necessary methodological adaptations. Considering basic vocabulary data from the well-known Dyen’s lexical database, which contains word forms in 84 IE languages for the meanings of a 200-meaning Swadesh list, we adapt a recently developed computational biology algorithm for building explicit hybridization networks to study the evolution of IE languages and compare our findings to the results provided by the split graph and galled network methods.

**Conclusion:**

We conclude that explicit phylogenetic networks can be successfully used to identify donors and recipients of lexical material as well as the degree of influence of each donor language on the corresponding recipient languages. We show that our algorithm is well suited to detect reticulate relationships among languages, and present some historical and linguistic justification for the results obtained. Our findings could be further refined if relevant syntactic, phonological and morphological data could be analyzed along with the available lexical data.

**Electronic supplementary material:**

The online version of this article (doi:10.1186/s12862-016-0745-6) contains supplementary material, which is available to authorized users.

## Background

Many curious similarities between the processes of species and language evolution have been observed since Darwin’s The Descent of Man [1]. But even earlier, in 1863, August Schleicher [2] sent a letter to Ernst Haeckel in which he discussed some of these similarities, comparing, for example, mixed languages to hybridized plants in botany. Atkinson and Gray [3] presented a table that highlights the most important conceptual parallels which can be drawn between these evolutionary phenomena. In particular, the latter study compares the process of social selection in linguistics to natural selection of species, borrowing of words across languages to horizontal transfer of genes, creole languages to plant hybrids, ancient texts to fossils, and cognates to homologies. There are also a few differences between these processes [4]. For instance, the biological alphabet (e.g., DNA) is universal, whereas the set of sounds used to form words is specific to each language. Moreover, the sequence data are usually much longer in molecular biology than in linguistics, and the selection of a perfect list of basic meanings suitable for the application of phylogenetic methods in the context of language evolution remains a challenging task. Nevertheless, the similarities and parallels between the two disciplines make it possible for researchers to use several well-developed computational biology methods for studying the evolution of species, and in particular reticulate evolution, in the field of linguistics. Obviously, it's not possible to apply these computational biology methods directly, without an appropriate adaptation, which is critical in interdisciplinary research. Thus, the existing phylogenetic algorithms should be modified and workflows adapted in order to obtain meaningful linguistic results and interpretations.

Two nucleotide sequences observed in two distinct species are said to be homologous if they have evolved from a common ancestral sequence [5]. Similarly, in linguistics, a group of cognates is a group of word forms in different languages that have been inherited from a common ancestral word form [6]. The main difference between these concepts is that the concept of homology includes the possibility of lateral transfers, whereas the concept of cognacy excludes all potential processes of borrowing. Cognates and phylogenetic trees play a fundamental role when studying the evolution of natural languages using phylogenetic methods [7]. For instance, a phylogenetic tree representing the main traits of lexical evolution is equivalent to a species phylogeny depicting the key speciation events [3, 7, 8]. Several linguistic studies used phylogenetic methods to better understand the evolution of Indo-European (IE) languages [7–11]. Discovering the origin and main evolutionary trends characterizing the IE language family is one of the most recalcitrant intellectual challenges in historical linguistics [7, 12]. Two opposing theories, Kurgan and Anatolian, concerning early Indo-European origins are generally considered [7]. The Kurgan theory [13, 14] postulates that IE languages originate from the Kurgan culture dated around 3000 to 4000 BC, whereas the Anatolian theory [15] dates the origin of IE languages around 7000 BC. For example, the works of Gray and Atkinson [7] and Bouckaert et al. [9], which focus on inferring and dating the divergence times of the contemporary and extinct IE languages using Bayesian phylogenetic methods, support the Anatolian theory of IE origin.

Phylogenetic tree model widely considered in linguistics assumes that the frequency of lateral word exchanges across languages has been relatively low. For example, Gray and Atkinson [7] and Bouckaert et al. [9] removed known loanwords from the basic vocabulary data before inferring their IE language trees. Obviously, linguistic phenomena such as word borrowing [10] and birth and evolution of hybrid languages [16], resulting from languages in contact, cannot be adequately represented by a tree model. For instance, a study of 80,000 words of the old Shorter Oxford Dictionary points out that English, which is a Germanic language, has borrowed 56.5 % of its total lexicon from Old French (Langue d’oïl) and Latin, 5.3 % from Greek, 13.2 % from other languages, and has inherited only 25 % of its current lexicon from its direct ancestor, Old Germanic [17, 18]. In this work, we analyzed basic vocabulary data from a 200-meaning Swadesh list [19]. While the use of this list may lead to a certain decrease in the number of loanwords [20], it remains helpful for detecting the most important word borrowing trends [21]. For example, the traditional 200-meaning English Swadesh list includes 33 confirmed loanwords (16.5 %) [22] and 10 additional “irregular phylogenetic patterns” which might be suggestive of unrecognized borrowings [21]. Moreover, in a recent revision of the Albanian Swadesh list 31.8 % of its entries were identified as probable borrowings [23].

Word borrowing can be viewed as one of the main development mechanisms leading to the emergence of hybrid (i.e., mixed or contact) languages. There exists a variety of hybrid languages, including pidgins, creoles, and lexical hybrids [24]. In a pidgin, the lexand minimum hybridization scoreicon usually comes from one parent language and the syntax comes from another one. A creole language, which arises from a pidgin, is a stable natural language spoken as a mother tongue. There are however many other types of lexical and grammatical transmission that produce a variety of linguistic outcomes. For example, Michif, which is the language of the Métis people of Canada and the United States, combines noun phrase phonology, lexicon, syntax and morphology from Métis French and verb phrase phonology, lexicon, syntax and morphology from Cree. As our analysis is based on lexical data only, here we address the problem of detection of lexical hybrids and word borrowing events.

Clearly, phylogenetic networks, and not phylogenetic trees, should be used to represent hybrid languages and word borrowing events. In fact, some drawbacks of the tree model in historical linguistic were already pointed out by Schmidt [25] in 1872. Nakhleh, Ringe and Warnow [26] were among the first to use directed phylogenetic networks to identify lexical contacts among 24 IE languages. These contacts have been represented by bidirectional reticulations, but the donor languages were not clearly distinguished from the recipient languages in the presented “perfect linguistic networks”. The study of Nakhleh and colleagues was restricted to the earliest attested languages of 12 subgroups of the IE family. Some other works that address the topic of modeling reticulate evolution in linguistics rely on the use of split graphs [3, 27, 28], minimal lateral networks (MLN) [10, 11, 21], and horizontal word transfer networks (HWTN) [29]. While the MLN and HWTN methods can be applied to detect word borrowing events, split graphs can be used to identify hybrid-like features of certain natural languages. For example, the split graph topology obtained for nine Germanic languages [27] allows one to identify Sranan, a language spoken in Suriname, as a hybrid of English and Dutch. However, split graphs were not specifically designed to detect and explicitly represent network relationships among languages. For instance, they cannot be used to identify explicitly the hybrid language, its parent languages and the corresponding hybridization/reticulation degree (i.e., percentage of lexical material transferred from each of the parent languages). Split graphs cannot be used to quantify the frequency of word borrowing events either. Furthermore, Wichmann and colleagues [30] proposed to infer reticulations based on distances retrieved from the Levenshtein metric [31] scores. Wang and Minett [32] used maximum parsimony to detect language contacts. The test, they designed, is based on the distribution of lexical similarities between languages. Köllner and Deller [33] proposed an ancestral state reconstruction method, which is specific to linguistics. The latter authors used the dissimilarities between a given node and its immediate ancestor in the tree in order to identify potential word borrowing events. In all these methods, the exact source and destination of the detected word borrowings cannot be identified explicitly. Only a few methods offer the advantage of finding the direction of reticulation events in linguistics. Mention here the work of Van der Ark et al. [34], who used the Levenshtein distance [31] to identify the source and the destination of word borrowing events, and that of Delz [35], who applied the horizontal gene transfer algorithm [36] from the T-Rex web server [37, 38] to detect loanwords and the corresponding word borrowings.

In this study, we adapt a recently developed computational biology method [39], which was originally designed to detect hybrid species, their parents and the corresponding hybridization degrees, to identify explicitly hybrid languages (i.e., lexical hybrids in this study) and word borrowing events. One of the main advantages of our method over the MLN [10, 11, 21] and perfect networks [26] approaches is that it allows for determining the direction of reticulation events (e.g., word borrowing events) in addition to the quantification of influence of each of the donor languages on the corresponding recipient languages. For a more complete description of the benefits and shortcomings of the MLN approach, the reader is referred to [40–42]. We compare our explicit hybridization networks to the corresponding split graphs [43, 44] and galled networks [45]. Finally, we present some historical evidence that supports the results of our analysis.

## Methods

### Data description

Several important studies dedicated to the classification of IE languages [7, 8, 10, 29] have examined the data from the 84 IE language database organized by Dyen and colleagues [46]. The Dyen database contains word forms for the meanings of the 200-meaning Swadesh list [19]. This list is one of a few lists of fundamental meanings collected by M. Swadesh in the 1940s and 50s. It is often used in lexicostatistics, which focuses on quantitative evaluation of lexical cognates, and in glottochronology, which focuses on dating divergence times of natural languages. Swadesh lists have been used by linguists to test the level of chronological separation of languages by comparing words, as they contain universal stable items with low levels of borrowing [7, 8]. However, it has been noticed that even though the use of Swadesh lists may decrease the level of borrowings to a certain degree, it cannot exclude all of them [21]. For each of the 200 basic meanings of the Swadesh list, the Dyen database contains their word forms in 84 IE languages. These word forms have been regrouped in cognate sets [46]. Two word forms were identified as cognate if they share an uninterrupted evolutionary history characterized by the presence of a common ancestral form. The word forms resulting from word borrowing (e.g., English word fruit which was borrowed from Old French) and those related by accidental similarity (e.g., the word form bad exists in both English and Farsi, but this is rather considered as an accidental similarity by linguists) were placed in a separate class. When it was difficult to differentiate between cognates and word forms resulting from borrowing or accidental similarities, the corresponding word forms, albeit not numerous, were categorized as doubtful cognates. For instance, this database was used by Gray and Atkinson [7] and Atkinson and Gray [47] to infer evolutionary trees of IE languages. In order to reconstruct our hybridization networks, we also considered some additional linguistic resources (Douglas Harper’s Online Etymology Dictionary [48], the IE Lexical Cognacy Database (IELex) [49] and the IE etymological dictionaries collection [50]), which include relevant etymological information regarding loanwords and accidental similarities. Using these resources, we modified some of the original cognate sets created by Dyen et al. [46]. Precisely, the loanwords, put aside by Dyen and colleagues, were added to the corresponding cognate sets (i.e., cognate sets containing the donor forms for these loanwords). In some rare cases, the original cognate sets including doubtful cognates were either merged or eliminated. In total, our modified database included 1315 cognate sets. It is available at: http://www.trex.uqam.ca/biolinguistics.

### Reconstruction of explicit linguistic hybridization networks

**Fig. 1:**
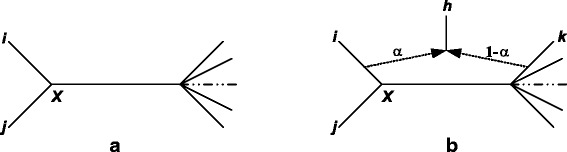
**a** Configuration in which languages *i* and *j* are selected as neighbors by the NJ algorithm, and **b** configuration in which language *h* is identified as a recipient of lexical material from languages *i* and *k* by our algorithm for inferring explicit hybridization networks (here, the parameters *α* and 1-*α* represent the hybridization (i.e., reticulation) degree of donor languages *i* and *k*, respectively)

**Fig. 2:**
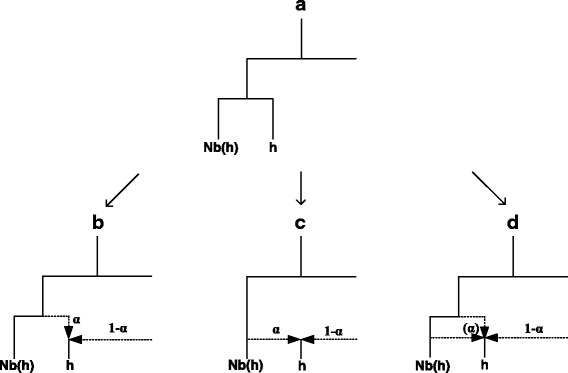
This figure illustrates three possible network configurations (**b–d**), when our algorithm detects a hybrid, *h*, which is neighbour of one of its parents, *Nb*(*h*), in the phylogenetic tree (**a**), e.g., in the IE language phylogeny inferred by Gray and Atkinson (see Fig. 1 in [6]). In configuration **b**, language *h* receives the proportion, *α*, of its lexicon from its closest ancestor in the tree via direct inheritance and the remaining part of its lexicon, (1-*α*), from a distant parent via word borrowing (e.g., see the case of Penn Dutch in Figs. 4 and 5b). In configuration **c**, language *h* is a lexical hybrid of *Nb*(*h*) and a distant parent (e.g., see the case of Sranan in Figs. 4 and 5b). In configuration **d**, language *h* receives the proportion *α* (indicated, in this case, in parentheses) of its lexicon from both its closest ancestor via direct inheritance and from its neighbour *Nb*(*h*) via word borrowing, and the remaining part, (1-*α*), of its lexicon from a distant parent via word borrowing (e.g., see the case of Old Armenian in Fig. 4)

In [39], we presented a new algorithm for inferring explicit hybridization networks from distance data. This algorithm takes as input a matrix of evolutionary distances between species of size (*n*x*n*) and the three following user-defined parameters: minimum and maximum levels of hybridization (the value of these parameters varies between 0 and 1), and minimum hybridization score. The output of this algorithm, based on a famous neighbor-joining (NJ) principle [51], is either a traditional phylogenetic tree with *n* leaves or a hybridization network with *n* terminal nodes. It is worth noting that NJ remains by far the most popular distance-based method in phylogenetics, even though in linguistics Bayesian framework is also frequently used [7]. NJ is specifically well suited for the inference of large phylogenies. It takes as input a distance matrix **D** = {*d*(*i*, *j*)}_1 ≤ *i*,*j* ≤ *n*_ defined on a set of *n* species (i.e., taxa or languages) and gives as output a phylogenetic tree representing their evolutionary history. NJ starts with a star tree including *n* leaves, one internal node and *n* branches. This tree is progressively transformed into an unrooted binary phylogeny with *n* leaves and 2*n-*3 branches. The *p-*th step of NJ consists of selecting and connecting the two most appropriate neighbors among (*n* − *p* + 1) candidates. For all of the (*n* − *p* + 1)(*n* − *p*)/2 tree configurations equivalent to that shown in Fig. 1a, the branch lengths are calculated according to the least-squares criterion. The configuration that minimizes the sum of all branch lengths of the tree is then selected and the two nodes *i* and *j*, which are neighbors in this configuration, are connected as shown in Fig. 1a. The nodes *i* and *j* are then replaced by the node *X* (their direct common ancestor; Fig. 1b) and the distance matrix **D** is updated by computing the new distances *d*(*X*, *k*), from *X* to each remaining leaf *k* of the tree, by means of the following formula $d\left(X,k\right)=\frac{1}{2}\left(d\left(i,k\right)+d\left(j,k\right)\right)$. We used the NJ criterion [51] to infer explicit hybridization networks between species [39] and adapted it here to the identification of hybrids and word borrowings among natural languages. Note that in our networks both terminal and ancestral branches can be involved in hybridization. Obviously, the two parent branches (i.e., languages or groups of languages) are not necessarily neighbors. Each hybrid language (or recipient of lexical material) is explicitly identified along with its parent languages (or donors) and the degree of hybridization (or reticulation) corresponding to each of them. In the case of word borrowing, this degree of hybridization represents the proportion of the relative influence of each of the two donors on the recipient (Fig. 2c). As we will see later, it can also take into account the direct inheritance part of the recipient’s lexicon (Fig. 2b, d).

Here we present some important computational details of our algorithm. We use the following formula to determine the level of hybridization, *α*_*i*,*j*_^*h*^, for each possible triplet of languages, (*h, i, j*), assuming that *h* is a hybrid of *i* and *j*:

1 $$ {\alpha}_{i,j}^h=\frac{{\displaystyle {\sum}_{k\ne i,j,h}{X}_k\left({Y}_k-{S}_h+{S}_j\right)}}{{\displaystyle {\sum}_{k\ne i,j,h}{X}_k{X}_k}}, $$

where ${S}_l=\frac{{\displaystyle {\sum}_{k\ne i,j,h}d\left(k,l\right)}}{n-3}$ (for *l* = *h, l = i* or *l* = *j*), *Y*_*k*_ = *d*(*k*, *h*) − *d*(*k*, *j*) and *X*_*k*_ = *S*_*j*_ − *S*_*i*_ + *d*(*k*, *i*) − *d*(*k*, *j*). Formula 1 was obtained by minimizing the following least-squares function of *α* (its minimum is attained with *α* = *α*_*i*,*j*_^*h*^):

2 $$ L{S}_{i,j}^h={{\displaystyle {\sum}_{k\ne i,j,h}\left({Y}_k-{S}_h+{S}_j-\alpha {X}_k\right)}}^2 $$

the hybridization (reticulation) score, *Sc*_*i*,*j*_^*h*^, is defined as follows for all triplets of languages (*h, i, j*):

3 $$ S{c}_{i,j}^h=\underset{k\ne i,j,h}{Min}\left\{d\left(i,j\right)+d\left(k,h\right)-d\left(i,h\right)-d\left(k,j\right);\ d\left(i,j\right)+d\left(k,h\right)-d\left(j,h\right)-d\left(k,i\right)\right\} $$

Formula 3 is related to the four point condition, which is satisfied in an additive tree (i.e., phylogenetic tree), but not in a phylogenetic network. We restrict the search of hybrids to the triplets of languages satisfying the following constraints: *Sc*_*i*,*j*_^*h*^ ≥ *MIN*_*Sc*_ and *α*_*MIN*_ ≤ *α*_*i*,*j*_^*h*^ ≤ *α*_*MAX*_, where the parameters 0 < *α*_*MIN*_ < *α*_*MAX*_ < 1 and *MIN*_*Sc*_ are selected by the program’s user depending on the desired number of hybridization events (see [39] for more details about parameter selection).

Our network reconstruction algorithm can be defined as follows. First, we determine the languages *i* and *j* that should be connected at the current step by the traditional NJ algorithm. Prior to connecting *i* and *j*, we identify the language *h* that is the best candidate for being a hybrid of either *i* or *j* (Parent 1 of *h*) and any other remaining language *k* (Parent 2 of *h*; see Fig. 1b). We search for the language *h*_0_ that maximizes the absolute value of the following function:

4 $$ {\varDelta}_{i,j}^h={\displaystyle {\sum}_{k\ne i,j}\left(d\left(j,h\right)+d\left(i,k\right)-d\left(j,k\right)-d\left(i,h\right)\right).} $$

Note that *Δ*_*i*,*j*_^*h*^ equals 0 if *i* and *j* are true neighbors in an additive tree.

Then, we select the triplet (*h*_0_*, i*_0_*, k*)*,* here *i*_0_ *= i* or *i*_0_ *= j*, that provides the minimum of the least-squares function *LS*_*i*,*j*_^*h*^ and satisfies the above-mentioned constraints. If *LS*_*i*,*j*_^*h*^ < (*Δ*_*i*,*j*_^*h*^)^2^, we consider that *h*_0_ is a hybrid of *i*_0_ and *k*, and remove from the distance matrix the row and the column corresponding to *h*_0_. Otherwise, we connect the languages *i* and *j* as in the conventional NJ algorithm [51]. The time complexity of our network building algorithm is *O*(*n*^3^), which is equivalent to the time complexity of NJ.

It’s important to mention that hybrid languages identified by our algorithm should not be always interpreted as real lexical hybrids or real mixed languages. In some cases, the detected parent-hybrid relationship may also represent the processes of word borrowing or even inheritance from the closest ancestor in the tree (see Fig. 2). This figure illustrates three possible network configurations which reflect the case where our algorithm detects a hybrid, *h*, which is a direct neighbour, or a very close neighbour, of one of its parents, *Nb*(*h*), in the phylogenetic tree (Fig. 2a). This tree is assumed to be inferred by a traditional tree reconstruction algorithm (e.g., NJ). For instance, language *h* may receive the proportion, *α*, of its lexicon either from its closest ancestor in the tree via direct inheritance (Fig. 2b), or from its neighbour *Nb*(*h*) in the tree as its lexical hybrid (Fig. 2c), or from both its closest ancestor via inheritance and from its neighbour *Nb*(*h*) via word borrowing (Fig. 2d; *α* is indicated in parentheses in this case).

**Fig. 3:**
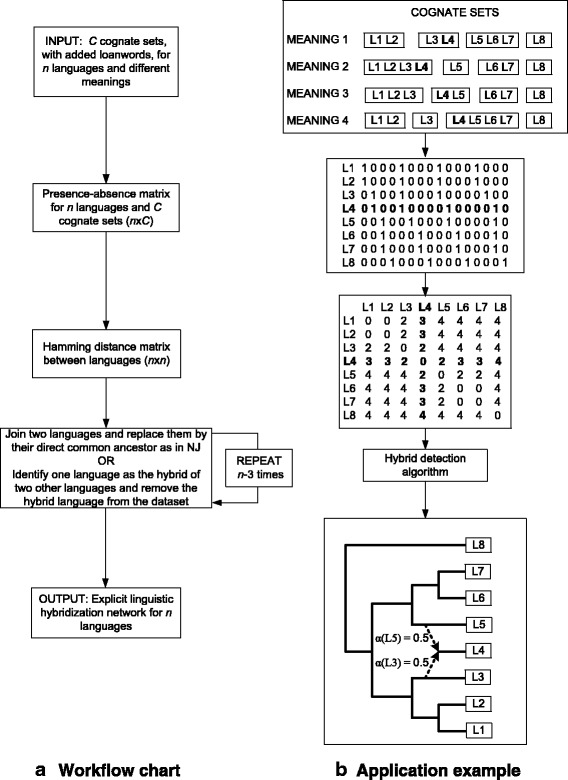
**a** Workflow chart of the new method for inferring explicit hybridization networks, and **b** an example of its application to a dataset consisting of 8 languages (including the hybrid language L4), 4 meanings and 16 cognate sets

We tested several strategies of computing the distance matrix **D** between the 84 IE languages considered in our study. As Dyen’s database [46] does not contain any word form from the Hittite and Tocharian languages, these ancient languages were discarded from our analysis. The first strategy, which provided the most plausible experimental results, used a binary presence-absence matrix of languages over the established cognate sets (1315 cognate sets in total). It is worth noting that our binary encoding concerned language presence-absence data only (e.g., as in [7]). The presence-absence matrix **D** had 84 rows and 1315 columns. The element (*i, j*) of this matrix was equal to 1 if a word form of language *i* was present in cognate set *j*, otherwise it was equal to 0. In total, 19.69 % of the data were missing in our database. Missing data were mostly due to the presence of the corresponding word forms in the special “non-cognate” class of the Dyen’s database; such word forms that were neither cognate with any other word form of the given meaning nor related to any word form by the way of borrowing were excluded from our database. The distance between any pair of languages was then calculated as the Hamming distance between the rows corresponding to these languages in the presence-absence matrix (i.e., it was equal to the number of cognate sets that contained word forms of only one of these languages). Two data encoding strategies were tested. The first, when the missing data were encoded by 0’s, and the second, when the Hamming distance between two languages was normalized by the number of meanings for which the corresponding word forms existed in both languages. As these two strategies provided very similar hybridization networks, only the results of the first strategy will be presented. The workflow chart of our method is presented in Fig. 3a, and a simple example of its application is shown in Fig. 3b. Here we consider a dataset with 8 languages, L1, L2, …, L8, 4 meanings and 16 cognate sets (i.e., 4 cognate sets for each meaning). According to the language content in these 16 cognate sets, language L4 can be seen as a hybrid of languages L3 and L5. Language L8 is used as an outgroup. In the first (respectively, second) step of our algorithm, languages L1 and L2 (respectively, L6 and L7) are joined, following the NJ principle. Then, before joining (L1, L2) and L3, language L4 is identified as a hybrid of L3 and L5 with the degree of hybridization, *α*, equal to 0.5 for both of its parents. Language L4 is then removed from the dataset, and the remaining steps of the algorithm correspond to the steps of traditional NJ. The obtained explicit hybridization network is presented in Fig. 3b.

We also conducted the analysis using the Levenshtein distance [31] between words of the same meaning but did not obtain convincing results using such an approach. This should be due to the fact that this distance tends to reflect chance similarity when the compared word forms are not cognate [52]. The Levenshtein distance will be further used for inferring galled networks from word trees, but its application will be restricted to word forms belonging to the same cognate set.

**Fig. 4:**
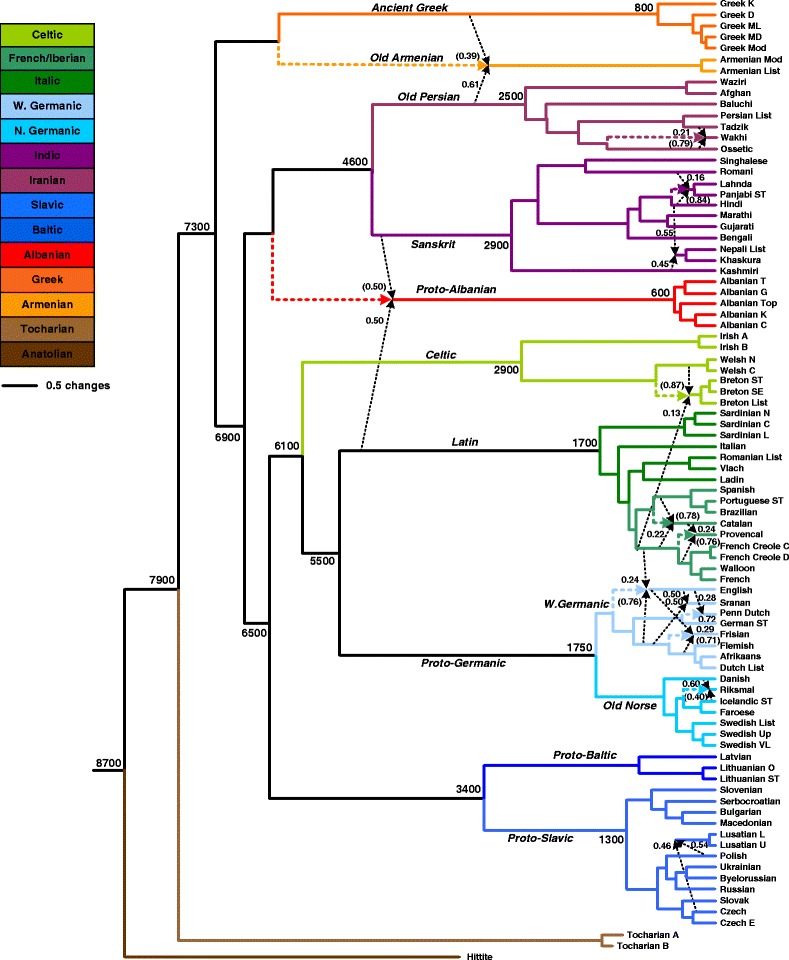
Explicit hybridization network given by our algorithm for the group of 84 IE languages originally considered by Dyen et al. [32]. The tree topology in this network corresponds to the IE language phylogeny inferred by Gray and Atkinson (see Fig. 1 in [6]). Language groups are indicated on the left. The numbers at the arrows are the reticulation degrees corresponding to each of the donor languages and the numbers at the internal tree nodes are their age estimates

**Fig. 5:**
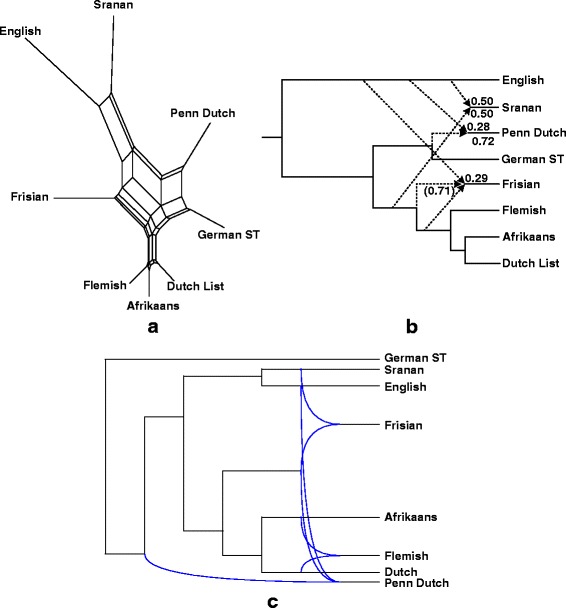
Split graph (**a**), explicit hybridization network (**b**) and galled network (**c**), obtained for 8 languages of the West-Germanic group

**Fig. 6:**
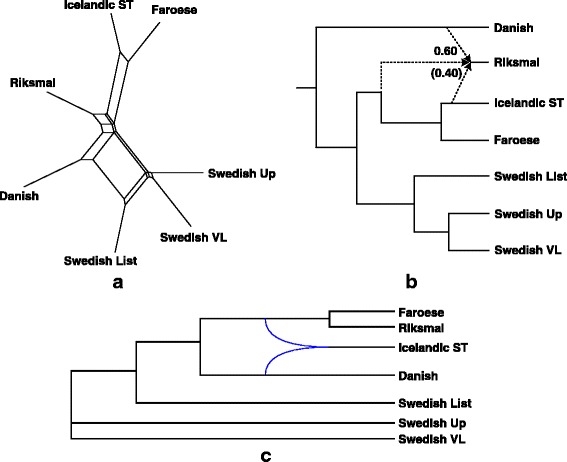
Split graph (**a**), explicit hybridization network (**b**) and galled network (**c**), obtained for 7 languages of the North-Germanic group

**Fig. 7:**
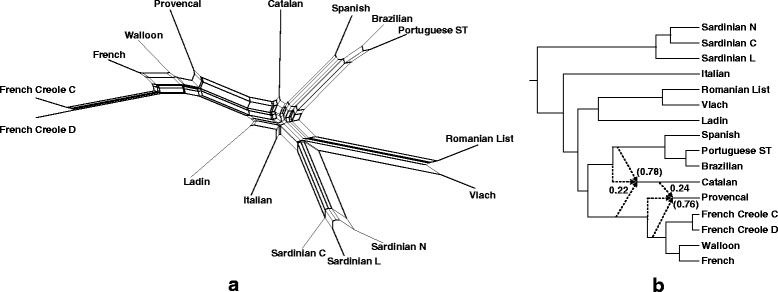
Split graph (**a**) and explicit hybridization network (**b**), obtained for 16 languages of the Latin group

We applied our hybridization network inferring algorithm to the entire Hamming distance matrix of 84 IE languages, denoted here by **D**_84_, as well as to its submatrices corresponding to each of the 11 considered IE language groups. In particular, some plausible lexical hybrids and word borrowing donors and recipients were found when the submatrices of the five following language groups were analyzed: Germanic, Latin (including the Italic and French/Iberian groups), Slavic, Sanskrit and Persian (see Figs. 4, 5b, 6b and 7b for the detailed results). Furthermore, the analysis of two submatrices corresponding to the union of the West Germanic and French/Iberian groups and the union of the Celtic and French/Iberian groups also provided very relevant results. We did not find additional reticulations within the other IE groups. We needed a distance matrix of size greater than four to be able to apply our algorithm. It is worth noting that the recovery of hybrid languages and word borrowing events seemed to be more complicated within smaller linguistic groups (i.e., groups with five or six taxa here).

The input parameters of our algorithm, *MIN*_*Sc*_, *α*_*MIN*_ and *α*_*MAX*_, were chosen according to the size of the considered distance matrix (see [39] for a detailed discussion on the parameter selection). For smaller distance matrices corresponding to particular language groups, the following set of input parameters: (*MIN*_*Sc*_ = 0, *α*_*MIN*_ = 0.1 and *α*_*MAX*_ = 0.9) was used. To avoid an excessive number of false positives, more restrictive parameters: (*MIN*_*Sc*_ = 0.1, *α*_*MIN*_ = 0.25 and *α*_*MAX*_ = 0.75) were used for the entire distance matrix **D**_84_. For the representation of our hybridization networks (Figs. 4, 5b, 6b and 7b), we used the backbone IE phylogenetic tree inferred by Gray and Atkinson (Fig. 1 in [6]), mapping into it the detected lexical hybrids and word borrowing events with their respective reticulation degrees. Our program for inferring explicit hybridization networks is available at:

www.info2.uqam.ca/~makarenkov_v/makarenv/hybrids_detection.zip. The data used in our study can be found at: www.trex.uqam.ca/biolinguistics/Biolinguistic_networks_data.zip.

### Reconstruction of split graph-based linguistic networks

**Fig. 8:**
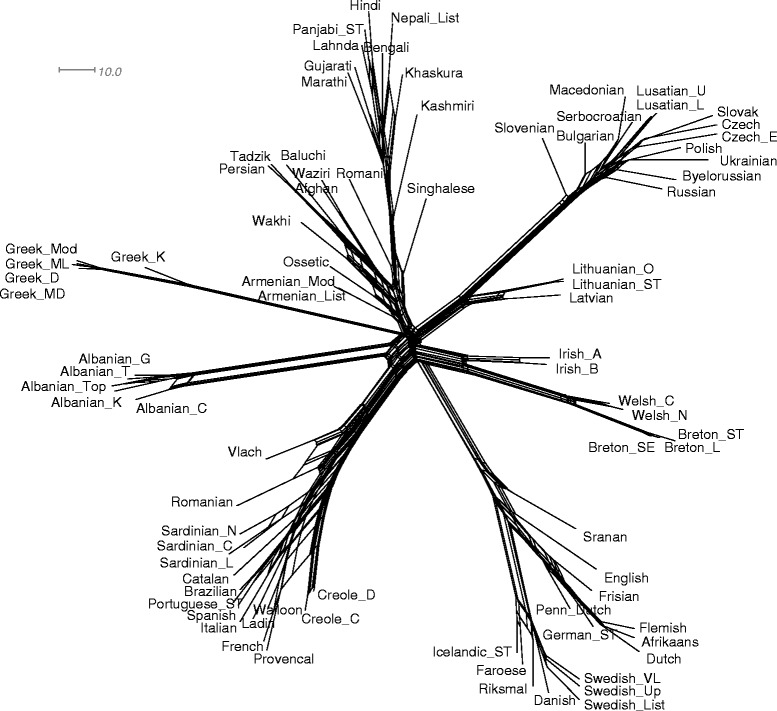
Split graph obtained for the entire set of 84 IE languages

The split decomposition method introduced by Bandelt and Dress [43] decomposes the given distance matrix into simple components based on weighted splits (i.e., bipartitions of taxa, species or languages). These splits can then be represented using a split graph, a particular type of phylogenetic network that simultaneously represents both clusters in the data and evolutionary distances between taxa. The Neighbor-Net method introduced by Bryant and Moulton [44] and implemented in the SplitsTree program [53] works in a similar way, but constructs phylogenetic networks that are much more resolved than those given by split decomposition. Split graphs have been widely used in phylogenetic studies to depict phylogenetic relationships between species, but several works have also considered their applications in historical linguistics [3, 27]. We used SplitsTree [53] to infer the split graphs corresponding to the West Germanic, North Germanic and Latin groups of IE languages, with the same submatrices of **D**_84_ as mentioned above. A total of 22 (respectively, 16 and 51) splits were identified for the West Germanic (respectively, North Germanic and Latin) language groups. These split graphs will be compared to our hybridization networks and galled phylogenetic networks inferred for the same groups of languages (Figs. 5, 6 and 7). Figure 8 shows the split graph, with 371 splits, obtained for the entire set of 84 IE languages examined in our study.

### Reconstruction of galled linguistic networks from word trees

Several methods have been developed for inferring consensus phylogenetic networks from contradictory sets of two or more phylogenetic trees. They include, among others, cluster networks [54], galled networks [45] and level-*k* networks [55]. A cluster network is a rooted phylogenetic network obtained from a given set of clusters (i.e., set of bipartitions). In such a network, every branch represents exactly one input cluster. A galled network is a rooted phylogenetic network in which each reticulation has a tree cycle. A tree cycle is an undirected cycle consisting of two disjoint tree paths between a tree node and a reticulation node. A level-*k* network is a rooted phylogenetic network, such that the maximum number of reticulations contained in a biconnected component equals *k*. A given set of clusters can always be represented by a galled network, but not necessarily by a level-*k* network [55]. These three methods have been implemented in the Dendroscope software [56]. We conducted our analyses with all of them but present here only the results of the galled network method which provided the “most interpretable” linguistic networks (i.e., networks in which the obtained reticulations correlate the best with known contacts between natural languages). Since for running this method we needed a set of phylogenetic trees, we reconstructed word phylogenies for each of the 200 meanings of the Swadesh list. We used the normalised Levenshtein metric [31], denoted here by *d*_*L*_, to calculate the distances between the cognate word forms of the same meaning; the distance between any two non cognate word forms was set to 1 (see below for more details). The Levenshtein distance between two words is defined as the minimum number of editing operations, consisting of insertions, deletions and substitution of a single letter, necessary to transform one word into the other. This distance was normalized by the maximum length of two words. The Levenshtein distance has been criticized as a poor distance for building language trees because of its reflection of chance similarity when the compared words are not cognate [52]. Our comparative study presented below suggests that this distance can be used for building word trees from cognate data.

Several recent linguistic studies argued that accurate comparisons between words should also incorporate likely changes to pronunciation and phonological system [52, 57]. Thus, we decided to compare, in terms of reconstruction word trees and word borrowing events, the normalized Levenshtein distance with the SCA (Sound-Class-based phonetic Alignment) distance recently introduced by List [58]. While the Levenshtein distance applies to orthographic data, the SCA distance is based on the comparison of phonological forms. Note that phonological forms are still not available for many word forms of Dyen’s database. Thus, among 42 cognate sets that were found to be suggestive of borrowing into English according to the modified MLN approach [21], we selected the 28 cognate sets (Table 1) for which at least four cognates with available phonological forms were present in the IELex database [49]. Trees with less than four leaves have identical topologies and thus cannot be used to recognize word borrowings [29]. It is important to note that the MLN approach is an automatic approach based on tree topology and the 42 suggestive cases of borrowing recovered by MLN, which include 33 English loanwords identified by Donohue et al. [22], cannot be considered as crystal-clear borrowings. They may comprise some false positives, which can be due to parallel semantic development [21], for example.

**Table 1:**
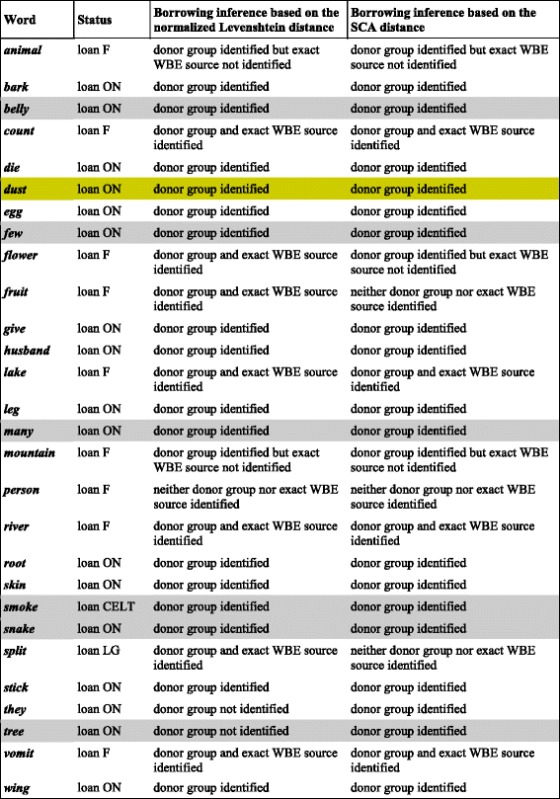
This table reports the results provided by the word borrowing event detection algorithm [29] applied to the normalized Levenshtein [31] and SCA [58] distance matrices

Cases of confirmed borrowing, according to Donohue et al. [22] are not colored; cases of possible borrowing, which could have been unrecognized according to List et al. [21], are shaded in gray (note that List and colleagues point out that the grayed words can also represent cases of parallel semantic development [21]); an additional suggestive case of borrowing (i.e., dust) is in yellow. When the Old North (ON) group was assumed to be the word donor, the exact source of borrowing in the ON group was not considered since it remains unknown in most of these cases. The Neighbor-Joining algorithm [51] was carried out to reconstruct phylogenies from distance matrices. Here, we considered all of the confirmed or suggested loanwords of the basic English vocabulary associated with the 200-word Swadesh list. The loanword information was taken from the studies of Donohue et al. ([22]; Supplementary Material) and List et al. ([21]; modified MLN approach). To the 33 English loanwords identified by Donohue et al. [22] and 8 possible additional English borrowings suggested by List et al. ([21]; Supplementary Material II; Table C), we added the word *dust*, which might have been borrowed by English from Old North according to the results given by our algorithm for detecting word borrowing events [29]. This English word was also identified as belonging to an irregular pattern, which may be suggestive of borrowing, by the modified MLN approach [21]. To compare the results yielded by the normalized Levenshtein distance [31] and the SCA phonetic distance [58], we examined 28 of the 42 above-mentioned cases of borrowing into English, i.e., all the cases for which at least four cognate phonological forms were available in the IELex database (see http://ielex.mpi.nl [49] for the cognate sets that included the considered English word forms). Thus, 28 of 42 suggested English borrowings (see the table) were examined along with their donor word form and all of the donor’s cognates that had phonological entries in IELex (the data were taken in September 2015). The detailed results have been included in the archive file Biolinguistic_networks_data.zip available at: http://www.trex.uqam.ca/biolinguistics. This data archive comprises word tree representations, lexeme and phonological distance matrices, and the Perl script for computing the normalised Levenshtein distance

*Abbreviations*: *F* French, *ON* Old North, *CELT* Celtic group, *WBE* word borrowing event

We applied our algorithm for inferring word borrowing events [29] to the word trees obtained with the normalized Levenshtein and the SCA distances (the inferred word tree topologies are available in Additional file 1). The results provided by using these distances can be considered as equivalent. The normalized Levenshtein distance allowed us to identify 23 of 28 suggested borrowings, while the SCA approach was able to detect 22 of them. For instance, the SCA-based algorithm was unable to recover the correct borrowings into English for the words *flower*, *fruit* and *split* (Table 1). The results of this analysis as well as the fact that orthographic cognate data are much more complete than phonological ones are the main reasons that justify the use of the normalized Levenshtein distance for inferring word trees. It is worth noting that one of the most significant differences between language history and biological evolution is that in the case of natural languages our alphabet systems change, while biological sequences change via mutation. Thus, methods using the Levenshtein distance as well as the more historically-oriented SCA distance may have shortcomings, since both of these distances are based on the idea that similarities and differences are due to mutations. For example, the distance between French *tête* and Latin *testa* should be 0 in linguistic terms, since the sound change was completely regular. Moreover, some words may contain cognate material, but only in parts. For example, the French word *soleil* is different from Italian *sole*, since it stems from a suffixed form of Latin *sol*, namely Latin *soliculus*. This case cannot be handled successfully by the Levenshtein and SCA distances, and the use of any of them will lead to the addition of noise to the distance matrix.

Borrowings can be seen as mutations in some parts, since they are not produced by regular sound change. Thus, methods based on sequence similarity, like those using the Levenshtein distance, may have advantages in identifying borrowings over methods that seek to ignore regular dissimilarities between words, like those using the SCA distance. Furthermore, the presented method could be modified to account for language-specific distances, which could be measured by other algorithms, as for example, the LexStat algorithm by List [42] or the algorithm proposed by Steiner et al. [59].

**Fig. 9:**
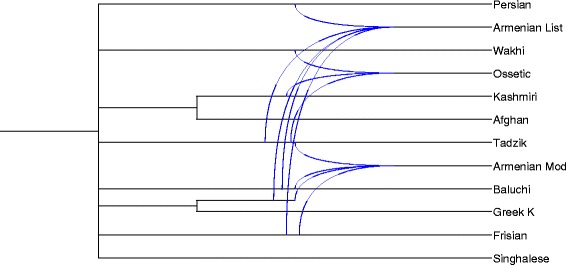
Partial galled network obtained for 12 IE languages. This is a maximum sub-network that includes reticulations of the complete galled network built for the entire set of 84 IE languages

For each considered meaning *m* of the 200-meaning Swadesh list, we denoted by *L*_*m*_ the set of languages for which we had at least one word form of *m* in our database, and by *C*_*m*_ the collection of cognate sets available for the meaning *m*. Let *n*_*m*_ be the cardinality of *L*_*m*_. Note that for most of the meanings, the value of *n*_*m*_ was lower than 84 since our database, as well as its original version created by Dyen, had some missing word forms for almost all the meanings. Mention that in some, rather rare, cases multiple word forms of the same language existed for a given meaning *m*. For each meaning *m*, a distance matrix **D**_*m*_ of size *n*_*m*_ was computed by applying the following formula to each pair of languages, *l*_1_ and *l*_2_, in *L*_*m*_:

5 $$ {d}_m\left({l}_1,{l}_2\right)\kern0.5em =\frac{{\displaystyle \sum_{c\in {C}_m}{d}_c\left({l}_1,{l}_2\right)}}{n_{l_1,{l}_2}}, $$

where *d*_*c*_(*l*_1_*,l*_2_) was equal to 0 if neither word forms of *l*_1_ nor those of *l*_2_ were present in *c*; *d*_*c*_(*l*_1_*,l*_2_) was equal to 1 if word forms of either only *l*_1_ or only *l*_2_ were present in *c*; and, it was equal to the minimum value of *d*_*L*_(*i,j*), over all cognates *i* representing *l*_1_ and all cognates *j* representing *l*_2_ in *c* if word forms of both *l*_1_ and *l*_2_ were present in *c*. The integer ${n}_{l_1,{l}_2}$ was the number of cognate sets of the meaning *m* that included at least one word form of either *l*_1_ or *l*_2_. Thus, we obtained 200 distance matrices **D**_*m*_ of different sizes. For each such a matrix, we then inferred the corresponding unrooted word phylogeny *T*_*m*_ using the NJ algorithm [51]. The obtained word phylogenies were given as input to the galled network algorithm [45]. Since these word trees did not contain the same sets of languages (i.e., tree leaves), we used the Z-closure method, available in Dendroscope 3 [56], to merge partial data [60]. Figures 5c, 6c and 9 present the most plausible networks provided by the galled network algorithm [45]. First, we inferred networks from the trees restricted to the languages of the West Germanic (Fig. 5c) and North Germanic (Fig. 6c) groups. The trees including at least four Germanic languages (West or North) were analyzed. Here we considered splits that were present in at least 30 % of the input trees. In the case of the West Germanic group, we examined 190 input trees with 207 input splits, 237 splits after Z-closure, and 76 remaining splits after the removal of partial splits. The consensus galled network obtained for the West Germanic group (Fig. 5c) contains 20 splits and 3 putative lexical recipients (i.e., *Frisian, Flemish* and *Pennsylvania Dutch*). In the case of the North Germanic group, we examined 188 input trees with 109 input splits, 112 splits after Z-closure, and 49 remaining splits after the removal of partial splits. The consensus galled network for the North Germanic group (Fig. 6c) contains 12 splits and 1 putative lexical recipient (i.e., *Icelandic ST*). We also inferred a galled network for a total of 84 IE languages. In this case, we considered only the splits that were present in at least 75 % of the input trees to avoid false positive reticulations. Figure 9 illustrates a sub-network of 12 IE languages that contains all the reticulations identified in the complete galled network of 84 languages. Here we considered 200 input trees with 6,176 input splits, 11,299 splits after Z-closure and 5,124 remaining splits after the removal of partial splits to obtain a consensus galled network with 101 splits and 3 putative recipient languages (i.e., *Armenian List*, *Armenian Mod* and *Ossetic*). The presented network correctly identifies the influence of the languages of the Iranian group and that of Ancient Greek on Armenian, but also includes false positive reticulations reflecting, for example, the influence of Frisian on Armenian.

## Results and Discussion

Hybrid languages emerge in a few generations as a new means of communication between two (or more) populations not sharing a common language. In many cases, e.g., when we found that Old Armenian is a lexical hybrid of Old Persian and Old Greek, we should interpret the results of our algorithm as the identification of the influence, e.g., cultural, political or military, which the two parent languages (i.e., the donors) had on their lexical hybrid (i.e., the recipient), at possibly different periods of time, and which could last over several centuries.

As known from evolutionary biology, the position of hybrid species in a phylogenetic tree or network is often uncertain [61]. Furthermore, some of the hybrids added to the data can influence the position of their parents when a phylogenetic tree or network is inferred. Often a hybrid is placed as a direct neighbor of one of its parents in a phylogenetic tree or network, and the parents’ location may change when this hybrid is removed from the data set. Thus, some of the results presented in this section were obtained after rerunning our algorithm on the distance matrices from which the detected lexical hybrids, identified at the first run of this algorithm, were removed.

Here we present the most important reticulation events characterizing the evolution of IE languages which were identified by the three competing algorithms for inferring split graphs, galled networks and our explicit hybridization networks, respectively. The related historical facts and justifications are also discussed. Since only lexical data were considered in our study, the presented phylogenetic networks represent interactions between languages which are mainly based on lexical borrowings. They do not account for other language interactions, such as contact-induced syntactic restructuring, for example.

### Network relationships within the Germanic group

We carried out our algorithm independently for the languages of the West Germanic group, the North Germanic group, and finally, the entire Germanic group. Four putative lexical hybrids were discovered in this analysis (Figs. 5b and 6b):

- *Pennsylvania Dutch* as a recipient of lexical material from *English* (by word borrowing) and *German* (by inheritance): Pennsylvania German or Pennsylvania Dutch (Penn Dutch) is a variant of German developed by the descendants of German, French (from Alsace and Lorraine) and Swiss emigrants to the East Coast of the United-States [62]. These migrants settled in the Unites-States in the 17th and 18th centuries. Pennsylvania Dutch borrowed many words from English, particularly in the 19th century.
- *Frisian* as a recipient of lexical material from *Old English* (by word borrowing) and the ancestor of *Flemish, Afrikaans* and *Dutch* (by word borrowing, but the inheritance from a close common ancestor is also possible here): The Frisian dialects are spoken in the northern parts of the Netherlands and Germany [63]. They are the closest living languages to English, after Scots. Due to the long lasting influence of Old Dutch (since the Middle Ages), Frisian is now more similar to Dutch than to English (see a greater reticulation degree obtained for Old Dutch than for Old English in Fig. 5b, i.e., 0.71 vs. 0.29).
- *Sranan* as a recipient of lexical material from *English* (by word borrowing) and *Old Dutch* (by word borrowing): Sranan is an English-based creole language spoken in Suriname [64]. After the invasion of Suriname by the Dutch in 1667, Sranan’s vocabulary was greatly influenced by Dutch. Sranan also borrowed some Portuguese and African words.
- *Riksmål* as a recipient of lexical material from *Danish* (by word borrowing) and *Icelandic* (by word borrowing, but the inheritance from a close common ancestor is also possible here): Historically, the North Germanic languages were divided into three main branches: East Scandinavian (Danish and Swedish), West Scandinavian (Icelandic, Faroese and Norwegian) and Old Gutnish [65]. Riksmål (or Bokmål) is now the most widely-used written standard of contemporary Norwegian. It was strongly influenced by Danish, because of the political domination of Denmark over Norway during several centuries. Nowadays, Riksmål is closer to Danish than to Icelandic and Faroese (see the corresponding reticulation degrees in Fig. 6b).

The following common features can be observed when comparing the explicit networks provided by our algorithm to those given by the split graphs (Figs. 5a and 6a) and galled networks (Figs. 5c and 6c) methods. In the case of the North Germanic group, the split graph (Fig. 6a) allows us to identify Riksmål as a potential lexical hybrid of Danish and the ancestor of Icelandic and Faroese. Very similar reticulations were found by our method (Fig. 6b). However, the split graph does not yield any quantitative estimation of the influence of donor languages on recipient languages. In the case of the West Germanic group, the identification of network relationships in the split graph is more sophisticated (Fig. 5a). For example, we could implicitly identify in this graph the same lexical recipients as in our explicit network, but we could also see German as a recipient of lexical material from (Flemish, Afrikaans and Dutch) and Pennsylvania Dutch, or the ancestor of (Flemish, Afrikaans and Dutch) as a lexical recipient of German and Frisian. The galled network method yielded more explicit linguistic networks than split graphs. However, the galled network obtained for the North Germanic languages (Fig. 6c) incorrectly identifies Icelandic as a recipient of lexical material from Danish and the ancestor of (Faroese and Riksmål). For the West Germanic group (Fig. 5c), the reconstructed galled network was able to depict two correct recipients languages: Frisian and Pennsylvania Dutch. Nevertheless, Flemish was wrongly identified as a recipient of lexical material from Dutch and Afrikaans, and Sranan was not detected as a lexical hybrid but rather as one of the donors of Frisian.

### Network relationships within the Latin group

Only two possible lexical hybrids were identified by our algorithm in the Latin group (including Italian and French**/**Iberian subgroups; Fig. 7b):

- *Catalan* as a recipient of lexical material from the ancestor of *Spanish, Portuguese* and *Brazilian* (by word borrowing, but the inheritance from a close common ancestor is also possible here) and from *Old French* (by word borrowing), and
- *Provençal* as a recipient of lexical material from *Catalan* (by word borrowing) and *Old French* (by word borrowing, but the inheritance from a close common ancestor is also possible here).

The detected reticulation events reflect the history of the Occitan language, which is a Romance language spoken in Southern France, Northern Italy and Eastern Spain [66]. There have been many interactions between Occitan and French since the Middle Ages. For instance, “Langue d’Oïl”, from which evolved the modern French, was spoken in the North, and “Langue d’Oc”, the ancestor of Occitan, was spoken in the South. Catalan, which is the closest relative of Occitan, is sometimes considered as one of its dialects [66, 67]. After the union of Aragon and Castile in 1479, the influence of the Iberian languages, in particular that of Spanish, on Catalan became more noticeable. Provençal is a dialect of Occitan spoken in Southern France [66].

The split graph obtained for the entire Latin group (Fig. 7a) represents a highly implicit linguistic network, which is not easy to interpret. For example, we could identify here Provençal as a lexical recipient with donors Catalan and the ancestor of French, Walloon and French Creole, as well as Italian as a lexical recipient with donors Ladin and Sardinian. No interpretable galled network has been obtained for the Latin language group.

### Network relationships within the Slavic group

Here we identified *Lusatian* as a lexical hybrid of *Polish* and *Czech* (both by word borrowing). The Sorbian (or Lusatian) languages are Slavic languages spoken in North East Germany [68]. These languages have been strongly influenced by Czech and Polish, since Lusatia is located at the border between Germany, the Czech Republic and Poland.

### Network relationships within the Persian and Sanskrit groups

Here we identified three possible lexical hybrids in two different program runs, i.e., one run for each of these groups:

- *Wakhi* as a recipient of lexical material from *Tadzik* (by word borrowing) and *Ossetic* (by word borrowing, but the inheritance from a close common ancestor is also possible here). Wakhi is an Iranian language spoken in Pamir, a mountain region between Pakistan, Afghanistan, China and Tajikistan. For the small nations of Pamir the language of oral and written communication is Tadzik. Moreover, the Wakhi oral tradition is bilingual (Wakhi and Tadzik), and most Wakhs speak Tadzik quite fluently [69].
- Ancestor of *Nepali* and *Khaskura* as a recipient of lexical material from *Hindi* (by word borrowing) and *Kashmiri* (by word borrowing). Nepali and Khaskura are spoken mainly in Nepal, India and Bhutan. They share about 80 % of their lexicon with Hindi [70].
- Ancestor of *Lahnda* and *Panjabi* as a recipient of lexical material from *Hindi* (by word borrowing, but the inheritance from a close common ancestor is also possible here) and *Romani* (by word borrowing). Lahnda and Panjabi are the languages spoken in Pakistan and India [71]. The Romani migrated from Northern India to Europe between the 6th and 11th centuries [72]. They had numerous interactions with Northern Indian, Iranian and European languages during their migrations.

### Network relationships within the Celtic and French/Iberian groups

We applied our algorithm to the union of the Celtic and French/Iberian groups excluding from our analysis the lexical hybrids that we had already identified when examining the Latin group alone, i.e., Catalan and Provençal. This way, we found that the *Breton* subgroup was a recipient of lexical material from *Old Welsh* (by word borrowing, but the inheritance from a close common ancestor is also possible here) and *Old French* (by word borrowing). The former reticulation shows a close etymological relationship between Welsh and Breton, whereas the latter accounts for the important number of words that Breton borrowed from Old French, namely in the 15^th^ and 16^th^ centuries [73].

### Network relationships within the West Germanic and French/Iberian groups

We also applied our algorithm to the union of the West Germanic and French/Iberian groups ruling out the lexical hybrids we had already detected in these groups, i.e., Catalan, Provençal, Sranan, Pennsylvania Dutch and Frisian. This allowed us to identify *English* as a recipient of lexical material from the *Old French* (by word borrowing) and *Old Dutch* (by word borrowing, but the inheritance from a close common ancestor is also possible here) subgroups. Mention that these two reticulations do not exclude the direct inheritance of Old English from the Anglo-Frisian and North Germanic dialects originally spoken by Germanic tribes, traditionally known as the Angles, Saxons and Jutes [74]. Moreover, the relationship between Dutch and English originates in Old Saxon, which was spoken in North West Germany and in the Netherlands by Saxon peoples. Old Saxon was closely related to both Old English and Old Dutch [75]. After the Norman conquest of England in the 11th century, many French words were borrowed by Middle English. Furthermore, English was replaced as the language of the upper classes by Anglo-Norman, a relative of Old French, and Old English developed into the next historical form of English, known as the Middle English language [74].

### Network relationships between IE language groups

In our final analysis, we removed from our data set the 12 lexical hybrids already identified in the original set of 84 IE languages, thus obtaining a reduced distance matrix **D**_72_ of size (72×72). We applied our algorithm to this reduced matrix and limited the search of recipient and donor languages to the ancestor branches of the 11 main IE language groups (Armenian, Albanian, Baltic, Celtic, Greek, Latin, North Germanic, Persian, Sanskrit, Slavic and West Germanic).

First, we identified the Armenian group as a recipient of lexical material from the Albanian and Persian groups, and, second, the Albanian group as a recipient of lexical material from the Sanskrit and Latin groups. Since the reticulation (hybridization) score, which reflect the likelihood of a reticulation event (see Formula 3), of Albanian was much higher than that of Armenian, we applied our algorithm once again after removing from the distance matrix the data corresponding to the five languages of the Albanian group. It is worth noting that the position of the Albanian group in the IE language tree has been found to be unstable by many authors [7–9, 26]. The following application of our method to the reduced distance matrix **D**_67_ of size (67×67) allowed us to identify Old Armenian as a recipient of lexical material from Old Persian and Old Greek (Fig. 4). A similar network pattern was found by the galled network method (Fig. 9). Thus, we could identify here:

- *Old Albanian* as a recipient of lexical material from *Sanskrit* (by word borrowing, but the inheritance from a close common ancestor is also possible here) and *Latin* (by word borrowing). Albanian borrowed many words from Latin, in particular between the 2nd century B.C. and the 5th century A.D. [76]. The Albanian group is also a close relative of the union of the Sanskrit and Persian in the IE language tree (see for example Fig. 1 in [7]).

- *Old Armenian* as a recipient of lexical material from *Old Greek* (by word borrowing, but the inheritance from a close common ancestor is also possible here) and *Old Persian* (by word borrowing). The Armenians stayed under Persian rule for long periods of time from the 5th century BC to the 19th century AC and the Armenian language includes a large number of Iranian loanwords in its vocabulary [77]. Moreover, the well-known “Graeco-Armenian” hypothesis postulates that Armenian is the closest relative of Greek [78].

## Conclusion

The application of computational biology methods presented here in the context of historical linguistic can be viewed as a step towards a better understanding of the evolution of natural languages [79–82]. In this paper, we adapted a recently developed bioinformatics method for inferring explicit hybridization networks [39] to identify reticulate relationships between languages. We also showed how the well-known split graph [43, 44] and galled network [45] algorithms can be applied to analyze linguistic data. While all the three competing methods can be used to reconstruct evolutionary relationships between natural languages, our method has the important advantage of identifying these relationships explicitly. It also allows one to establish the extent of influence of each of the donor languages on the corresponding recipient languages through the computation of the reticulation degree parameter. Some recent studies have used syntactic distances to infer phylogenies of IE languages [83, 84]. Syntactic parameters reveal complementary relationships between languages which are often not reflected by lexicon [83]. This type of syntactic distances could be further used to refine the inference of linguistic networks along with plausible phonological and morphological data. It would be also interesting to extend our method to infer the exact timing of the obtained reticulation events. This will allow us to discover new historical events that have shaped the evolution of natural languages.

## Additional files

Additional file 1: Biolinguistic IE data archive. This file includes phonetic data, data matrices, Newick strings and word trees discussed in this paper as well as Perl and Python scripts for computing the Levenshtein and SCA distances. (ZIP 328 kb)
