# Supplementary material for: Using hybridization networks to retrace the evolution of Indo-European languages
Source: BMC Evol Biol. 2016 Sep 6;16(1):180. doi: 10.1186/s12862-016-0745-6 (PMC5012036; doi:10.1186/s12862-016-0745-6)
Supplement: Additional file 1: — Biolinguistic IE data archive. This file includes phonetic data, data matrices, Newick strings and word trees discussed in this paper as well as Perl and Python scripts for computing the Levenshtein and SCA distances. (ZIP 328 kb) [file 12862_2016_745_MOESM1_ESM.zip › Word_trees.pdf]

Phylogenetic trees obtained with SCA phonetic distance (List, 2012) - in blue and with weighted Levenshtein distance - in red. The Neighbor-Joining (Saitou et Nei, 1987) algorithm was applied to infer the trees from the distance matrices. All the data have been taken from the IELex database (http://ielex.mpi.nl/) (Dunn *et al.* 2011).

Animal

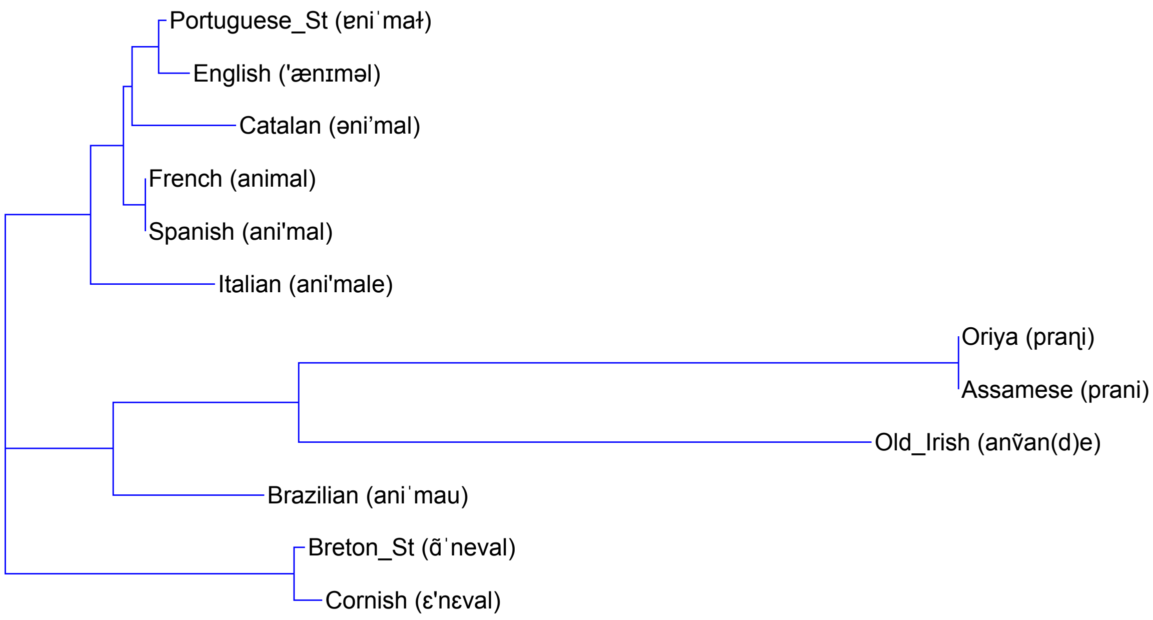

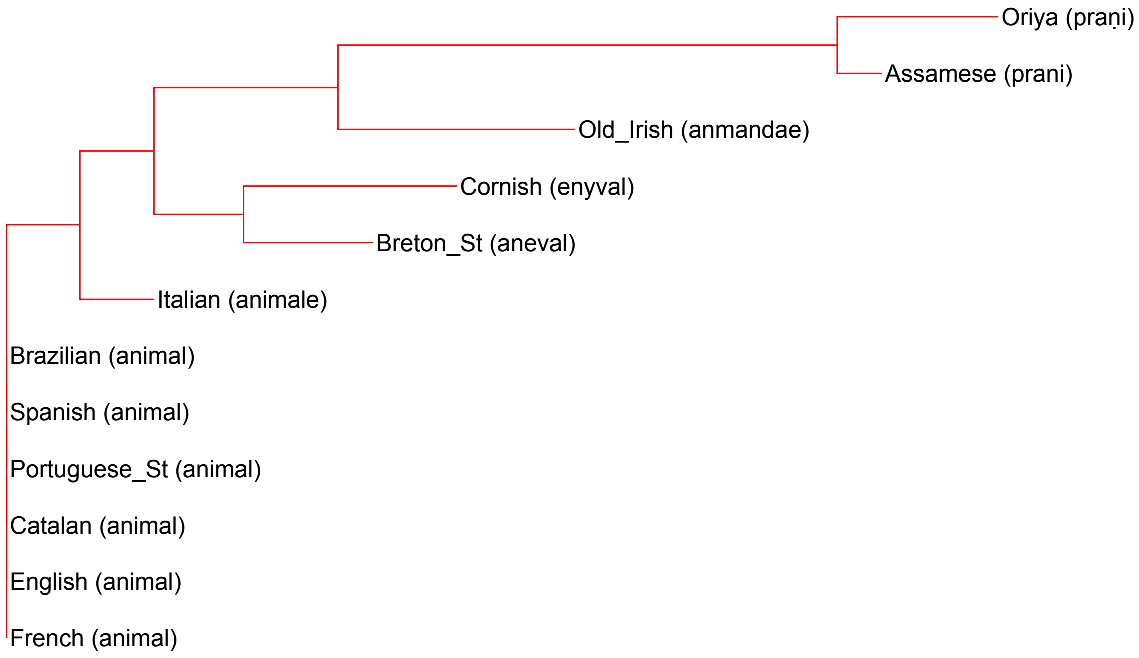

Bark

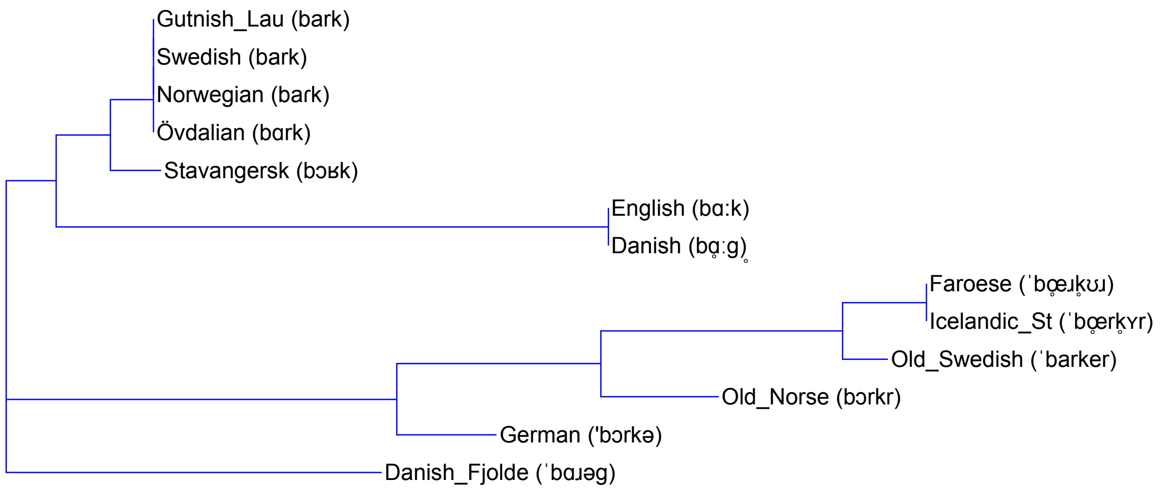
Gutnish_Lau (bark)
Swedish (bark)
Norwegian (bark)
Övdalian (bɑrk)
Stavangersk (bɔʁk)
English (bɑːk)
Danish (bɑ̥ːg̊)
Faroese (ˈbœ̥ɹk̥ʊɹ)
Icelandic_St (ˈbœ̥rk̥ʏr)
Old_Swedish (ˈbarker)
Old_Norse (bɔrkr)
German ('bɔrkə)
Danish_Fjolde (ˈbɑɹəg)

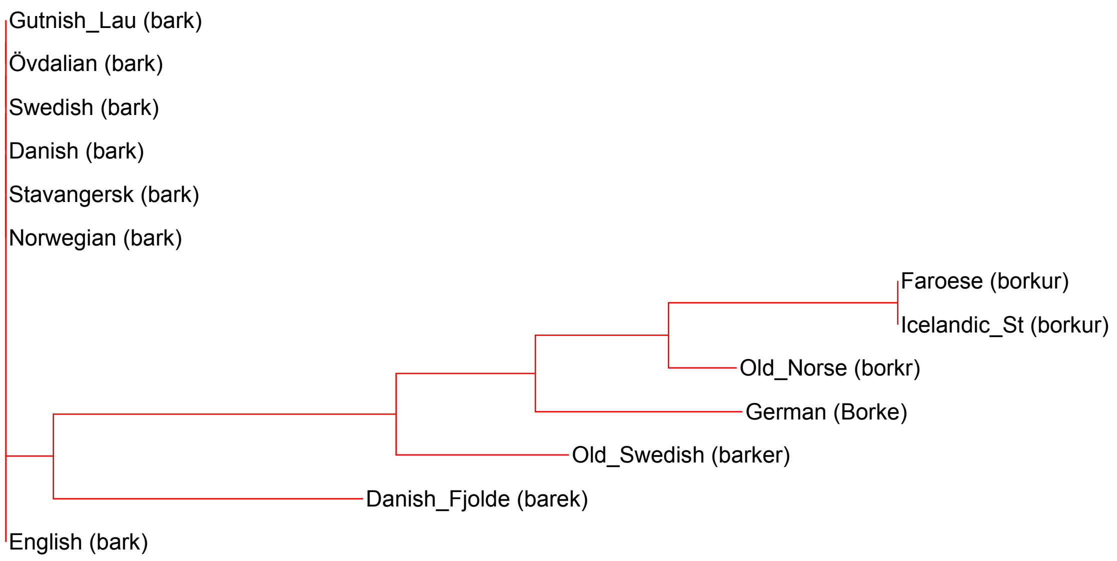
Gutnish_Lau (bark)
Övdalian (bark)
Swedish (bark)
Danish (bark)
Stavangersk (bark)
Norwegian (bark)
Faroese (borkur)
Icelandic_St (borkur)
Old_Norse (borkr)
German (Borke)
Old_Swedish (barker)
Danish_Fjolde (barek)
English (bark)

Belly

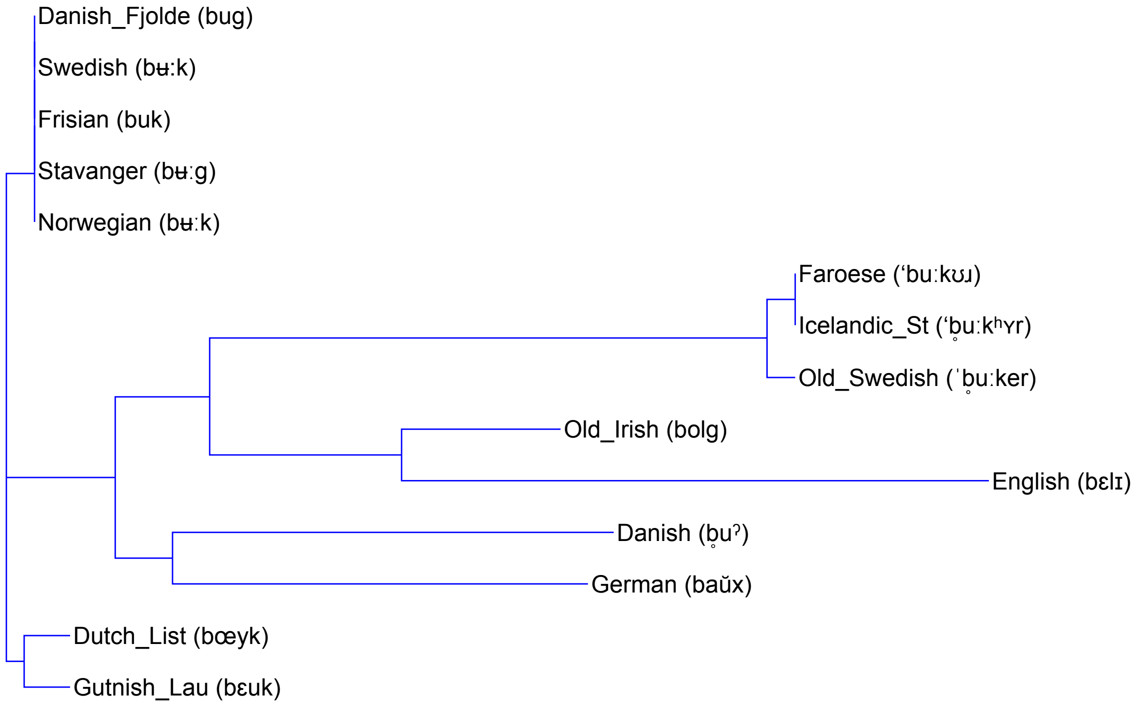
Danish_Fjolde (bug)
Swedish (bʉːk)
Frisian (buk)
Stavanger (bʉːg)
Norwegian (bʉːk)
Faroese (ʻbuːkʊɹ)
Icelandic_St (ʻbu̥ːkʰʏr)
Old_Swedish (ˈbu̥ːker)
Old_Irish (bolg)
English (bɛlɪ)
Danish (bu̥ˀ)
German (baŭx)
Dutch_List (bœyk)
Gutnish_Lau (bɛuk)

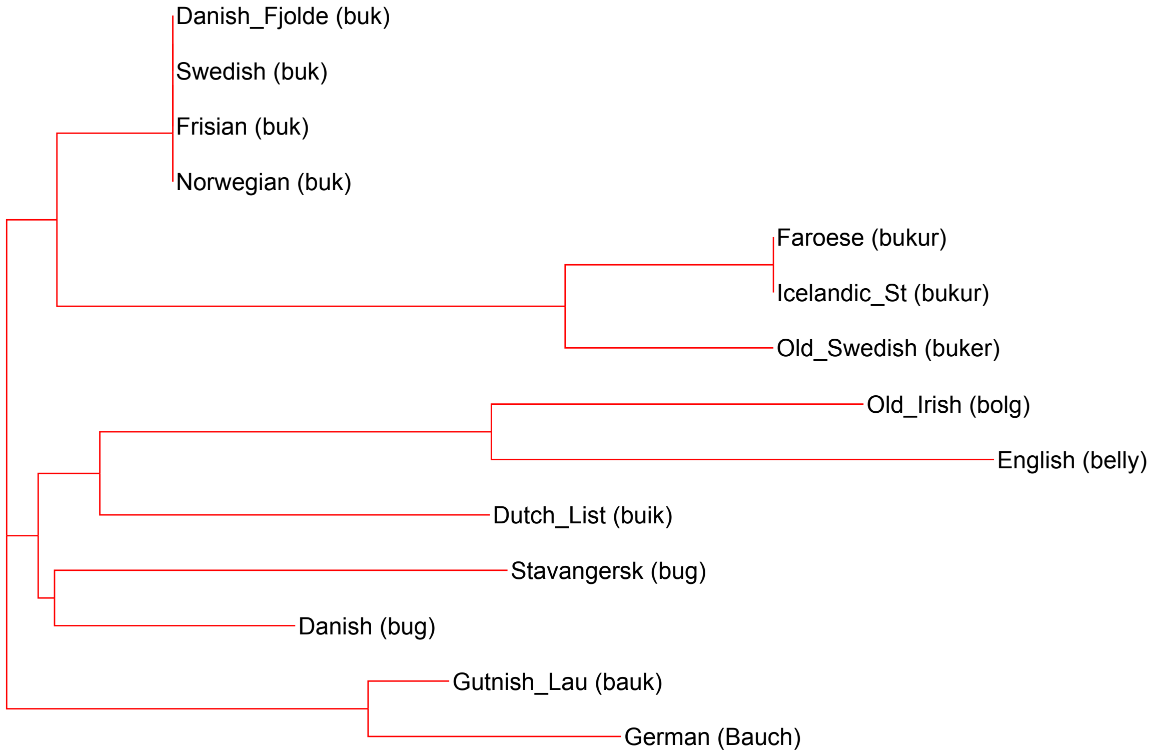
Danish_Fjolde (buk)
Swedish (buk)
Frisian (buk)
Norwegian (buk)
Faroese (bukur)
Icelandic_St (bukur)
Old_Swedish (buker)
Old_Irish (bolg)
English (belly)
Dutch_List (buik)
Stavangersk (bug)
Danish (bug)
Gutnish_Lau (bauk)
German (Bauch)

Count

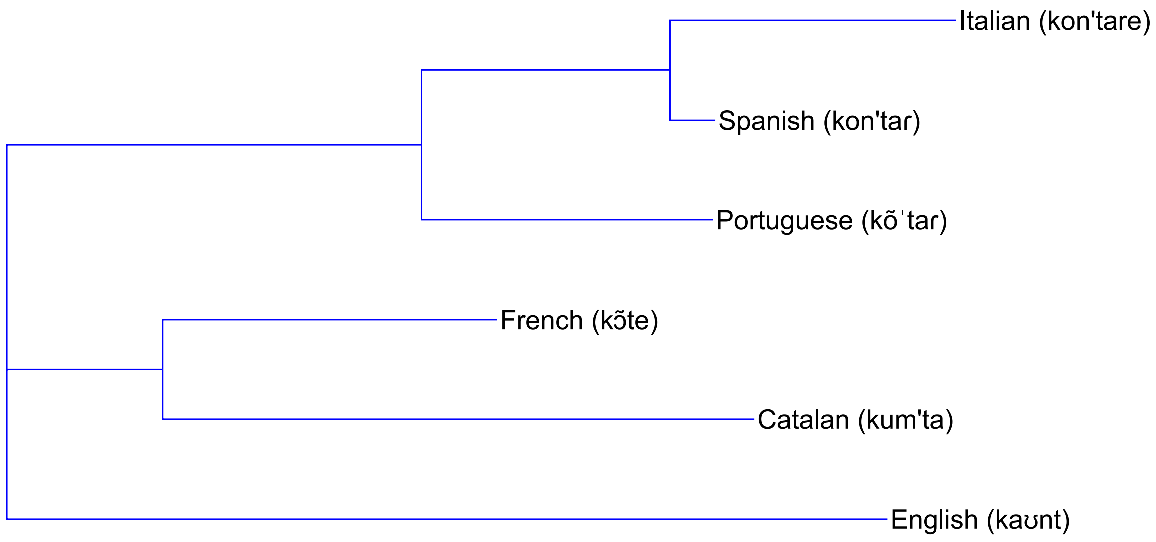
Italian (kon'tare)
Spanish (kon'tar)
Portuguese (kõˈtar)
French (kõte)
Catalan (kum'ta)
English (kaʊnt)

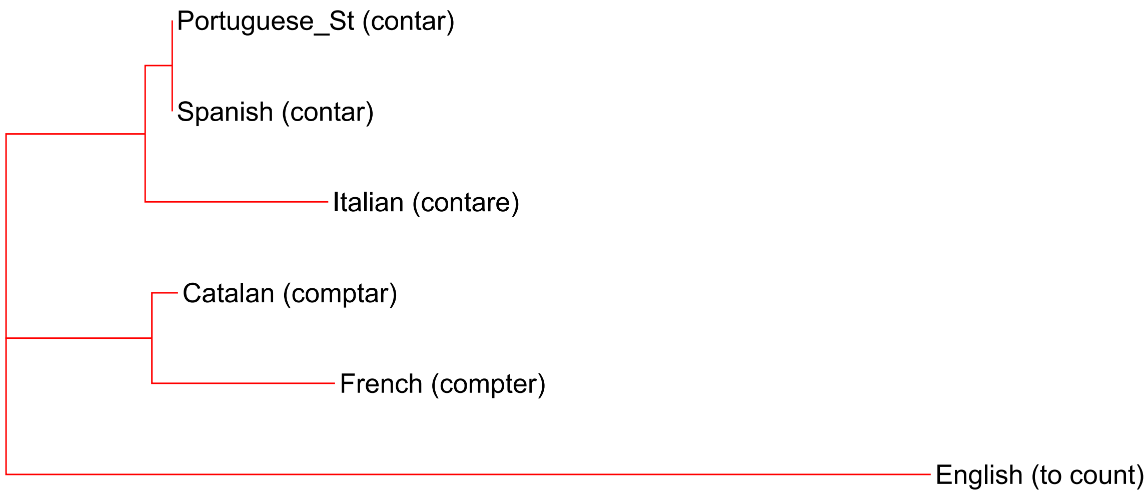
Portuguese_St (contar)
Spanish (contar)
Italian (contare)
Catalan (comptar)
French (compter)
English (to count)

Die

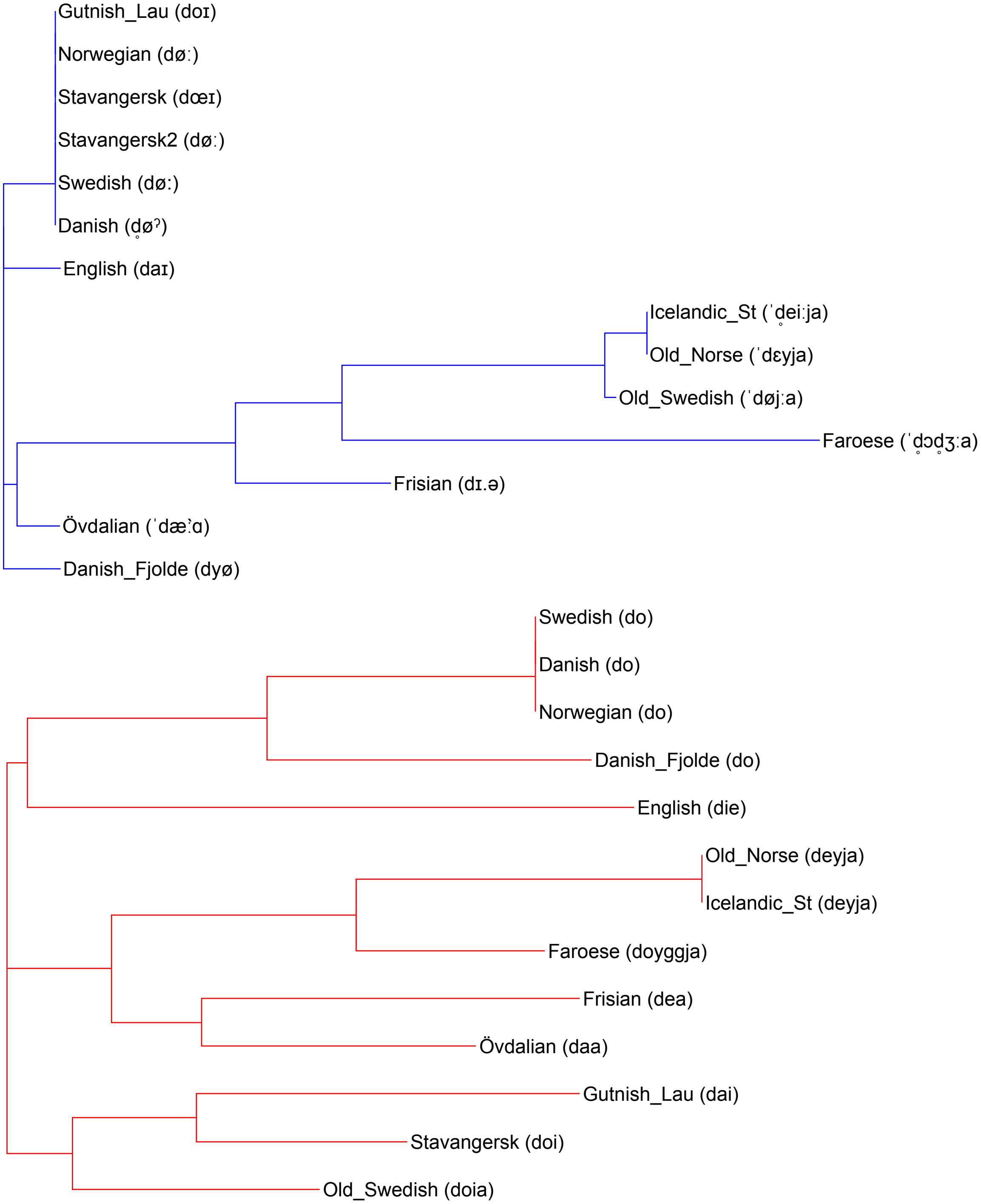
Gutnish_Lau (doɪ)
Norwegian (dø:)
Stavangersk (dœɪ)
Stavangersk2 (dø:)
Swedish (dø:)
Danish (d̥ø̰ˀ)
English (daɪ)
Icelandic_St (ˈd̥ei:ja)
Old_Norse (ˈdɛyja)
Old_Swedish (ˈdøj:a)
Faroese (ˈd̥ɔd̥ʒ:a)
Frisian (dɪ.ə)
Övdalian (ˈdæ:ɑ)
Danish_Fjolde (dyø)
Swedish (do)
Danish (do)
Norwegian (do)
Danish_Fjolde (do)
English (die)
Old_Norse (deyja)
Icelandic_St (deyja)
Faroese (doyggja)
Frisian (dea)
Övdalian (daa)
Gutnish_Lau (dai)
Stavangersk (doi)
Old_Swedish (doia)

Dust

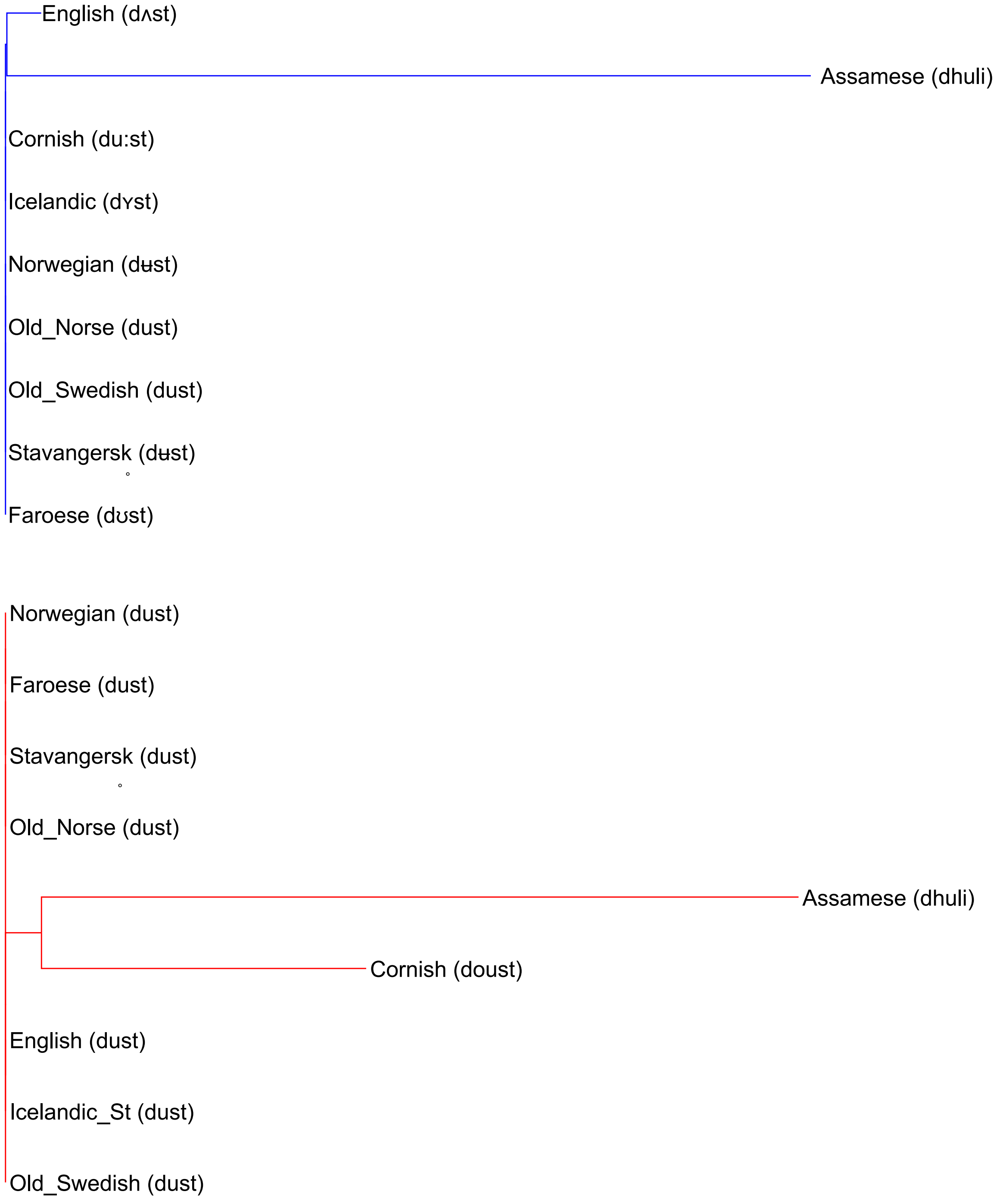
English (dʌst)
Assamese (dhuli)
Cornish (du:st)
Icelandic (dʏst)
Norwegian (dʉst)
Old_Norse (dust)
Old_Swedish (dust)
Stavangersk (dʉst)
Faroese (dʊst)
Norwegian (dust)
Faroese (dust)
Stavangersk (dust)
Old_Norse (dust)
Assamese (dhuli)
Cornish (doust)
English (dust)
Icelandic_St (dust)
Old_Swedish (dust)

Egg

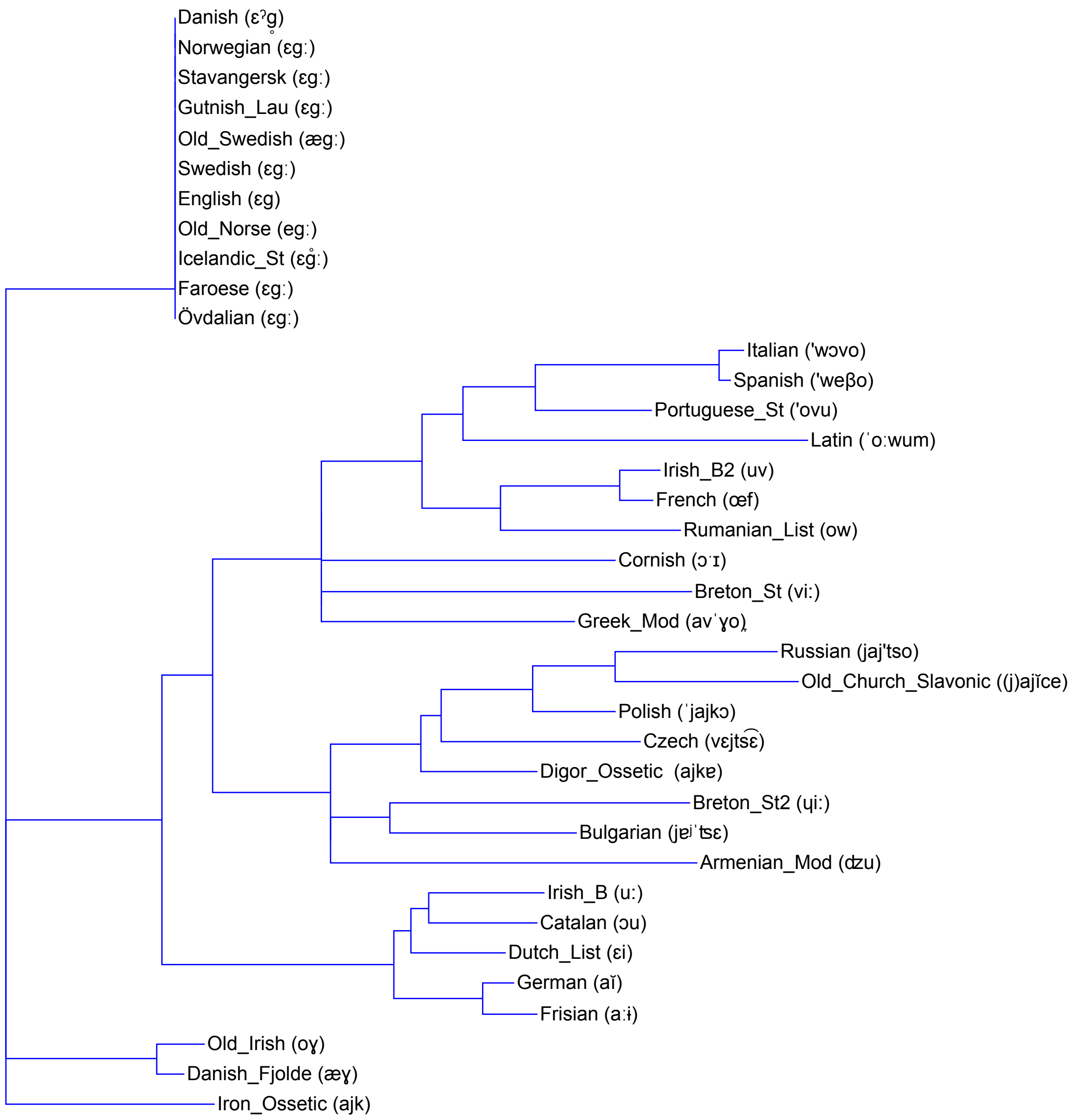

Danish (ɛˀg̊)
Norwegian (ɛgː)
Stavangersk (ɛgː)
Gutnish_Lau (ɛgː)
Old_Swedish (æɡː)
Swedish (ɛgː)
English (ɛg)
Old_Norse (egː)
Icelandic_St (ɛg̊ː)
Faroese (ɛgː)
Övdalian (ɛgː)
Italian ('wɔvo)
Spanish ('weβo)
Portuguese_St ('ovu)
Latin (ˈoːwum)
Irish_B2 (uv)
French (œf)
Rumanian_List (ow)
Cornish (ɔˑɪ)
Breton_St (viː)
Greek_Mod (avˈɣo)
Russian (jaj'tso)
Old_Church_Slavonic ((j)ajĭce)
Polish (ˈjajkɔ)
Czech (vɛjt͡sɛ)
Digor_Ossetic (ajkɐ)
Breton_St2 (ɥiː)
Bulgarian (jəʲˈtsɛ)
Armenian_Mod (dzu)
Irish_B (uː)
Catalan (ɔu)
Dutch_List (ɛi)
German (aĭ)
Frisian (aːɨ)
Old_Irish (oɣ)
Danish_Fjolde (æɣ)
Iron_Ossetic (ajk)

Egg

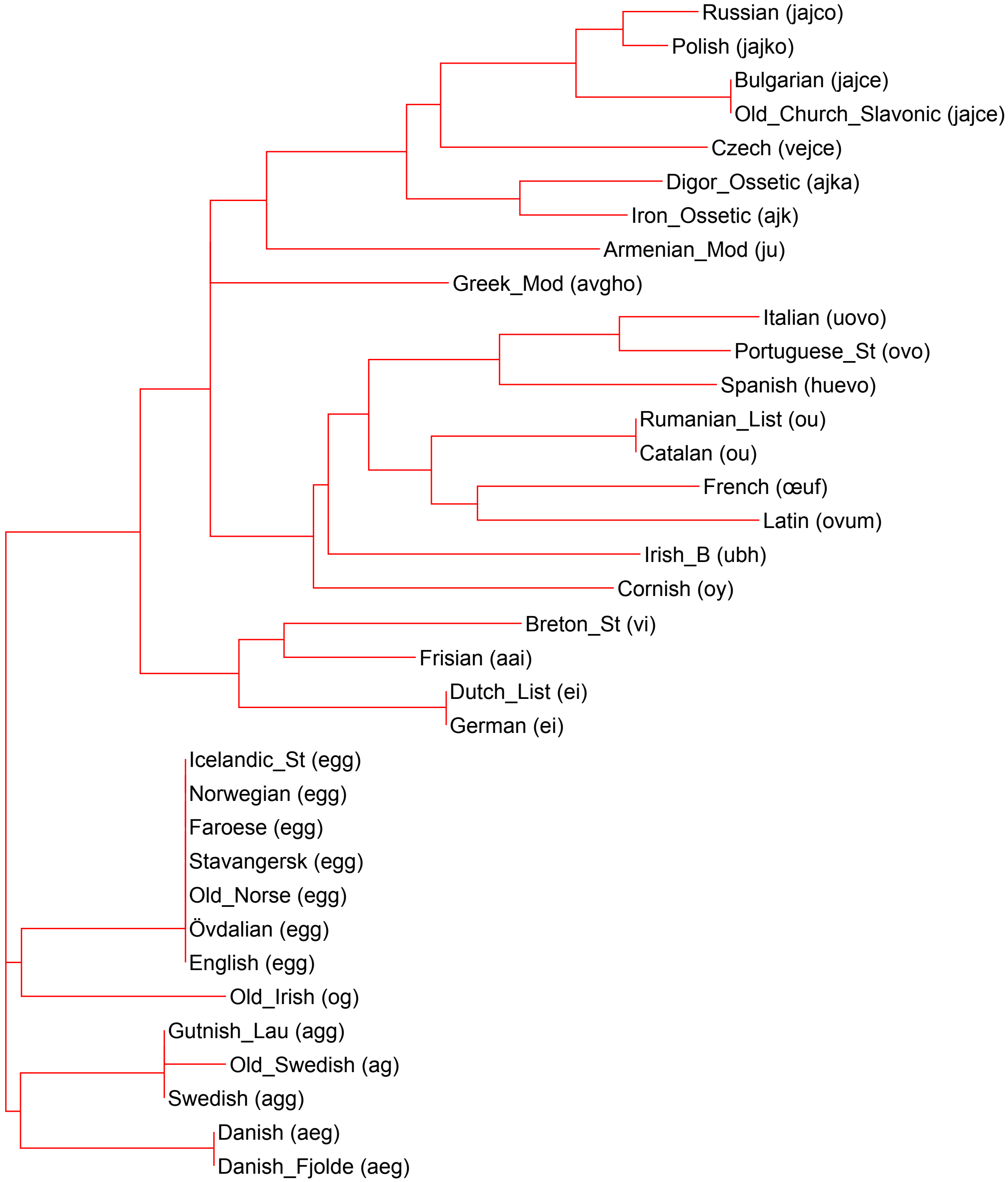
Russian (jajco)
Polish (jajko)
Bulgarian (jajce)
Old_Church_Slavonic (jajce)
Czech (vejce)
Digor_Ossetic (ajka)
Iron_Ossetic (ajk)
Armenian_Mod (ju)
Greek_Mod (avgho)
Italian (uovo)
Portuguese_St (ovo)
Spanish (huevo)
Rumanian_List (ou)
Catalan (ou)
French (œuf)
Latin (ovum)
Irish_B (ubh)
Cornish (oy)
Breton_St (vi)
Frisian (aai)
Dutch_List (ei)
German (ei)
Icelandic_St (egg)
Norwegian (egg)
Faroese (egg)
Stavangersk (egg)
Old_Norse (egg)
Övdalian (egg)
English (egg)
Old_Irish (og)
Gutnish_Lau (agg)
Old_Swedish (ag)
Swedish (agg)
Danish (aeg)
Danish_Fjolde (aeg)

Few

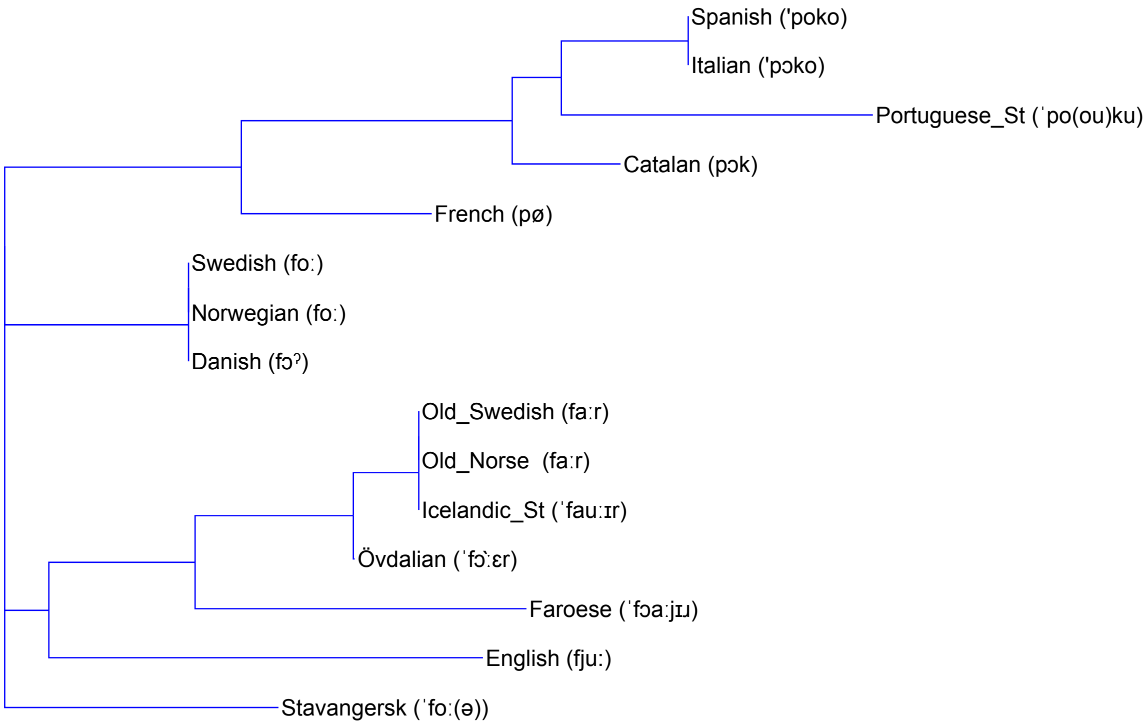
Spanish ('poko)
Italian ('pɔko)
Portuguese_St (ˈpo(ou)ku)
Catalan (pɔk)
French (pø)
Swedish (fo:)
Norwegian (fo:)
Danish (fɔˀ)
Old_Swedish (fa:r)
Old_Norse (fa:r)
Icelandic_St (ˈfau:ɪr)
Övdalian (ˈfɔ̀:ɛr)
Faroese (ˈfɔa:jɪɹ)
English (fju:)
Stavangersk (ˈfo:(ə))

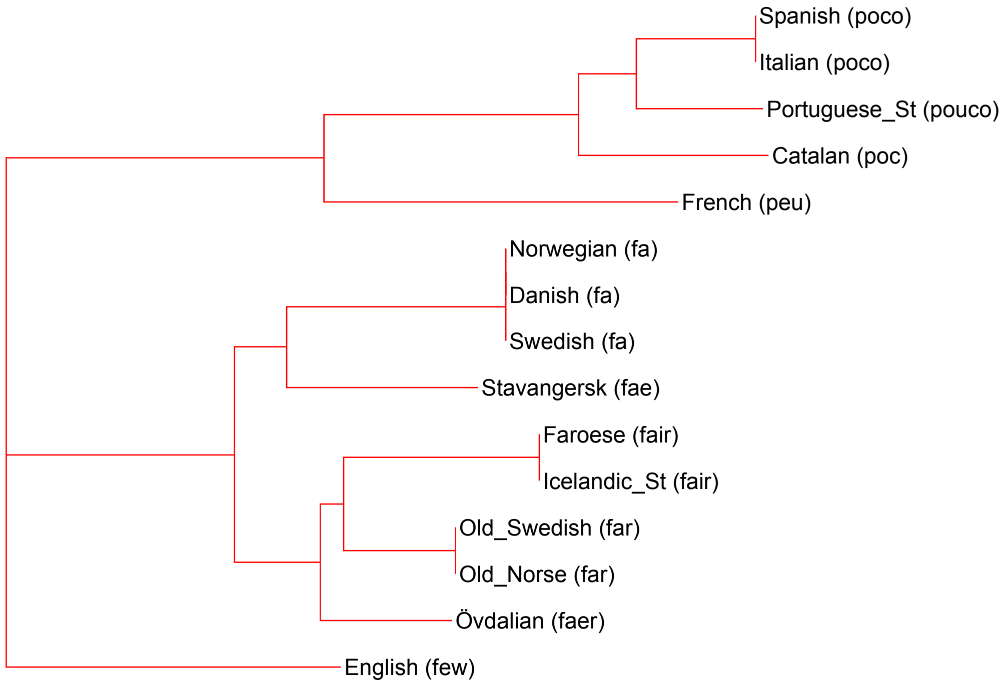
Spanish (poco)
Italian (poco)
Portuguese_St (pouco)
Catalan (poc)
French (peu)
Norwegian (fa)
Danish (fa)
Swedish (fa)
Stavangersk (fae)
Faroese (fair)
Icelandic_St (fair)
Old_Swedish (far)
Old_Norse (far)
Övdalian (faer)
English (few)

Flower

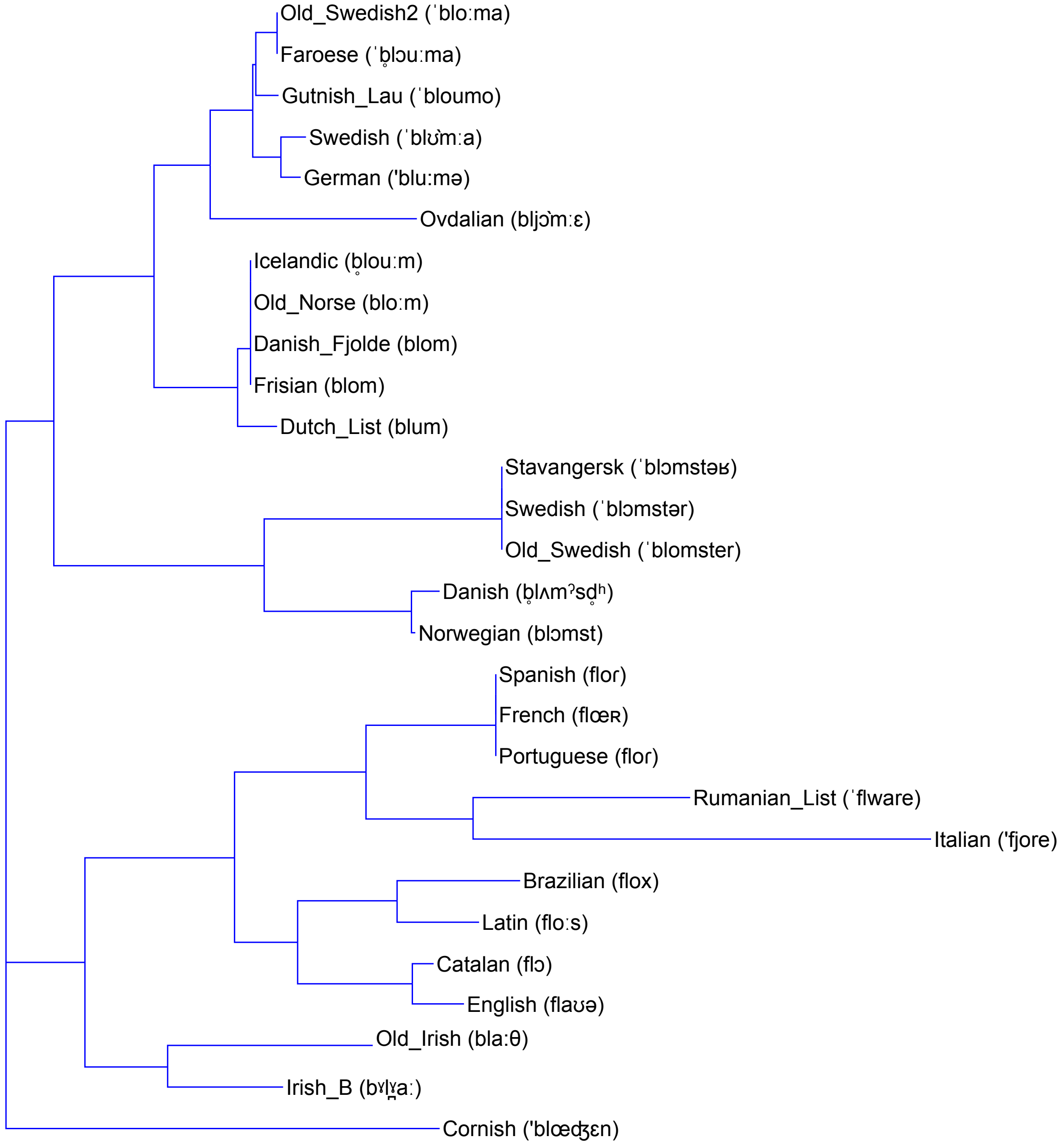
Old_Swedish2 (ˈbloːma)
Faroese (ˈb̥lɔuːma)
Gutnish_Lau (ˈbloumo)
Swedish (ˈblʊ̌mːa)
German ('bluːmə)
Ovdalian (bljɔ̌mːɛ)
Icelandic (b̥louːm)
Old_Norse (bloːm)
Danish_Fjolde (blom)
Frisian (blom)
Dutch_List (blum)
Stavangersk (ˈblɔmstəʁ)
Swedish (ˈblɔmstər)
Old_Swedish (ˈblomster)
Danish (b̥lʌmˀsd̥ʰ)
Norwegian (blɔmst)
Spanish (flor)
French (flœʀ)
Portuguese (flor)
Rumanian_List (ˈflware)
Italian ('fjore)
Brazilian (flox)
Latin (floːs)
Catalan (flɔ)
English (flaʊə)
Old_Irish (blaːθ)
Irish_B (bˠlʲa̪ː)
Cornish ('blœʤɛn)

Flower

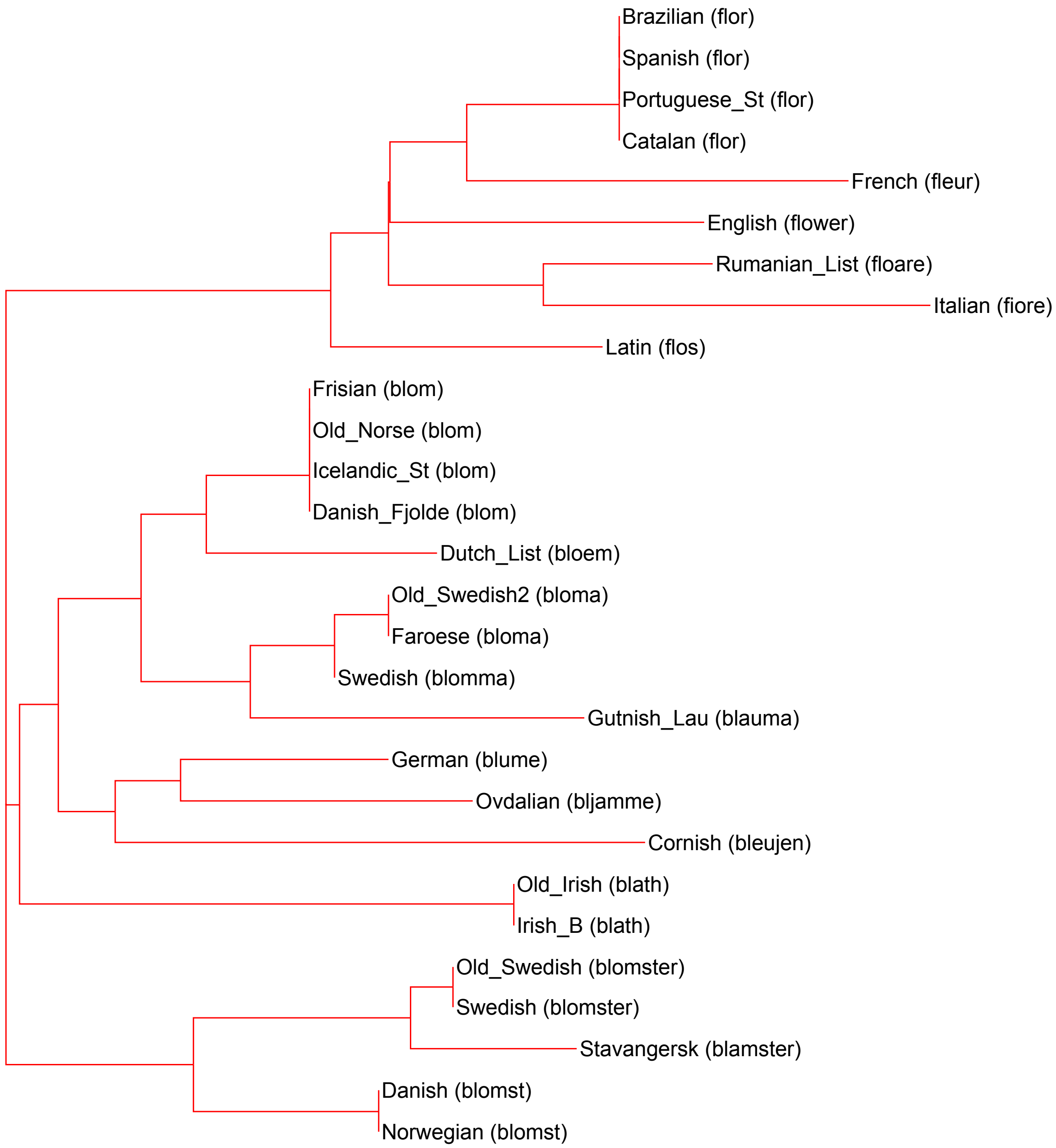

Brazilian (flor)
Spanish (flor)
Portuguese_St (flor)
Catalan (flor)
French (fleur)
English (flower)
Rumanian_List (floare)
Italian (fiore)
Latin (flos)
Frisian (blom)
Old_Norse (blom)
Icelandic_St (blom)
Danish_Fjolde (blom)
Dutch_List (bloem)
Old_Swedish2 (bloma)
Faroese (bloma)
Swedish (blomma)
Gutnish_Lau (blauma)
German (blume)
Ovdalian (bljamme)
Cornish (bleujen)
Old_Irish (blath)
Irish_B (blath)
Old_Swedish (blomster)
Swedish (blomster)
Stavangersk (blamster)
Danish (blomst)
Norwegian (blomst)

Fruit

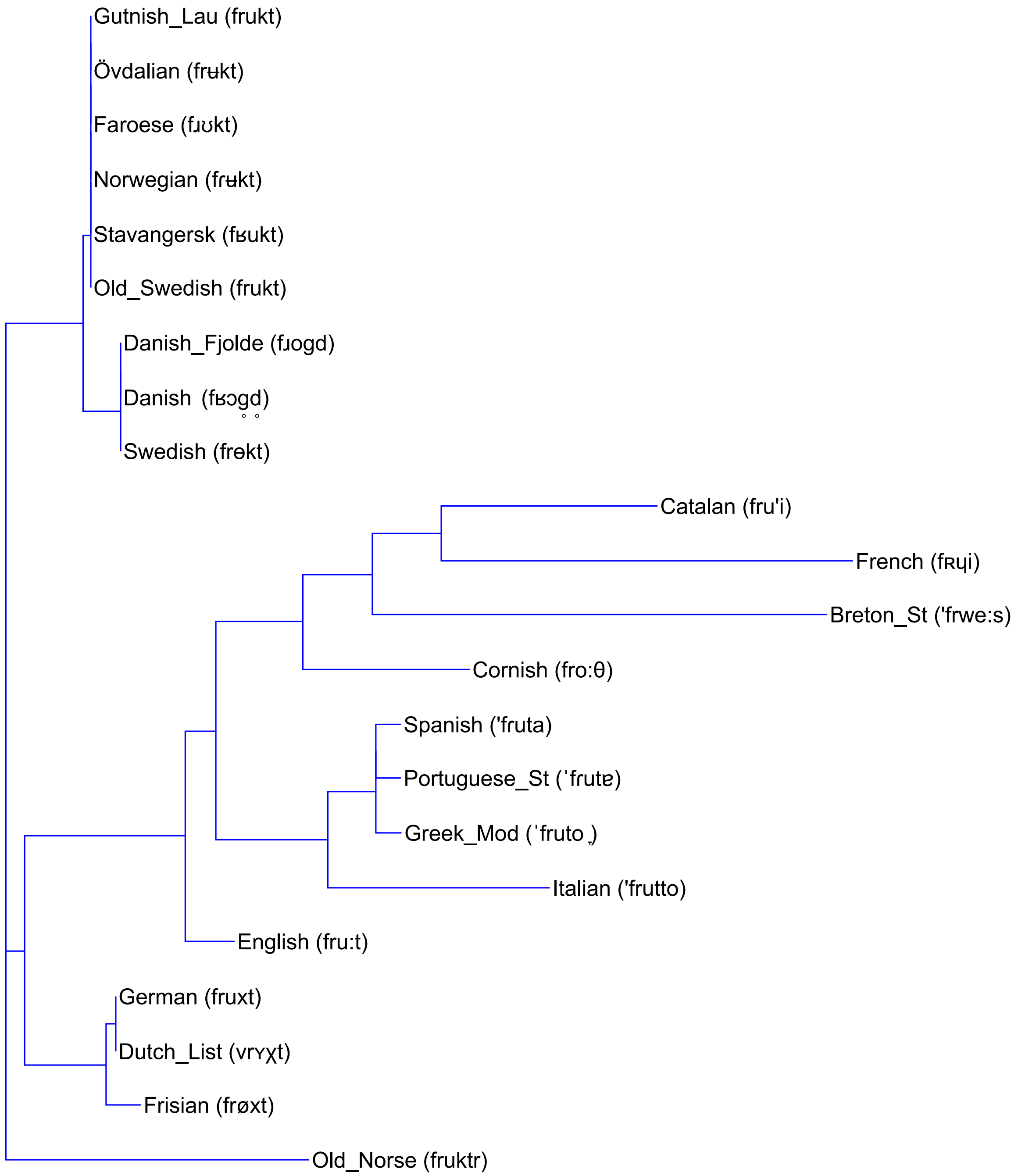
Gutnish_Lau (frukt)
Övdalian (frʉkt)
Faroese (fɹʊkt)
Norwegian (frʉkt)
Stavangersk (fʁukt)
Old_Swedish (frukt)
Danish_Fjolde (fɹogd)
Danish (fʁɔg̥d̥)
Swedish (frɵkt)
Catalan (fru'i)
French (fʀɥi)
Breton_St ('frwe:s)
Cornish (fro:θ)
Spanish ('fruta)
Portuguese_St (ˈfrutɐ)
Greek_Mod (ˈfruto̞)
Italian ('frutto)
English (fru:t)
German (fruxt)
Dutch_List (vrʏχt)
Frisian (frøxt)
Old_Norse (fruktr)

Fruit

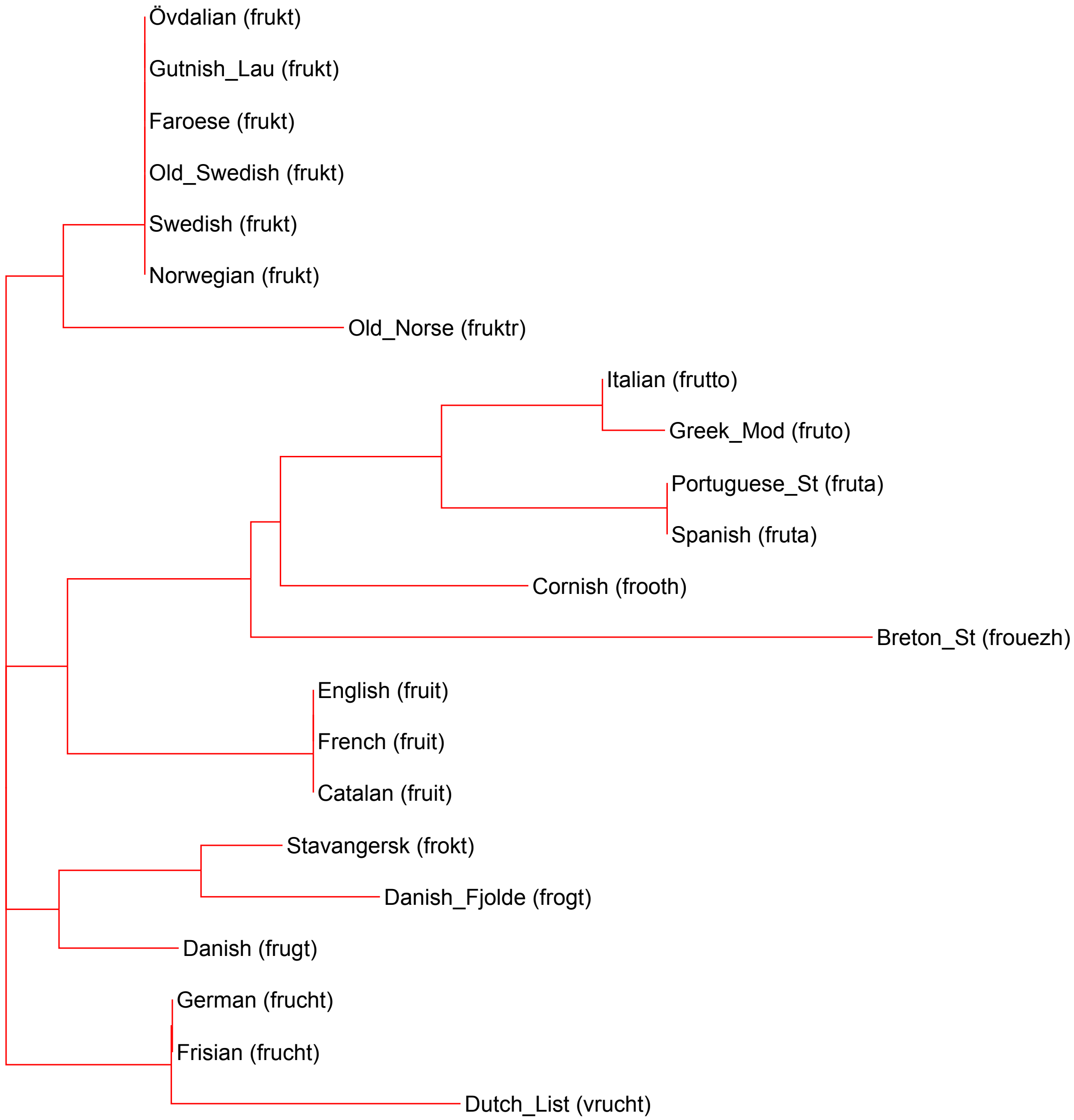

Övdalian (frukt)
Gutnish_Lau (frukt)
Faroese (frukt)
Old_Swedish (frukt)
Swedish (frukt)
Norwegian (frukt)
Old_Norse (fruktr)
Italian (frutto)
Greek_Mod (fruto)
Portuguese_St (fruta)
Spanish (fruta)
Cornish (frooth)
Breton_St (frouezh)
English (fruit)
French (fruit)
Catalan (fruit)
Stavangersk (frokt)
Danish_Fjolde (frogt)
Danish (frugt)
German (frucht)
Frisian (frucht)
Dutch_List (vrucht)

Give

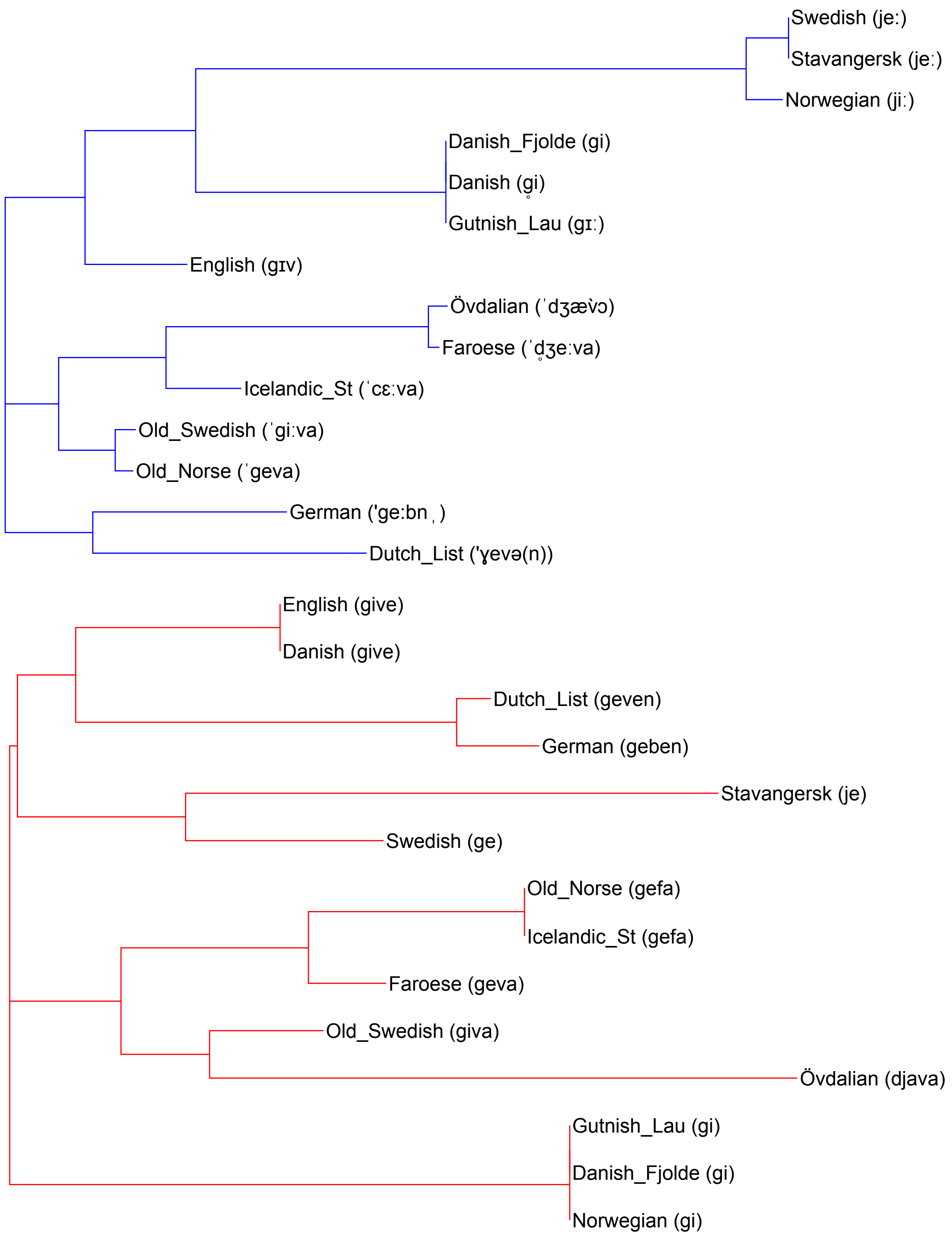
Swedish (je:)
Stavangersk (je:)
Norwegian (ji:)
Danish_Fjolde (gi)
Danish (gi̥)
Gutnish_Lau (gɪ:)
English (gɪv)
Övdalian (ˈdʒæ̀vɔ)
Faroese (ˈd̥ʒe:va)
Icelandic_St (ˈcɛ:va)
Old_Swedish (ˈgi:va)
Old_Norse (ˈgeva)
German ('ge:bn̩)
Dutch_List ('ɣevə(n))
English (give)
Danish (give)
Dutch_List (geven)
German (geben)
Stavangersk (je)
Swedish (ge)
Old_Norse (gefa)
Icelandic_St (gefa)
Faroese (geva)
Old_Swedish (giva)
Övdalian (djava)
Gutnish_Lau (gi)
Danish_Fjolde (gi)
Norwegian (gi)

Husband

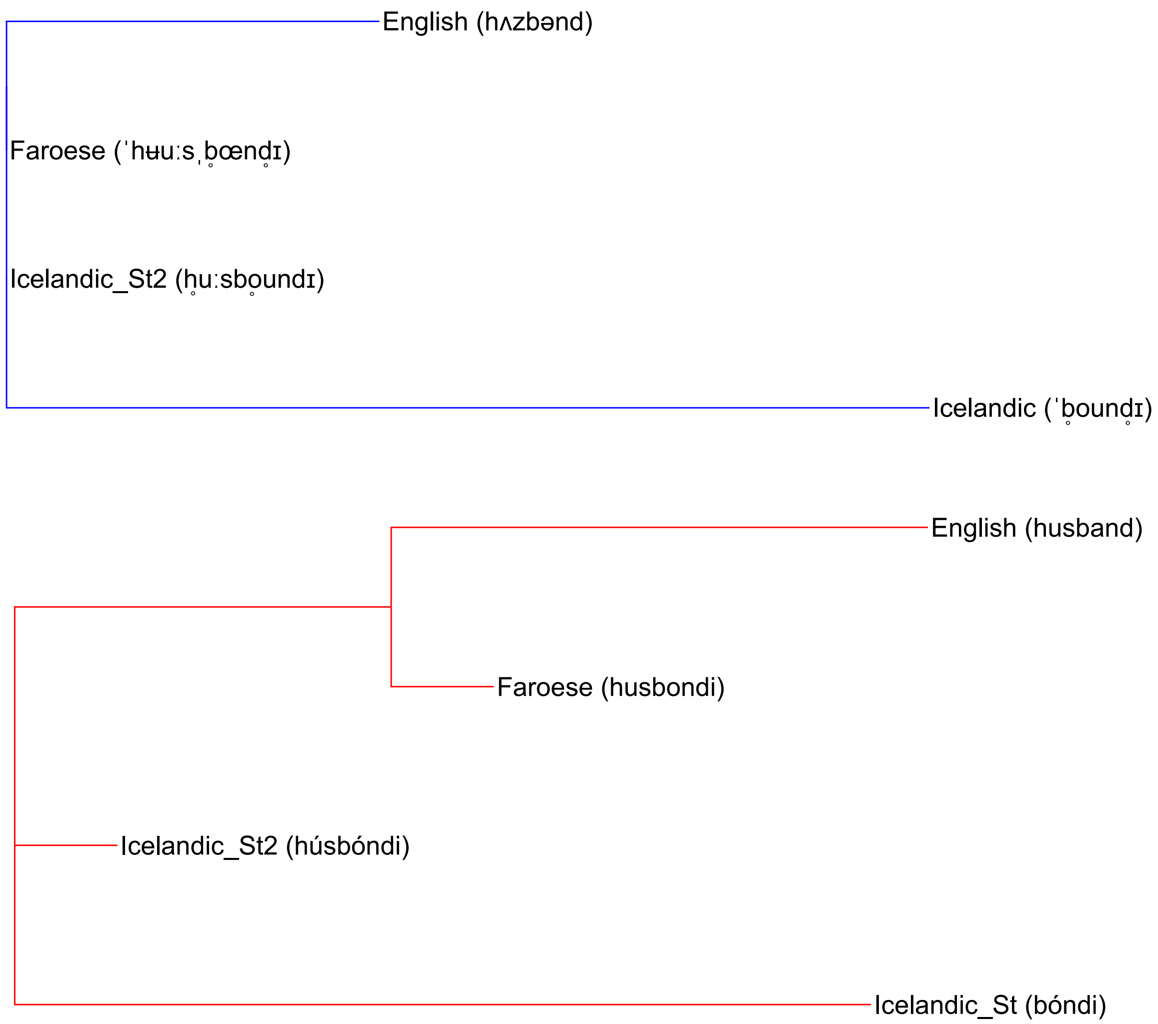
English (hʌzbənd)
Faroese (ˈhʉuːsˌb̥œnd̥ɪ)
Icelandic_St2 (h̥uːsb̥oundɪ)
Icelandic (ˈb̥ound̥ɪ)
English (husband)
Faroese (husbondi)
Icelandic_St2 (húsbóndi)
Icelandic_St (bóndi)

Lake

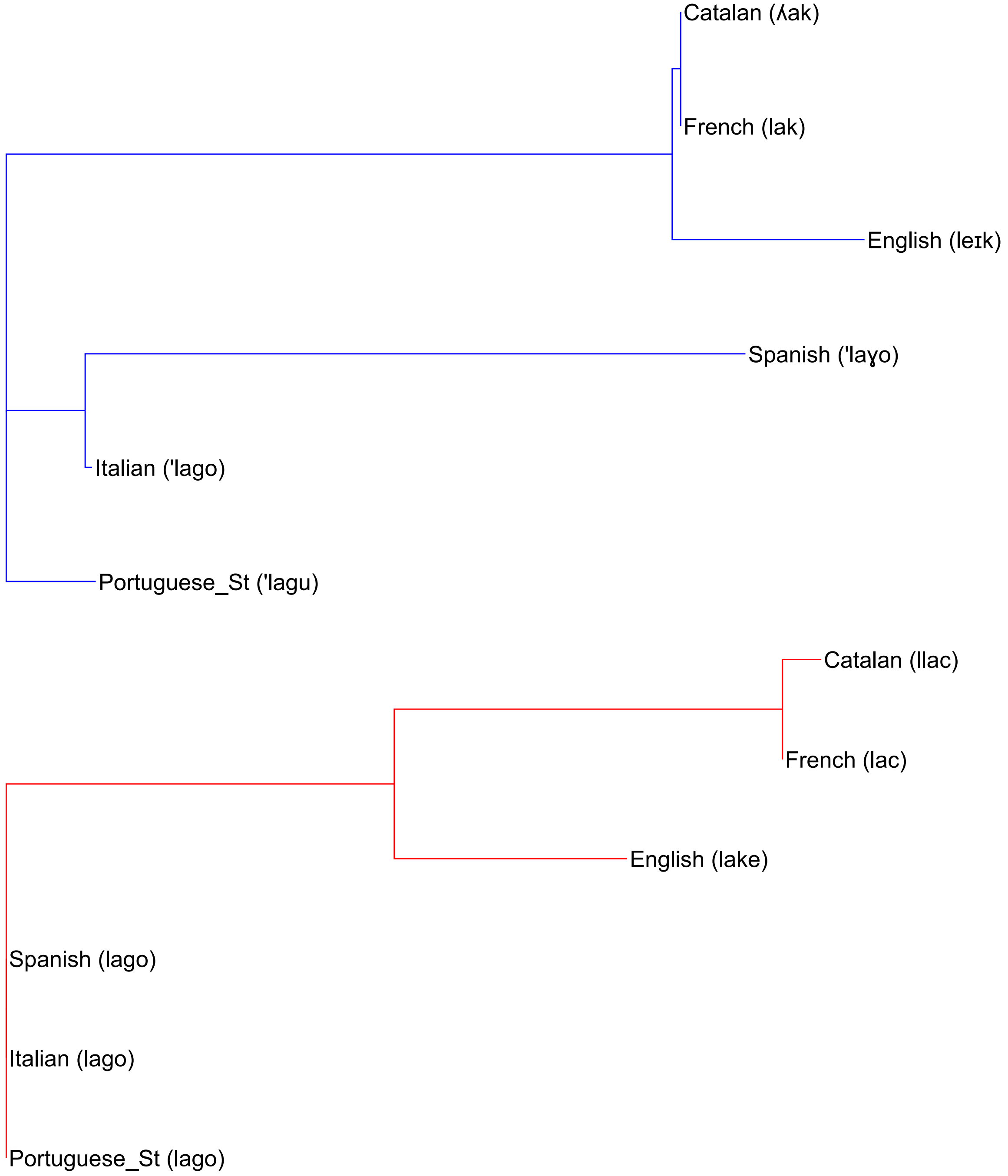
Catalan (ʎak)
French (lak)
English (leɪk)
Spanish ('laɣo)
Italian ('lago)
Portuguese_St ('lagu)
Catalan (llac)
French (lac)
English (lake)
Spanish (lago)
Italian (lago)
Portuguese_St (lago)

Leg

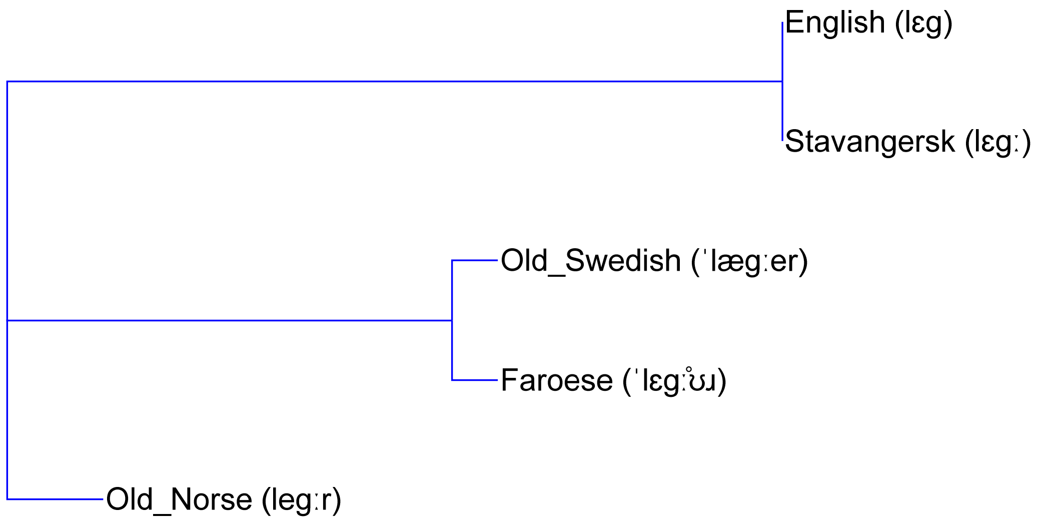
English (lɛg)
Stavangersk (lɛgː)
Old_Swedish (ˈlægːer)
Faroese (ˈlɛgːʊ̊ɹ)
Old_Norse (legːr)

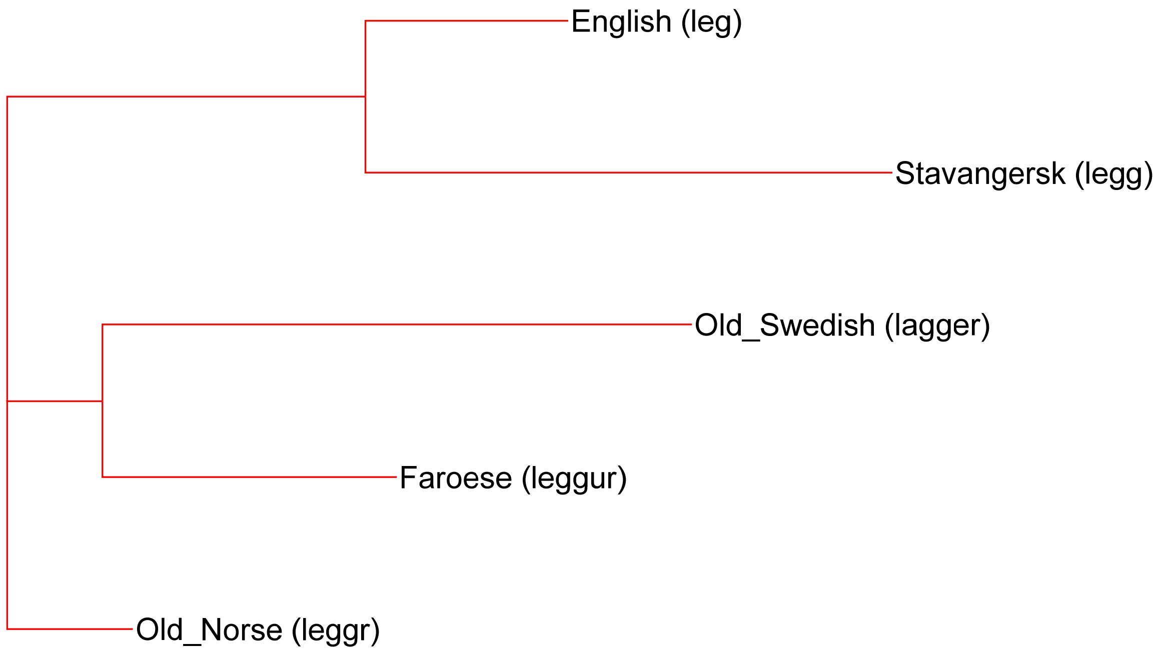
English (leg)
Stavangersk (legg)
Old_Swedish (lagger)
Faroese (leggur)
Old_Norse (leggr)

Many

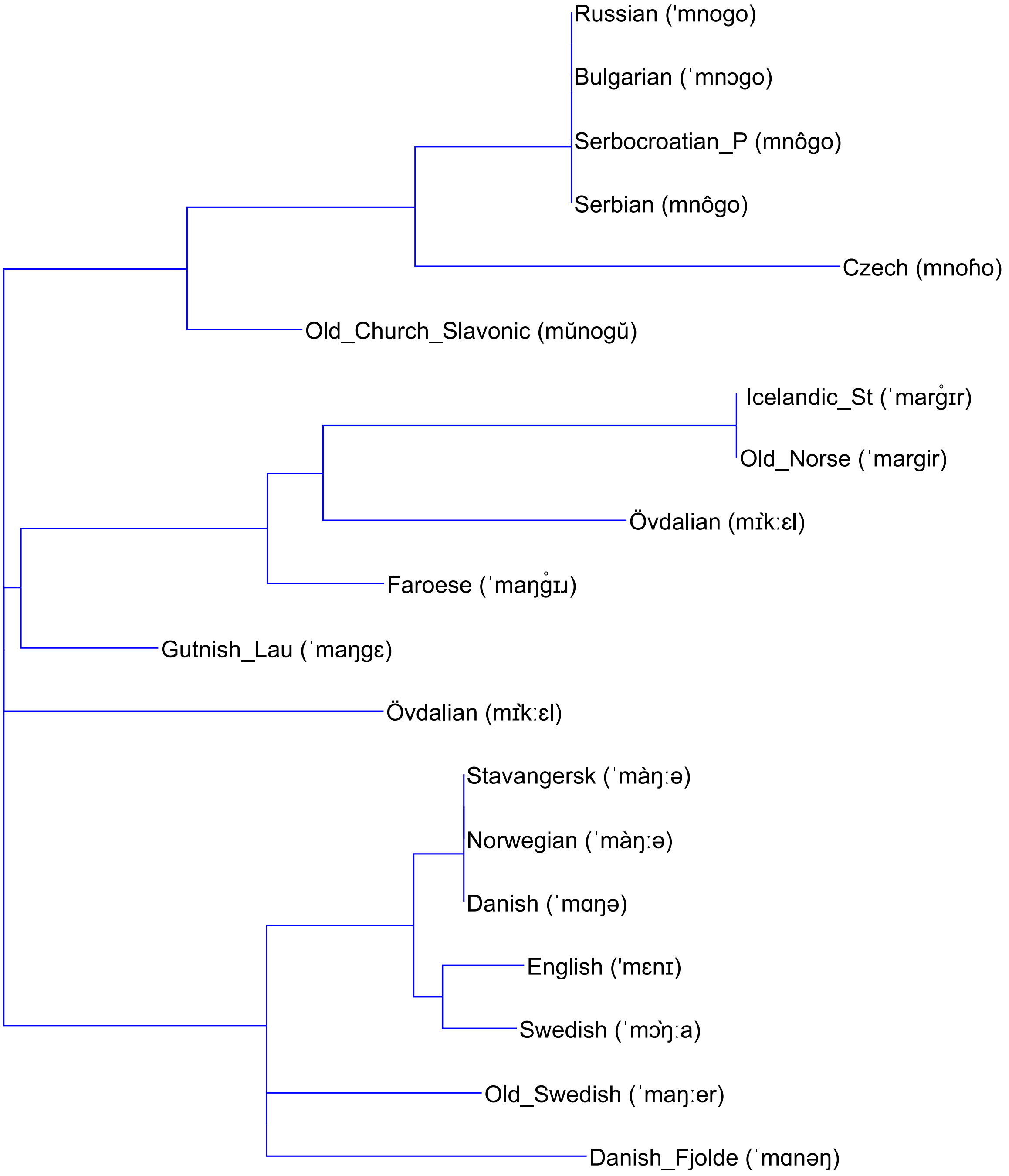

Russian ('mnogo)
Bulgarian (ˈmnɔgo)
Serbocroatian_P (mnôgo)
Serbian (mnôgo)
Czech (mnoɦo)
Old_Church_Slavonic (mŭnogŭ)
Icelandic_St (ˈmarg̊ɪr)
Old_Norse (ˈmargir)
Övdalian (mɪ̀k:ɛl)
Faroese (ˈmaŋg̊ɹ)
Gutnish_Lau (ˈmaŋgɛ)
Övdalian (mɪ̀k:ɛl)
Stavangersk (ˈmàŋ:ə)
Norwegian (ˈmàŋ:ə)
Danish (ˈmɑŋə)
English ('mɛnɪ)
Swedish (ˈmɔ̀ŋ:a)
Old_Swedish (ˈmaŋ:er)
Danish_Fjolde (ˈmɑnəŋ)

Many

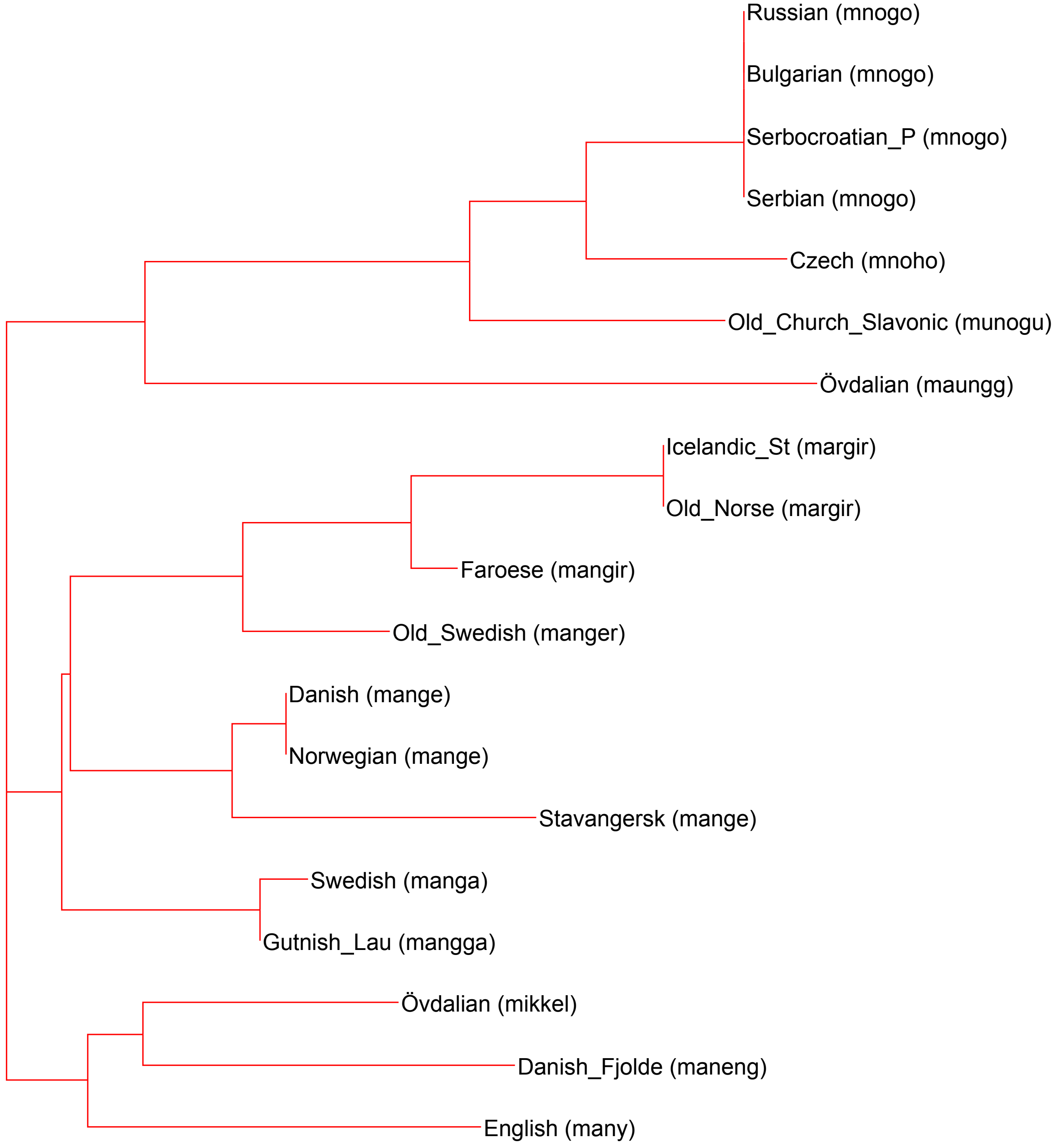

Russian (mnogo)
Bulgarian (mnogo)
Serbocroatian_P (mnogo)
Serbian (mnogo)
Czech (mnoho)
Old_Church_Slavonic (munogu)
Övdalian (maungg)
Icelandic_St (margir)
Old_Norse (margir)
Faroese (mangir)
Old_Swedish (manger)
Danish (mange)
Norwegian (mange)
Stavangersk (mange)
Swedish (manga)
Gutnish_Lau (mangga)
Övdalian (mikkel)
Danish_Fjolde (maneng)
English (many)

Mountain

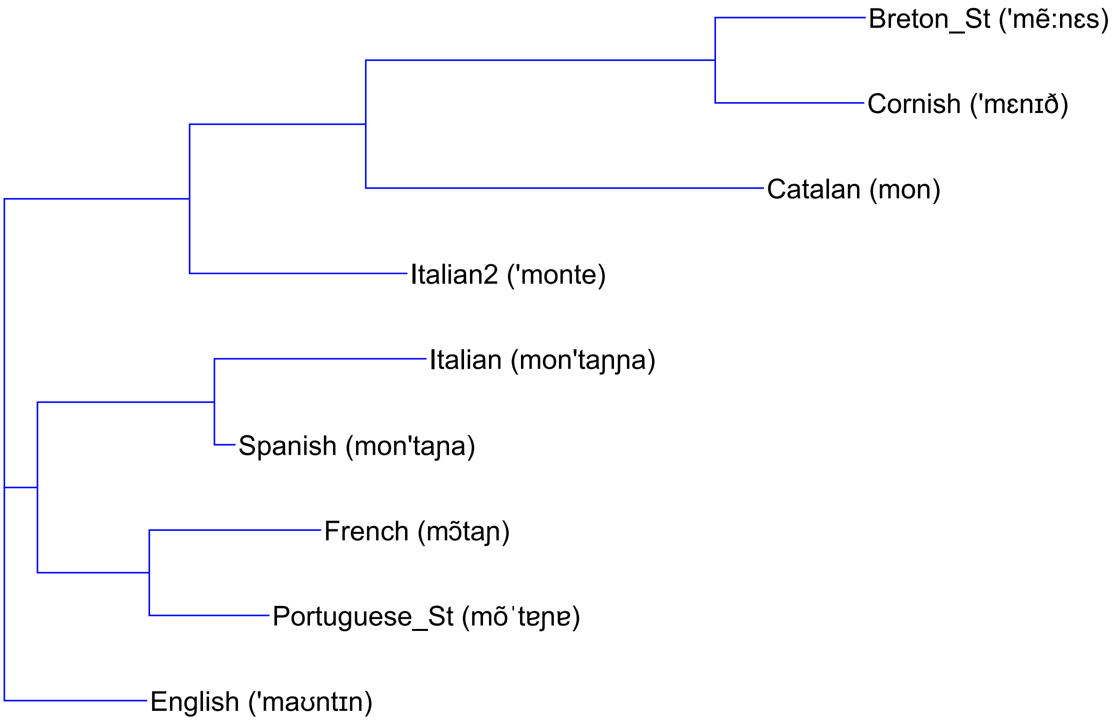

Breton_St ('mẽ:nɛs)
Cornish ('mɛnɪð)
Catalan (mon)
Italian2 ('monte)
Italian (mon'taɲɲa)
Spanish (mon'taɲa)
French (mɔ̃taɲ)
Portuguese_St (mõˈtɐɲɐ)
English ('maʊntɪn)

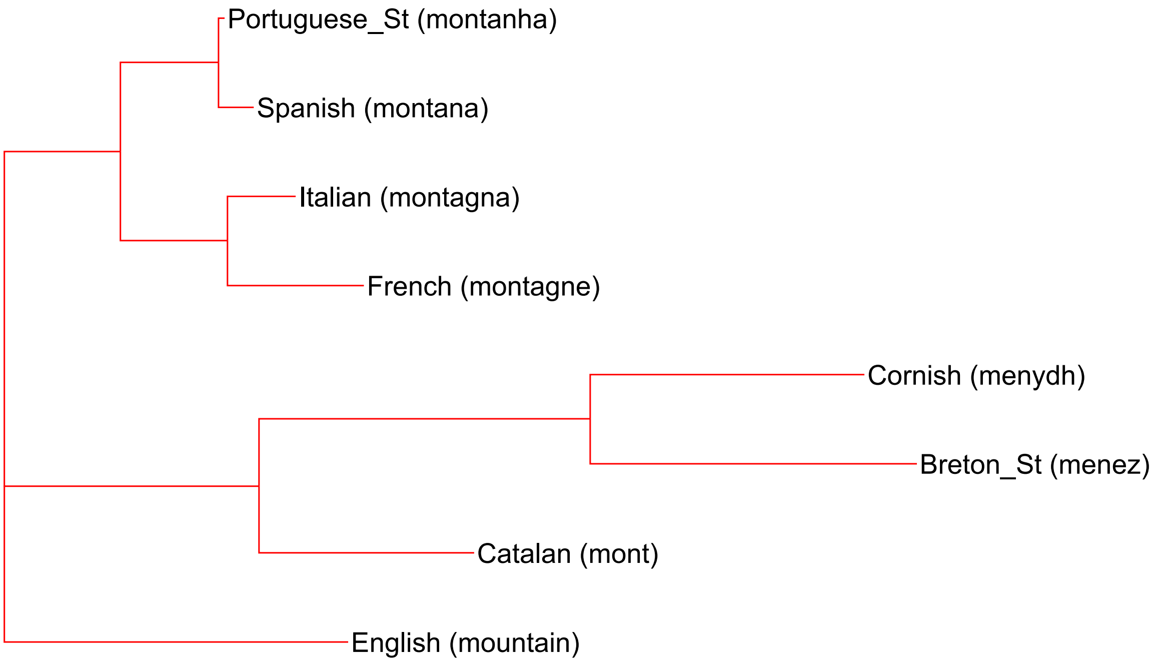

Portuguese_St (montanha)
Spanish (montana)
Italian (montagna)
French (montagne)
Cornish (menydh)
Breton_St (menez)
Catalan (mont)
English (mountain)

Person

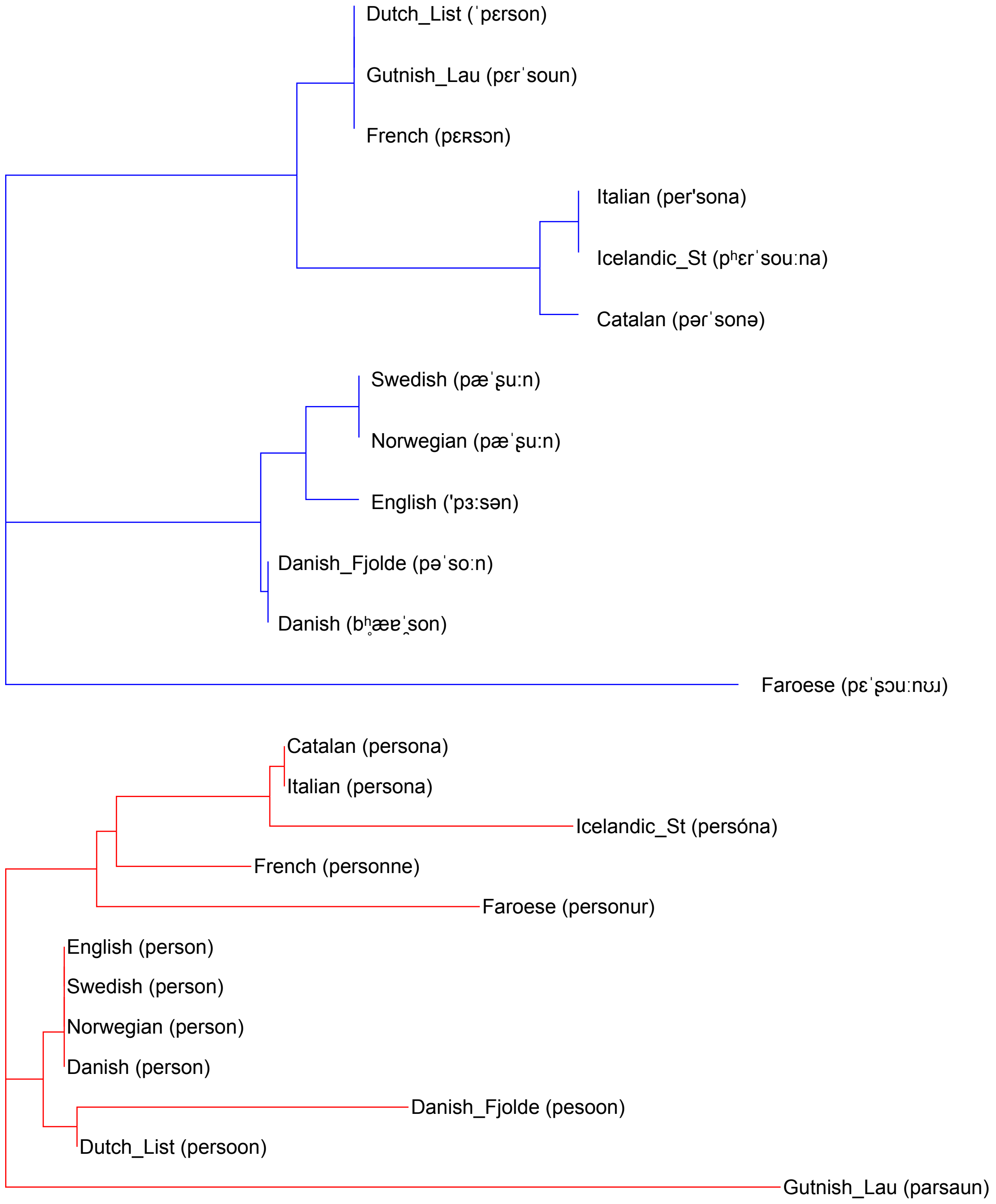
Dutch_List (ˈpɛrson)
Gutnish_Lau (pɛrˈsoun)
French (pɛʁsɔn)
Italian (per'sona)
Icelandic_St (pʰɛrˈsou:na)
Catalan (pərˈsonə)
Swedish (pæˈʂu:n)
Norwegian (pæˈʂu:n)
English ('pɜ:sən)
Danish_Fjolde (pəˈso:n)
Danish (bʰ̥æɐ̯ˈson)
Faroese (pɛˈʂɔu:nʊɹ)
Catalan (persona)
Italian (persona)
Icelandic_St (persóna)
French (personne)
Faroese (personur)
English (person)
Swedish (person)
Norwegian (person)
Danish (person)
Danish_Fjolde (pesoon)
Dutch_List (persoon)
Gutnish_Lau (parsaun)

River

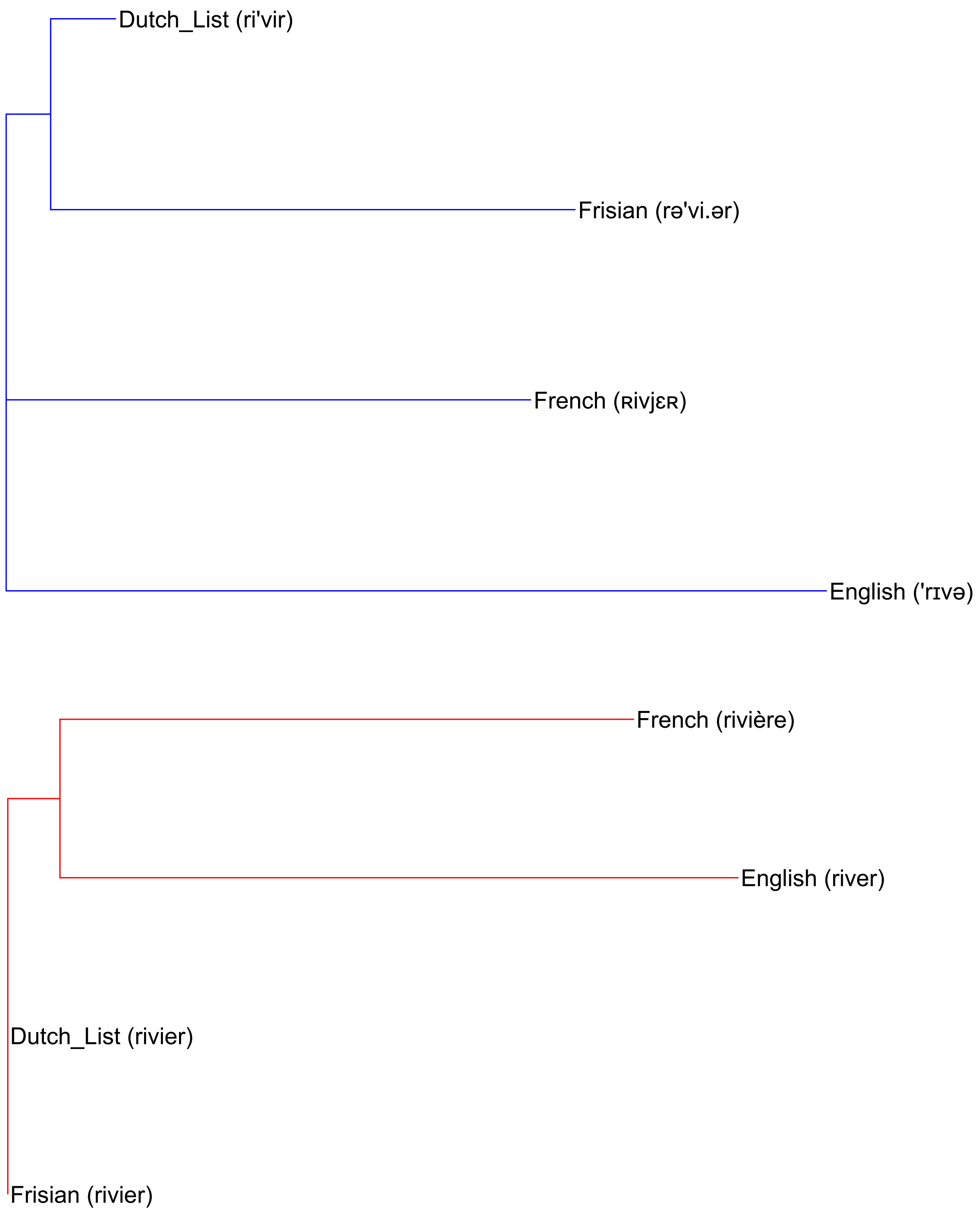
Dutch_List (ri'vir)
Frisian (rə'vi.ər)
French (ʀivjɛʀ)
English ('rɪvə)
French (rivière)
English (river)
Dutch_List (rivier)
Frisian (rivier)

Root

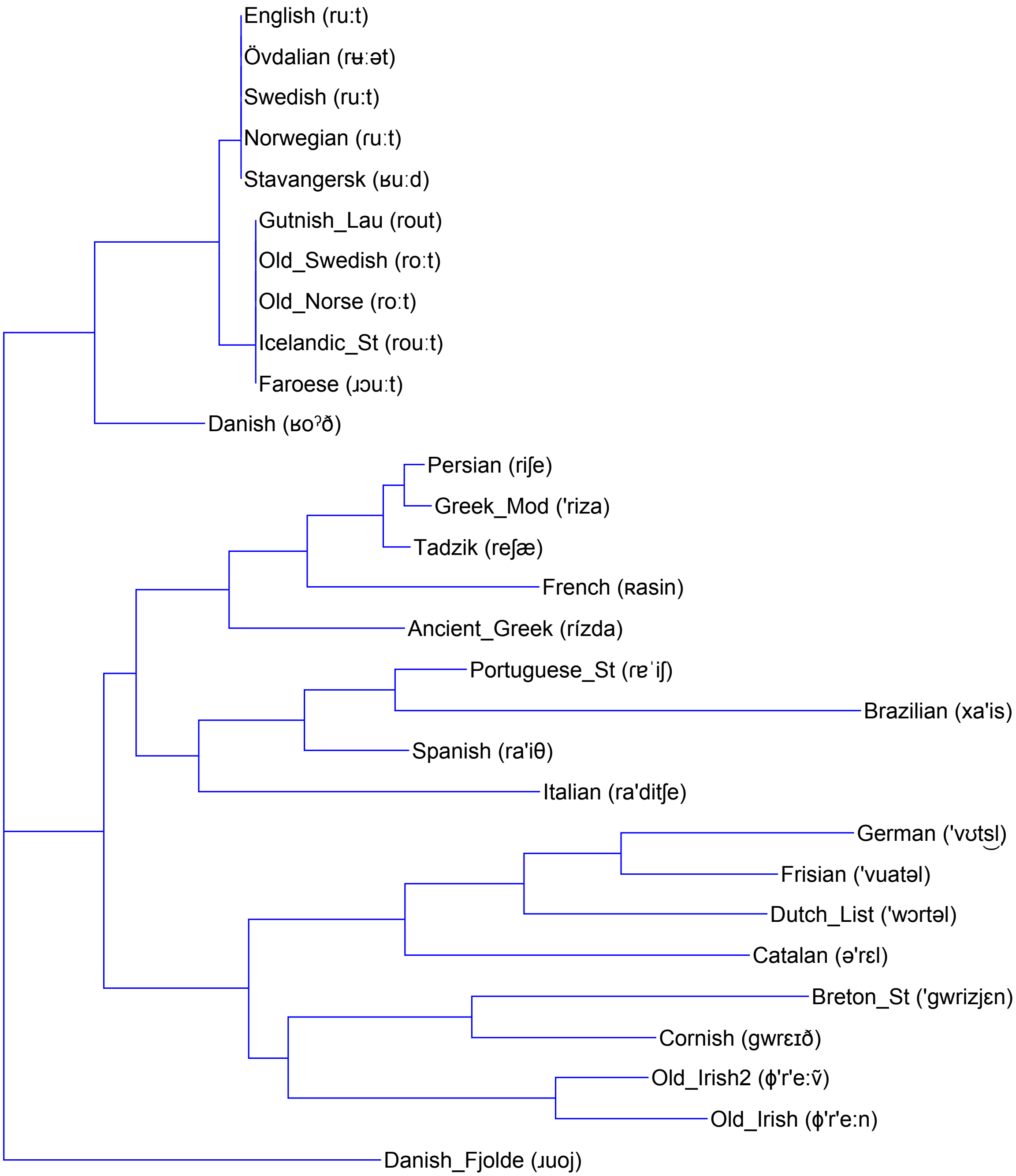
English (ru:t)
Övdalian (rʉ:ət)
Swedish (ru:t)
Norwegian (ru:t)
Stavangersk (ʁu:d)
Gutnish_Lau (rout)
Old_Swedish (ro:t)
Old_Norse (ro:t)
Icelandic_St (rou:t)
Faroese (ɹɔu:t)
Danish (ʁoˀð)
Persian (riʃe)
Greek_Mod ('riza)
Tadzik (reʃæ)
French (ʀasin)
Ancient_Greek (rízda)
Portuguese_St (rɐˈiʃ)
Brazilian (xa'is)
Spanish (ra'iθ)
Italian (ra'ditʃe)
German ('vʊtsl̮)
Frisian ('vuatəl)
Dutch_List ('wɔrtəl)
Catalan (ə'rɛl)
Breton_St ('gwrizjɛn)
Cornish (gwrɛɪð)
Old_Irish2 (ɸ'r'e:ṽ)
Old_Irish (ɸ'r'e:n)
Danish_Fjolde (ɹuoj)

Root

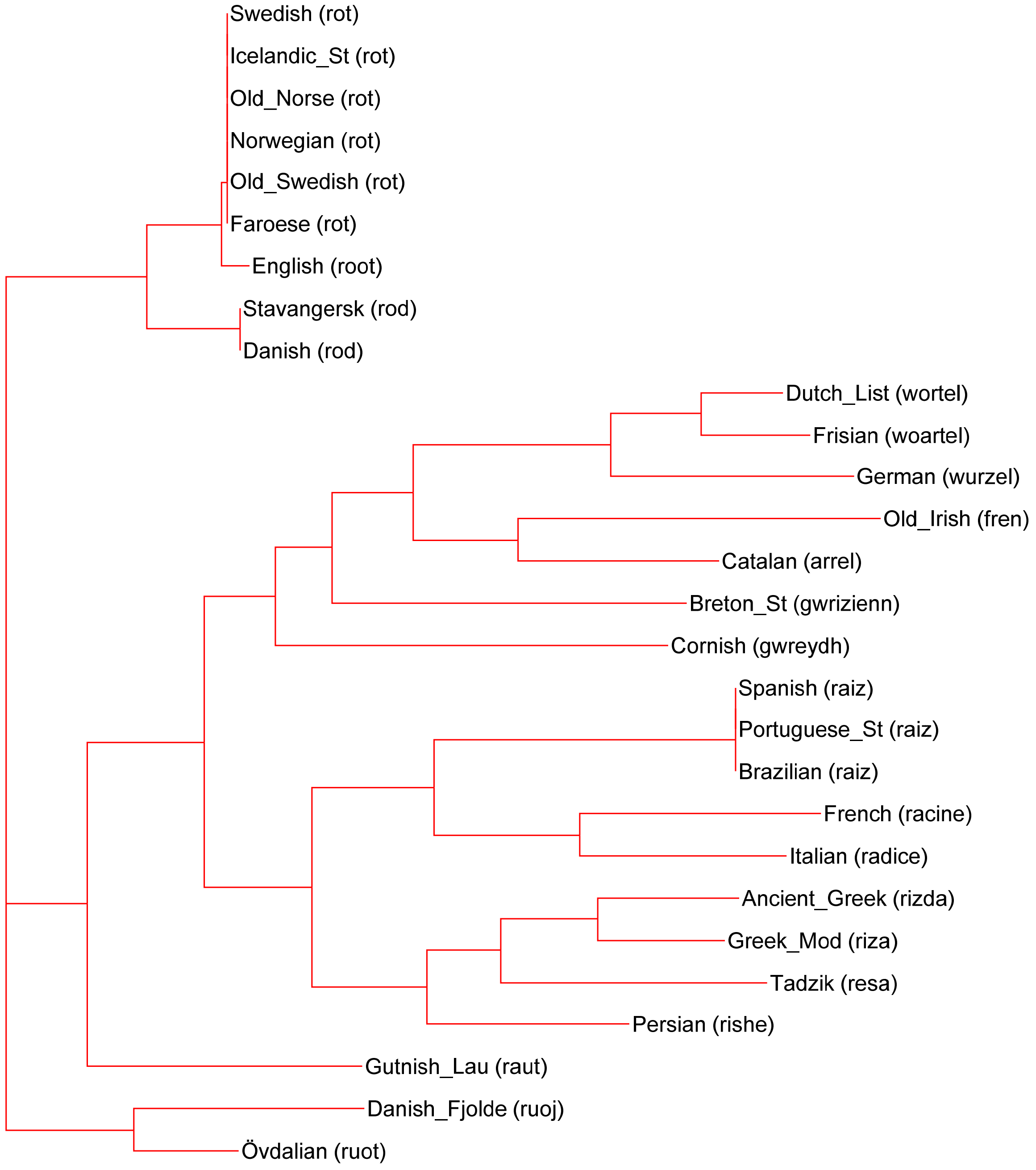
Swedish (rot)
Icelandic_St (rot)
Old_Norse (rot)
Norwegian (rot)
Old_Swedish (rot)
Faroese (rot)
English (root)
Stavangersk (rod)
Danish (rod)
Dutch_List (wortel)
Frisian (woartel)
German (wurzel)
Old_Irish (fren)
Catalan (arrel)
Breton_St (gwrizienn)
Cornish (gwreydh)
Spanish (raiz)
Portuguese_St (raiz)
Brazilian (raiz)
French (racine)
Italian (radice)
Ancient_Greek (rizda)
Greek_Mod (riza)
Tadzik (resa)
Persian (rishe)
Gutnish_Lau (raut)
Danish_Fjolde (ruoj)
Övdalian (ruot)

Skin

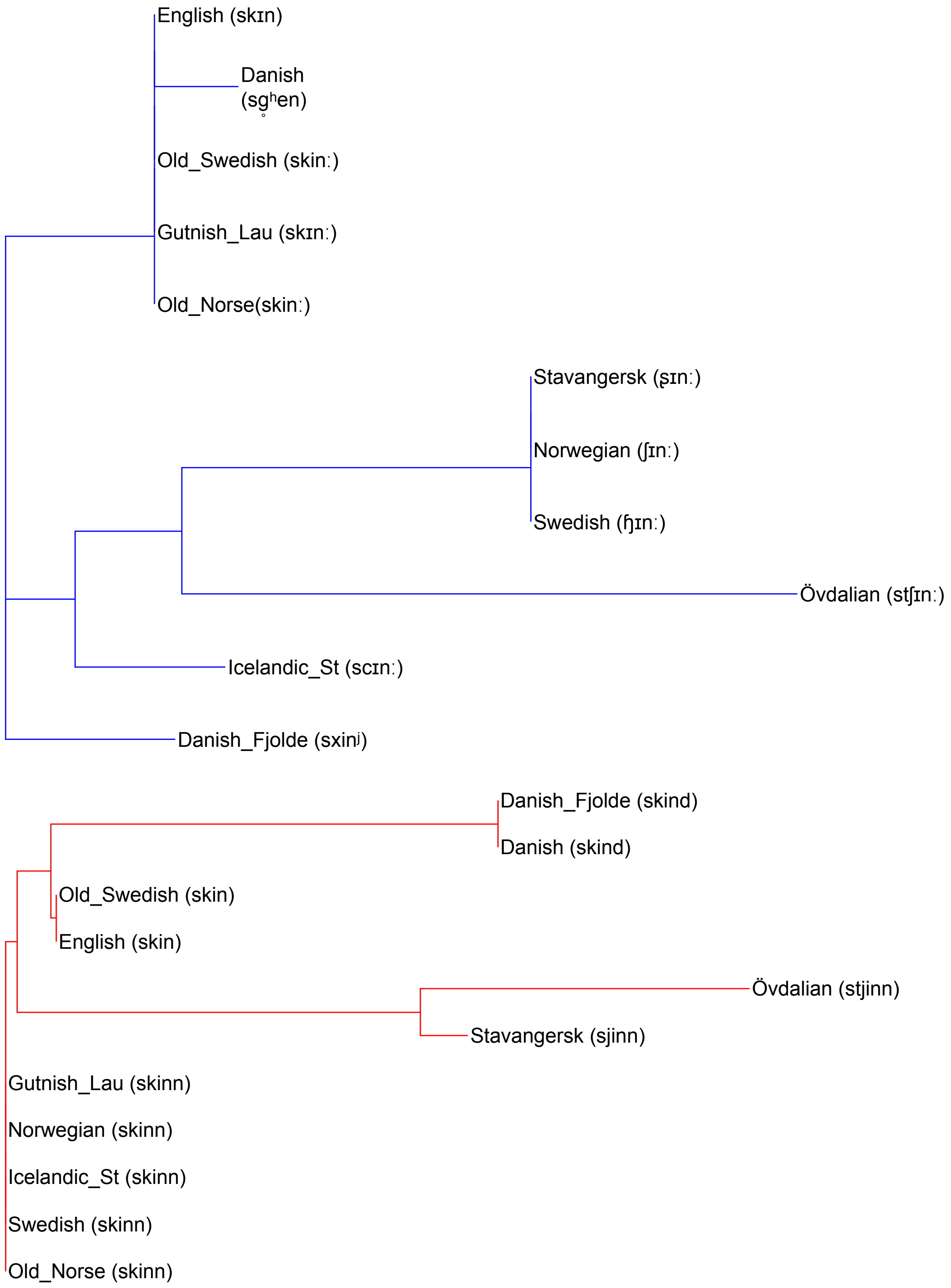
English (skɪn)
Danish (sg̊ʰen)
Old_Swedish (skiːn)
Gutnish_Lau (skɪːn)
Old_Norse(skiːn)
Stavangersk (ʂɪːn)
Norwegian (ʃɪːn)
Swedish (ɧɪːn)
Övdalian (stʃɪːn)
Icelandic_St (scɪːn)
Danish_Fjolde (sxinʲ)
Danish_Fjolde (skind)
Danish (skind)
Old_Swedish (skin)
English (skin)
Övdalian (stjinn)
Stavangersk (sjinn)
Gutnish_Lau (skinn)
Norwegian (skinn)
Icelandic_St (skinn)
Swedish (skinn)
Old_Norse (skinn)

Smoke

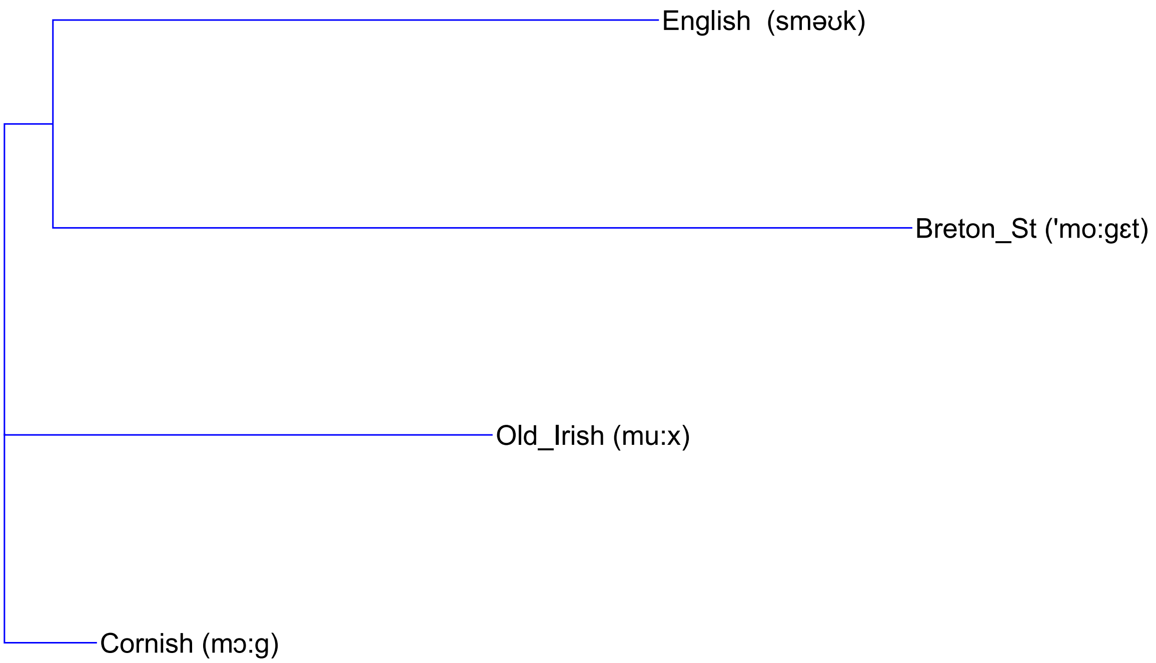
English (sməʊk)
Breton_St ('mo:gɛt)
Old_Irish (mu:x)
Cornish (mɔ:g)

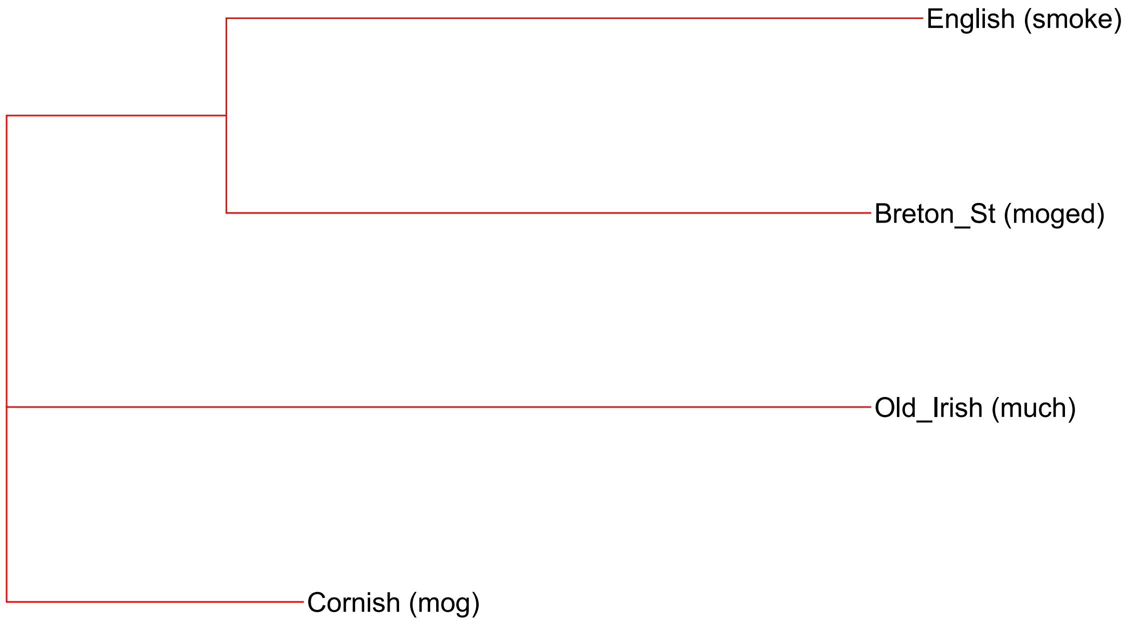
English (smoke)
Breton_St (moged)
Old_Irish (much)
Cornish (mog)

Snake

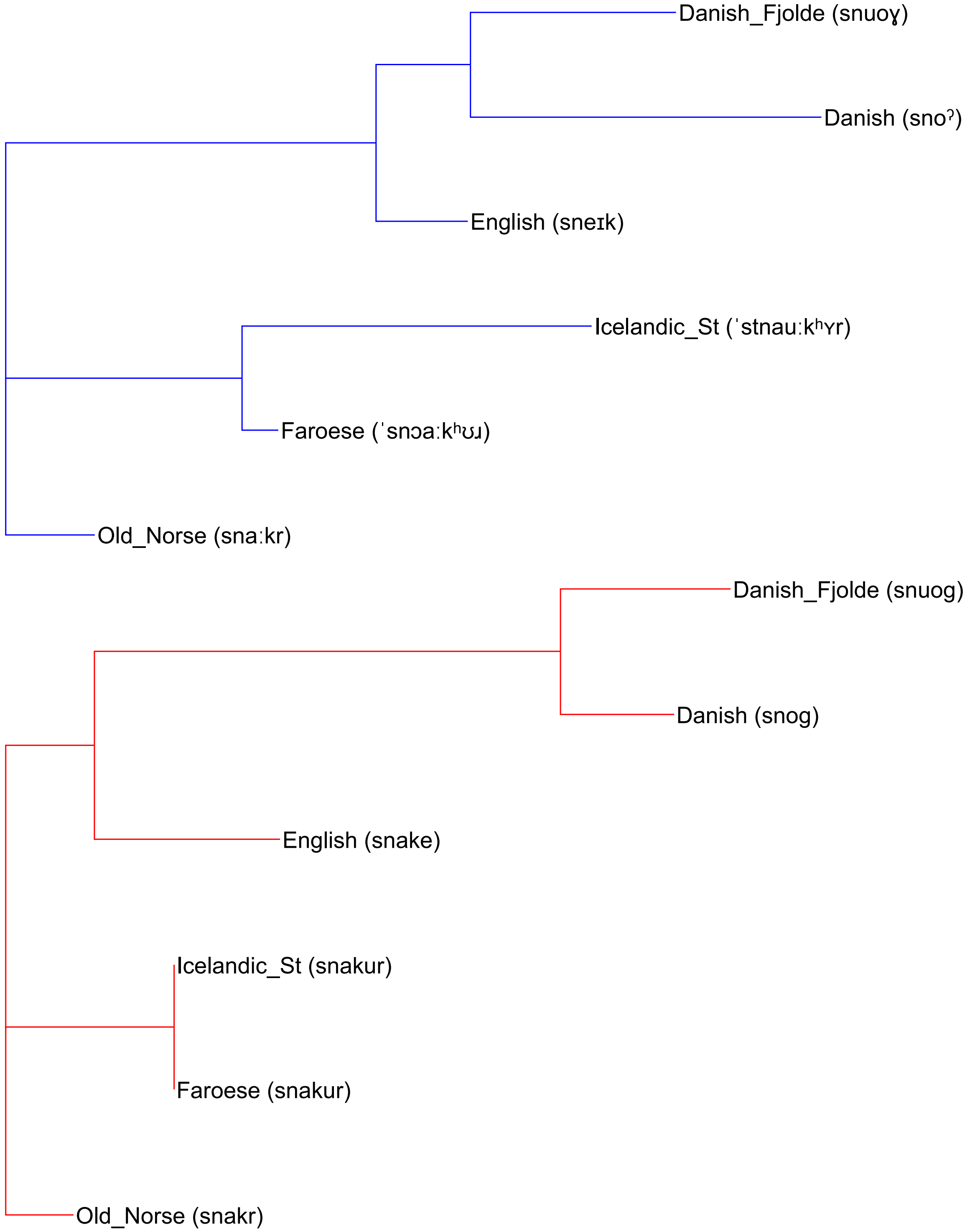

Danish_Fjolde (snuoɣ)
Danish (snoˀ)
English (sneɪk)
Icelandic_St (ˈstnau:kʰʏr)
Faroese (ˈsnɔa:kʰʊɹ)
Old_Norse (sna:kr)
Danish_Fjolde (snuog)
Danish (snog)
English (snake)
Icelandic_St (snakur)
Faroese (snakur)
Old_Norse (snakr)

Split

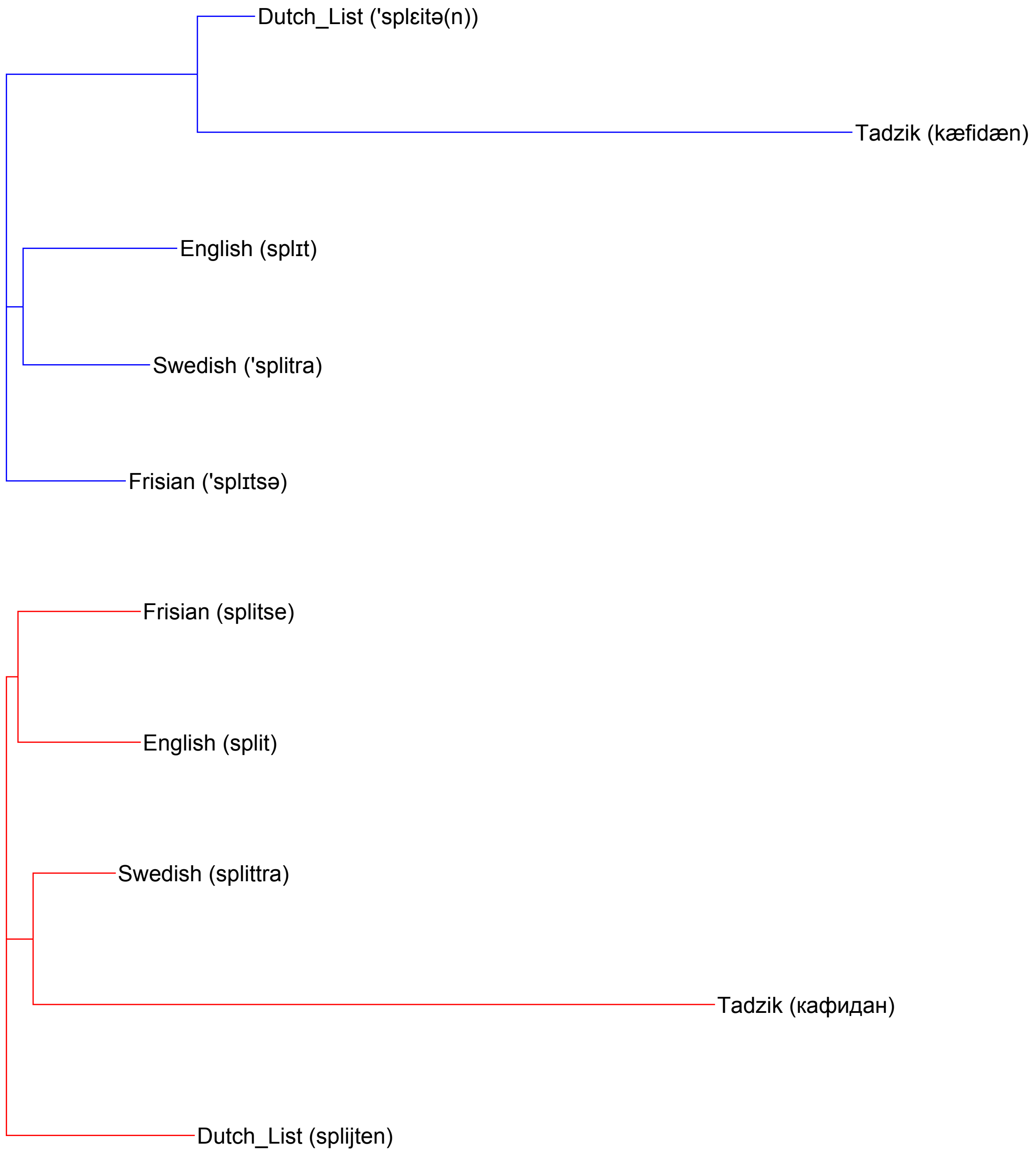
Dutch_List ('splɛitə(n))
Tadzik (kæfidæn)
English (splɪt)
Swedish ('splitra)
Frisian ('splɪtsə)
Frisian (splitse)
English (split)
Swedish (splittra)
Tadzik (кафидан)
Dutch_List (splijten)

Stick

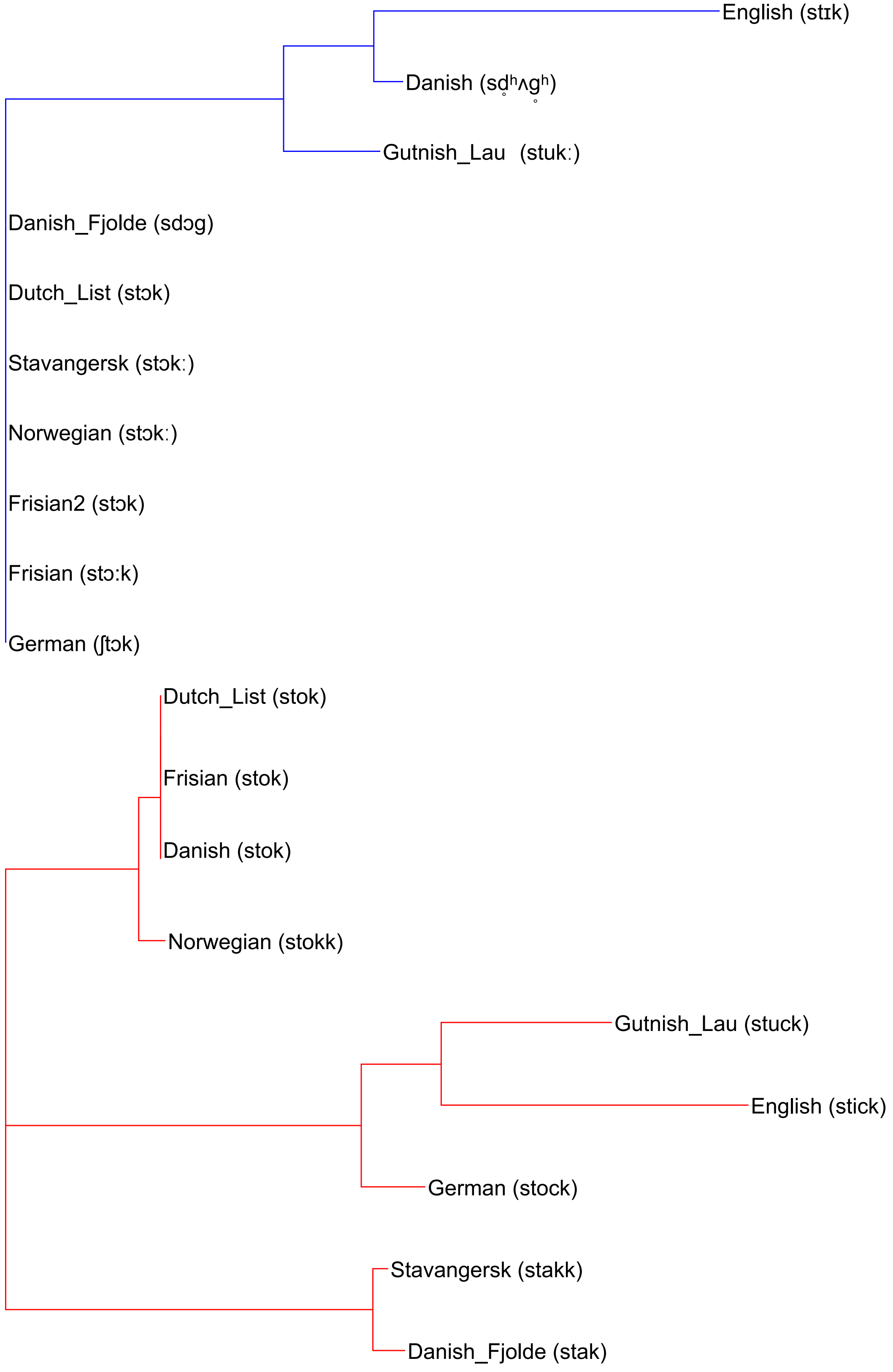

English (stɪk)
Danish (sd̥ʰʌg̊ʰ)
Gutnish_Lau (stuk:)
Danish_Fjolde (sdɔg)
Dutch_List (stɔk)
Stavangersk (stɔk:)
Norwegian (stɔk:)
Frisian2 (stɔk)
Frisian (stɔ:k)
German (ʃtɔk)
Dutch_List (stok)
Frisian (stok)
Danish (stok)
Norwegian (stokk)
Gutnish_Lau (stuck)
English (stick)
German (stock)
Stavangersk (stakk)
Danish_Fjolde (stak)

They

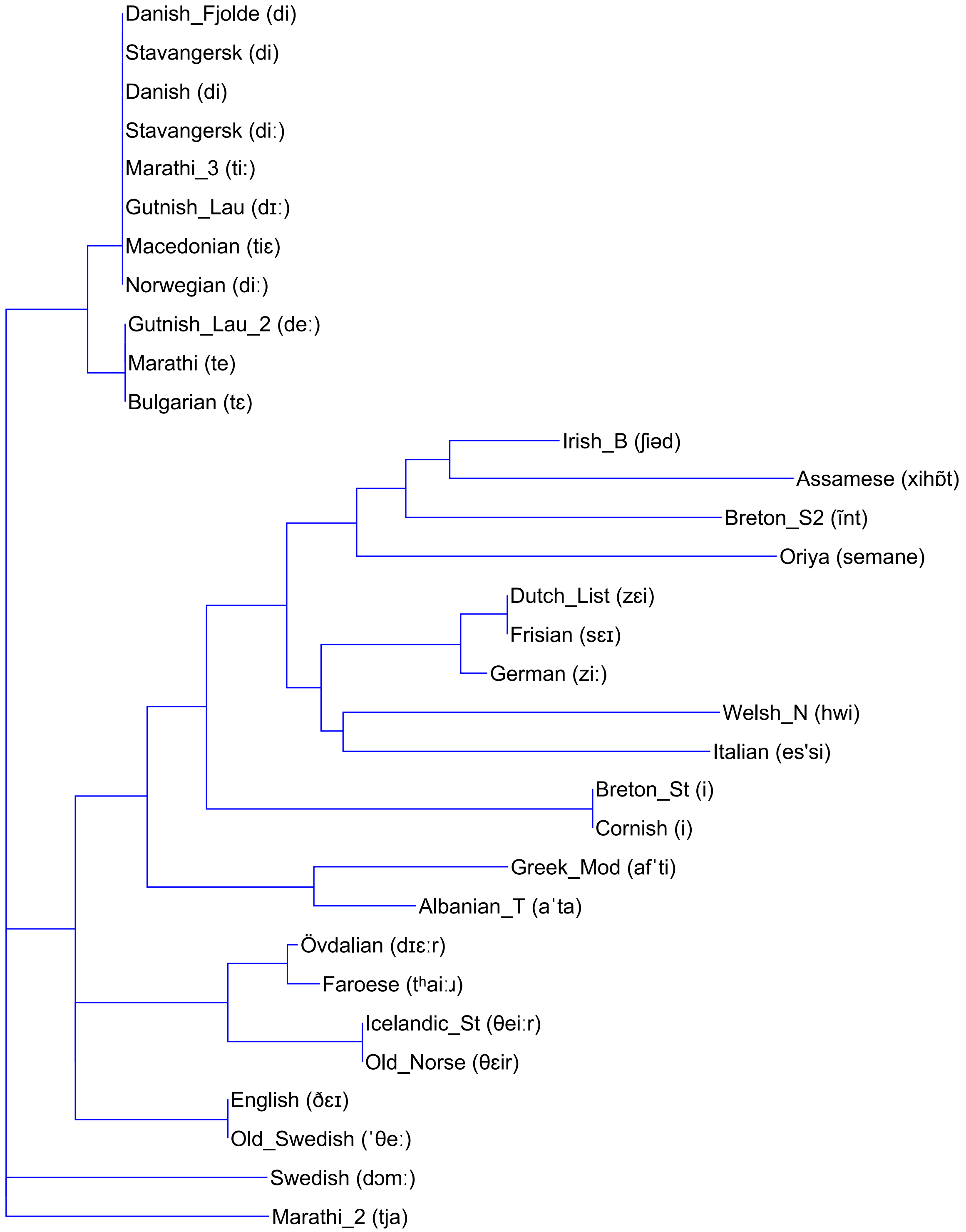
Danish_Fjolde (di)
Stavangersk (di)
Danish (di)
Stavangersk (diː)
Marathi_3 (tiː)
Gutnish_Lau (dɪː)
Macedonian (tiɛ)
Norwegian (diː)
Gutnish_Lau_2 (deː)
Marathi (te)
Bulgarian (tɛ)
Irish_B (ʃiəd)
Assamese (xihõt)
Breton_S2 (ĩnt)
Oriya (semane)
Dutch_List (zɛi)
Frisian (sɛɪ)
German (ziː)
Welsh_N (hwi)
Italian (es'si)
Breton_St (i)
Cornish (i)
Greek_Mod (afˈti)
Albanian_T (aˈta)
Övdalian (dɪɛːr)
Faroese (tʰaiːɹ)
Icelandic_St (θeiːr)
Old_Norse (θɛir)
English (ðɛɪ)
Old_Swedish (ˈθeː)
Swedish (dɔmː)
Marathi_2 (tja)

They

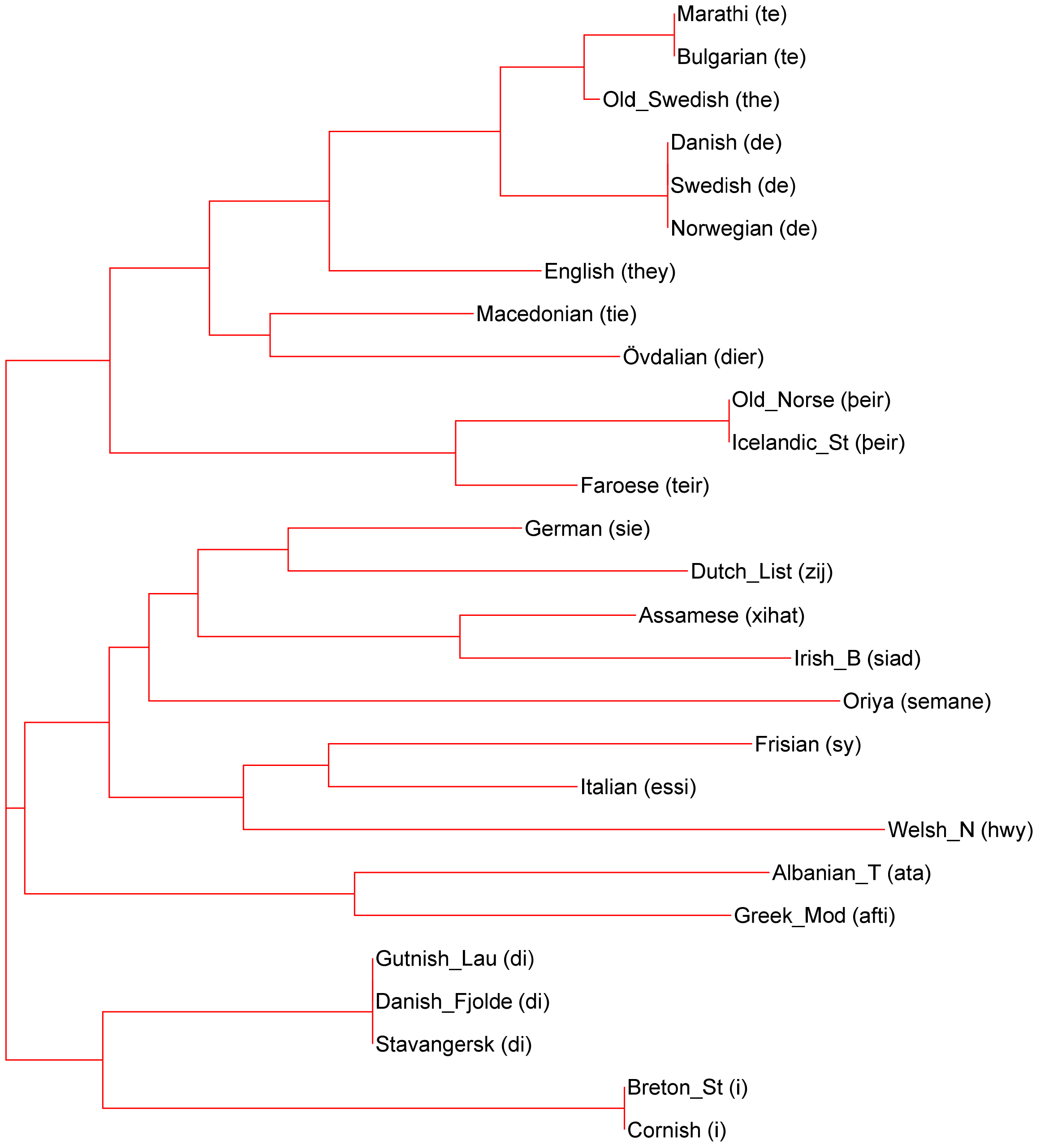
Marathi (te)
Bulgarian (te)
Old_Swedish (the)
Danish (de)
Swedish (de)
Norwegian (de)
English (they)
Macedonian (tie)
Övdalian (dier)
Old_Norse (þeir)
Icelandic_St (þeir)
Faroese (teir)
German (sie)
Dutch_List (zij)
Assamese (xihat)
Irish_B (siad)
Oriya (semane)
Frisian (sy)
Italian (essi)
Welsh_N (hwy)
Albanian_T (ata)
Greek_Mod (afti)
Gutnish_Lau (di)
Danish_Fjolde (di)
Stavangersk (di)
Breton_St (i)
Cornish (i)

Tree

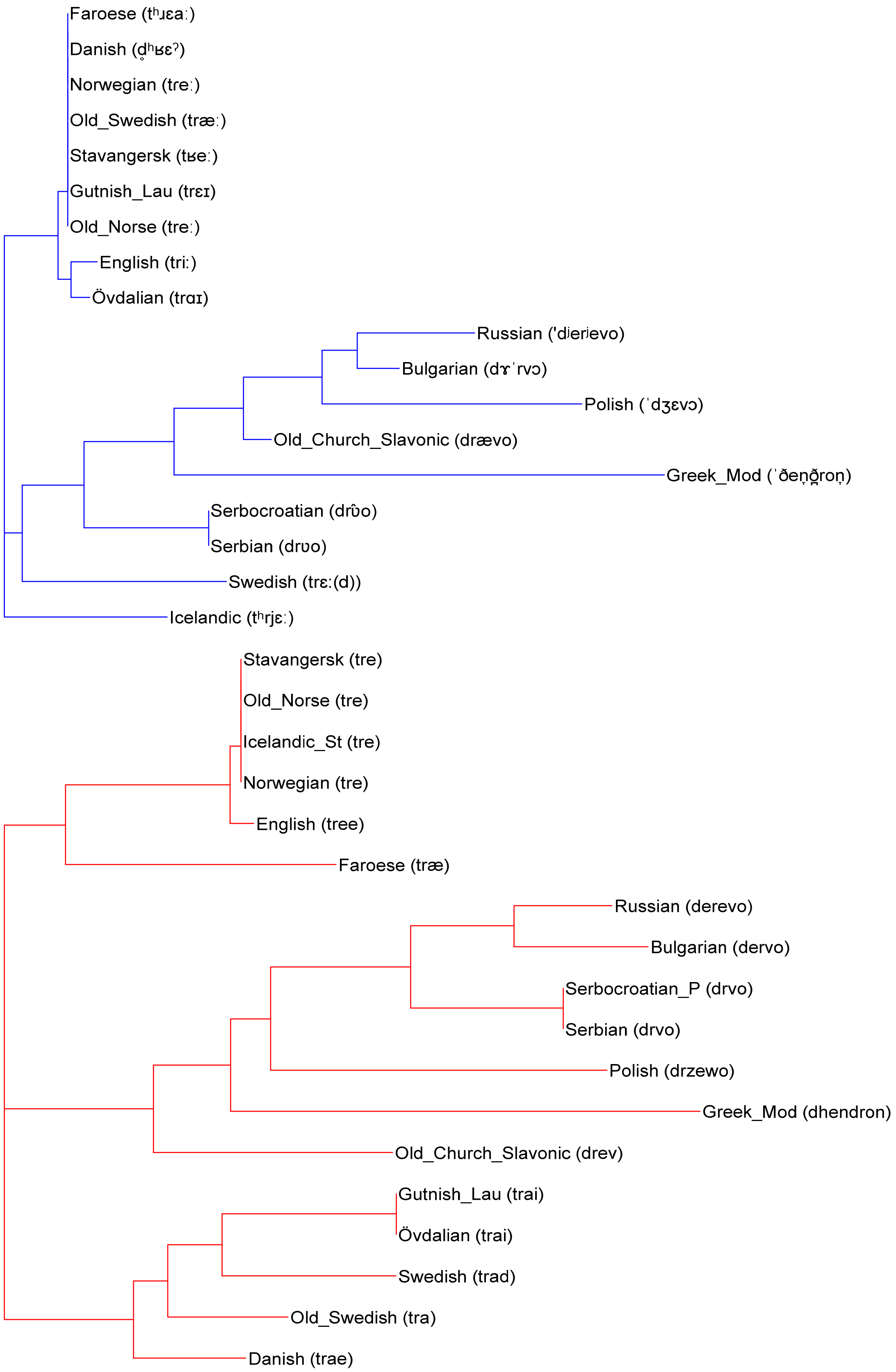
Faroese (tʰɹɛaː)
Danish (d̥ʰʁɛˀ)
Norwegian (treː)
Old_Swedish (træː)
Stavangersk (tʁeː)
Gutnish_Lau (trɛɪ)
Old_Norse (treː)
English (triː)
Övdalian (trɑɪ)
Russian ('dʲerʲevo)
Bulgarian (dɤˈrvɔ)
Polish (ˈdʒɛvɔ)
Old_Church_Slavonic (drævo)
Greek_Mod (ˈðeṇð̪roṇ)
Serbocroatian (drȕo)
Serbian (drʊo)
Swedish (trɛː(d))
Icelandic (tʰrjɛː)
Stavangersk (tre)
Old_Norse (tre)
Icelandic_St (tre)
Norwegian (tre)
English (tree)
Faroese (træ)
Russian (derevo)
Bulgarian (dervo)
Serbocroatian_P (drvo)
Serbian (drvo)
Polish (drzewo)
Greek_Mod (dhendron)
Old_Church_Slavonic (drev)
Gutnish_Lau (trai)
Övdalian (trai)
Swedish (trad)
Old_Swedish (tra)
Danish (trae)

Vomit

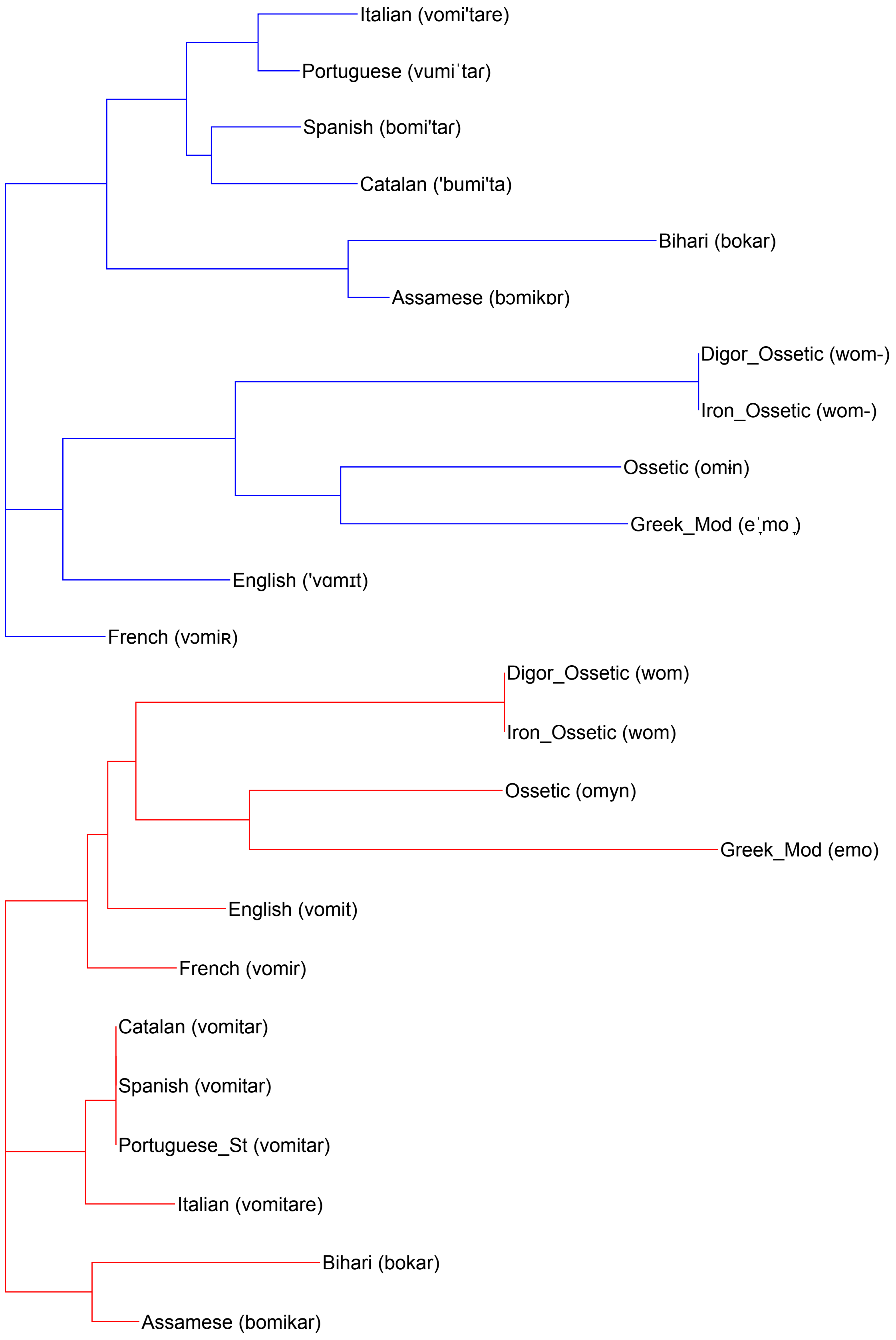
Italian (vomi'tare)
Portuguese (vumiˈtar)
Spanish (bomi'tar)
Catalan ('bumi'ta)
Bihari (bokar)
Assamese (bɔmikɒr)
Digor_Ossetic (wom-)
Iron_Ossetic (wom-)
Ossetic (omɨn)
Greek_Mod (eˈmo)
English ('vɑmɪt)
French (vɔmiʀ)
Digor_Ossetic (wom)
Iron_Ossetic (wom)
Ossetic (omyn)
Greek_Mod (emo)
English (vomit)
French (vomir)
Catalan (vomitar)
Spanish (vomitar)
Portuguese_St (vomitar)
Italian (vomitare)
Bihari (bokar)
Assamese (bomikar)

Wing

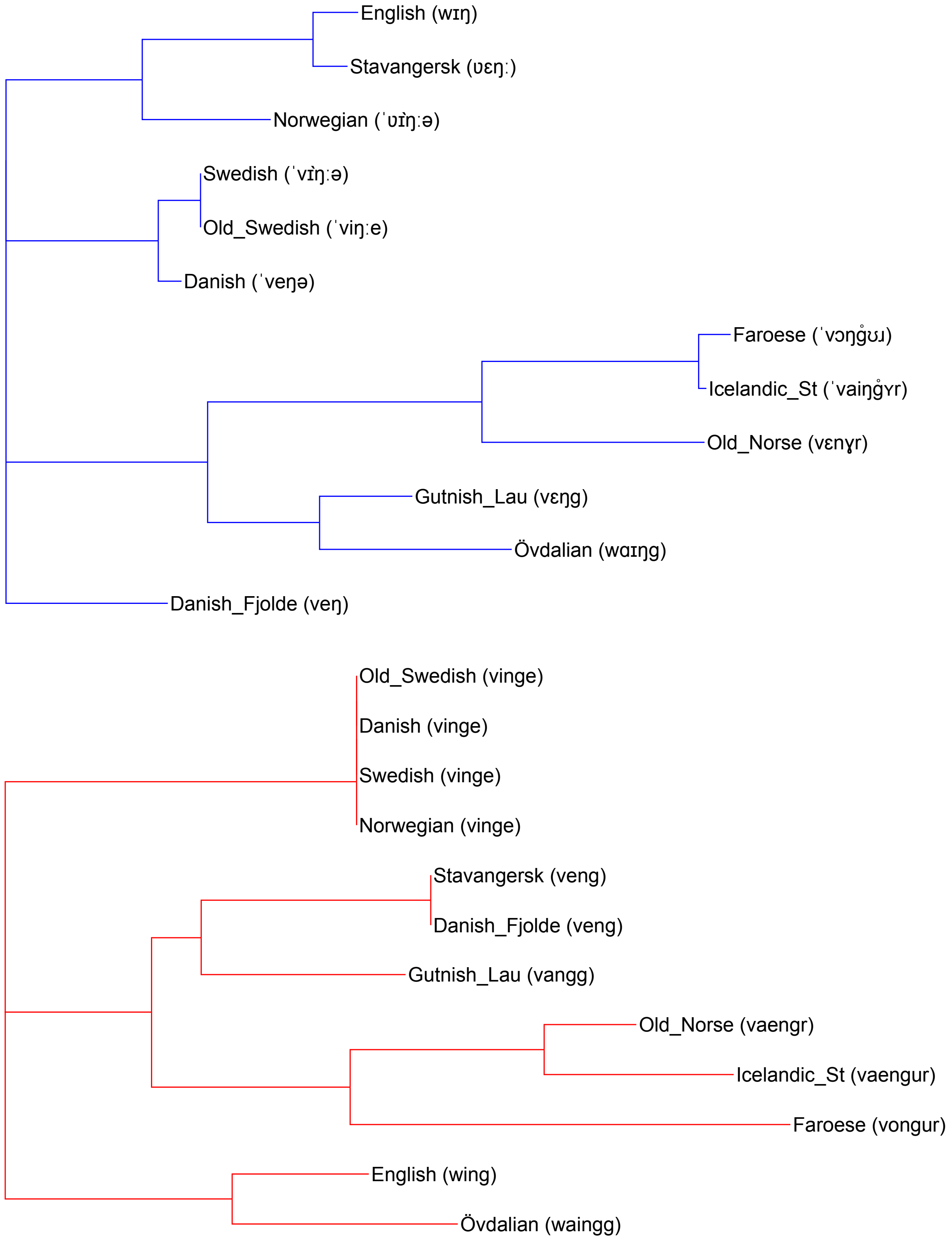
English (wɪŋ)
Stavangersk (ʋɛŋː)
Norwegian (ˈʋɪ̀ŋːə)
Swedish (ˈvɪ̀ŋːə)
Old_Swedish (ˈviŋːe)
Danish (ˈveŋə)
Faroese (ˈvɔŋg̊ʊɹ)
Icelandic_St (ˈvaiŋg̊ʏr)
Old_Norse (vɛnɣr)
Gutnish_Lau (vɛŋg)
Övdalian (waɪŋg)
Danish_Fjolde (veŋ)
Old_Swedish (vinge)
Danish (vinge)
Swedish (vinge)
Norwegian (vinge)
Stavangersk (veng)
Danish_Fjolde (veng)
Gutnish_Lau (vangg)
Old_Norse (vaengr)
Icelandic_St (vaengur)
Faroese (vongur)
English (wing)
Övdalian (waingg)
